# Regulation of Type VI Secretion Systems of *Burkholderia* during transition to intracellular lifestyle

**DOI:** 10.1038/s44319-026-00905-2

**Published:** 2026-09-02

**Authors:** Hoi Ching Cheung, Daria Jacqueline Wüst, Miro Thorsten Wilhelm Plum, Patricia Reist Iscar, Marek Basler

**Affiliations:** 1 Biozentrum, University of Basel, Spitalstrasse 41, Basel, Switzerland; 2 Present Address: Institute of Bioengineering, School of Life Sciences, École Polytechnique Fédérale de Lausanne, Lausanne, Switzerland; 3 Present Address: University of Lausanne, Chemin des Boveresses 155, Epalinges, Switzerland

**Keywords:** Autophagy & Cell Death, Microbiology, Virology & Host Pathogen Interaction, Organelles

## Abstract

Pathogenic bacteria tightly regulate gene expression in response to environmental cues. In *Burkholderia* species, the Type VI Secretion Systems (T6SS) mediate interbacterial competition and host cell interactions. Here, we show that *B. thailandensis* switches from the anti-bacterial T6SS-1 to the anti-eukaryotic T6SS-5 during phagosomal maturation. While T6SS-1 expression persists in the host cells, its assembly progressively declines with increasing T6SS-5 expression. This switch is controlled by the two-component system VirAG as deletion of *virA* blocks T6SS-5 expression and restores T6SS-1 assembly in host cells. Furthermore, we found that deletion of T6SS-5 membrane complex restores T6SS-1 assembly in host cells. Notably, T6SS-1 activity in host cells induces mitochondrial fragmentation and apoptosis. Our data suggest that *Burkholderia* has evolved a mechanism to silence T6SS-1 upon host entry to preserve its replicative niche.

## Synopsis

*Burkholderia thailandensis* uses VirAG to switch from the T6SS-1 (required for bacterial competition) to the T6SS-5 (cell-cell fusion) during infection of host cells. This reveals coordination of distinct secretion systems during transition from environment to the host.

- During infection, *B. thailandensis* gradually shifts from T6SS-1 assembly to T6SS-5 expression in the phagosome.
- Expression of T6SS-5 inhibits T6SS-1 assembly and firing, revealing cross-regulation between the two secretion systems.
- T6SS-1 activity in host cells induces mitochondrial fragmentation and apoptosis.
- Silencing T6SS-1 is important for maintaining an intracellular replicative niche.

## Abstract (web summary)

*Burkholderia thailandensis* uses VirAG to switch from the T6SS-1 (required for bacterial competition) to the T6SS-5 (cell-cell fusion) during infection of host cells. This reveals coordination of distinct secretion systems during transition from environment to the host.

## Graphical abstract

**Figure:**
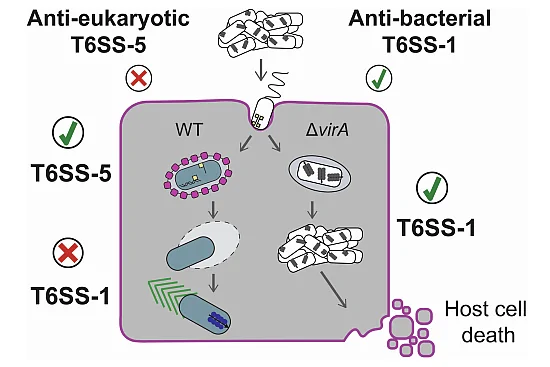

## Introduction

To adapt to various niches, pathogens employ different factors required for virulence and survival in the environment. The expression of these factors is often tightly controlled. The facultative intracellular pathogen *Burkholderia pseudomallei* is endemic in tropical and subtropical regions and is the causative agent of the disease Melioidosis (Sanchez-Villamil et al, 2020; Wiersinga et al, 2006; Meumann et al, 2023; Limmathurotsakul et al, 2010). Patients are commonly infected via inhalation, ingestion or skin abrasions (Barnes and Ketheesan, 2005). It is estimated to have 165,000 melioidosis cases leading to 89,000 deaths per year globally (Birnie et al, 2019; Limmathurotsakul et al, 2016), and the mortality rate ranges from 10 to 40% (Limmathurotsakul et al, 2010; Currie et al, 2021; Chantratita et al, 2023). Due to the disease’s high mortality rate and resistance to antibiotics, *B. pseudomallei* is listed as a tier 1 select agent by the US Centers for Disease Control and Prevention (Rhodes and Schweizer, 2016; Meumann et al, 2023). The closely related, less virulent *Burkholderia thailandensis* is often used as an attenuated model for studying *Burkholderia* virulence (Haraga et al, 2008).

Both *B. pseudomallei* and *B. thailandensis* are able to infect a range of phagocytic and non-phagocytic eukaryotic cells (Whiteley et al, 2017). Upon entry into host cells, *B. thailandensis* escapes the phagosome using effectors of the Type III Secretion System (T3SS_Bsa_) (Stevens et al, 2002). *B. thailandensis* moves in the host cytosol by using an intracellular flagellum and by polymerising host cell actin with the bacterial protein BimA, which is a mimic of the eukaryotic Arp2/3 complex (Stevens et al, 2005). Actin-tails promote formation of membrane protrusions into the neighbouring host cell. In the protrusions, *B. thailandensis* assembles its T6SS-5 to fuse host cell membranes by VgrG-5, leading to a protrusion escape or fusion of host cells (Schwarz et al, 2010, 2014; Plum et al, 2024; Kostow and Welch, 2022; Toesca et al, 2014).

T6SS is structurally homologous to a contractile bacteriophage tail and functions by propelling a needle-like structure with effectors into a neighbouring target cell (Pukatzki et al, 2006; Hood et al, 2010; Ma et al, 2009; Pukatzki et al, 2007; Basler and Mekalanos, 2012). Its core structure consists of a membrane and baseplate complex formed around a VgrG-PAAR spike (Aschtgen et al, 2010; Durand et al, 2015; Brunet et al, 2015; Nazarov et al, 2018; Filloux and Freemont, 2016; Kudryashev et al, 2015; Felisberto-Rodrigues et al, 2011). The sheath is formed by the assembly of TssB-C around an inner tube of Hcp rings starting from the VgrG-PAAR base and extending into the cytoplasm. When the TssB-C sheath contracts, it drives the Hcp-VgrG-PAAR and associated effectors outside the cell and into the target cell (Basler et al, 2012; Szwedziak and Pilhofer, 2019; Shneider et al, 2013; Wood et al, 2019). The contracted sheath is disassembled by the ATPase ClpV (Basler and Mekalanos, 2012; Kapitein et al, 2013; Bönemann et al, 2009; Pietrosiuk et al, 2011).

*B. pseudomallei* and *B. thailandensis* have multiple T6SS with distinct roles and high homology between the two species (Schwarz et al, 2010; Angus et al, 2014). T6SS-5 was shown to be important for cell-to-cell spread (Schwarz et al, 2014; Toesca et al, 2014; Plum et al, 2024; Schell et al, 2007; Lennings et al, 2019); whereas T6SS-1 is required for bacterial killing (Schwarz et al, 2010; Lennings et al, 2019; Majerczyk et al, 2016). In previous work, functional chromosomal fusion of the sheath protein TssB of both T6SS-1 and T6SS-5 with fluorophores have been generated (Plum et al, 2024; Lin et al, 2022).

The expression and assembly *B. thailandensis* T6SS-5 are tightly regulated. The regulatory cascade starts with BsaN, a central regulator located in the T3SS_Bsa_ locus. It regulates T3SS effectors and two downstream T6SS-5 regulators VirAG and BprC (Chen et al, 2011, 2014). A two-component histidine kinase, VirAG, regulates T6SS-5 expression during infection; and BprC regulates T6SS-5 expression when grown in medium, except for the *tssBC* operon (Chen et al, 2011). The sensor VirA phosphorylates VirG upon sensing host cytosol when the bacterium escapes from the endosome and the phosphorylated VirG binds to the promoter regions of TssB-5 and Hcp-5 (Wong et al, 2015). However, the signals for BsaN and BprC activation are unknown and the redox signal sensed by VirAG is the only signal identified during infection so far. The regulation of the anti-bacterial T6SS-1 is poorly understood in *B. thailandensis*. It was shown that quorum sensing controls the expression of T6SS-1 effectors and VgrG-1 (Majerczyk et al, 2016). In terms of post-translational regulation of T6SS-1, it was shown that T6SS accessory protein TagM-1 is important for T6SS-1 assemblies (Lin et al, 2022).

In this study, we aim to understand how *Burkholderia* regulates its different T6SS in the environment and during infection. We investigated the expression and assembly of all five T6SS of *B. thailandensis* by live-cell imaging in medium and during infection. Here, the T6SS expression refers to the detection of uniformly distributed fluorescence signal in the bacterial cytosol, while the T6SS assembly refers to the detection of the formation and disassembly of bright foci within bacterial cytosol. We observed that the phagosomal environment is a transition state where there is a gradual increase in T6SS-5 expression and decrease of T6SS-1 assemblies, while deletion of T6SS-5 components restored T6SS-1 assembly. Furthermore, we identified VirAG as a regulator for both T6SS-5 expression and T6SS-1 assemblies. Deletion of *virA* abolished T6SS-5 expression and allowed T6SS-1 assemblies during infection, which led to apoptotic host cell death. This indicates that switch between the two T6SS allows the bacteria to adapt to their environment and to spread during infection.

## Results

### T6SS-2, T6SS-4 and T6SS-6 are not expressed during infection of A549 cells

*B. thailandensis* harbours five T6SS on different chromosomal loci and with different functions. Previous studies have mainly focused on the anti-eukaryotic T6SS-5 as it alone was sufficient to cause virulence in mice (Schwarz et al, 2010). A transcriptomics study has hinted that T6SS-2, T6SS-4, and T6SS-6 have increased expression when inside of host cells (Heacock-Kang et al, 2021). However, it remains unclear if these T6SS are expressed intracellularly. To test this, we constructed strains of *B. thailandensis* with the T6SS sheath proteins TssB tagged with mNeonGreen expressed from the native chromosomal loci. We then infected a monolayer of A549 cells and performed live-cell imaging. Intracellular bacteria were identified by constitutive expression of cytoplasmic mCherry2 under a *Burkholderia* ribosomal promoter, or by expression of the TssB-5-mCherry2 fusion protein. Images were acquired between 11 and 12 h post-infection (hpi) to localize bacteria in the cytoplasm and in membrane protrusions. We failed to detect expression of T6SS-2, T6SS-4 and T6SS-6 during infection (Appendix Fig. S1A). In accordance with previous studies, the T6SS-5 was expressed in *B. thailandensis* at 12 hpi. Notably, the anti-bacterial T6SS-1 expression was detected during infection (Appendix Fig. S1B).

We next assessed T6SS expression when grown in liquid culture and imaged on LB (Luria–Bertani) agar pads. T6SS-2 and T6SS-4 were not expressed under such conditions as the fluorescently-labelled *B. thailandensis* strains exhibited background fluorescence levels comparable to those of the unlabelled strain. The T6SS-6-labelled strain exhibited heterogeneous expression, but no assemblies were detected (Appendix Fig. S1C).

### T6SS-5 is repressed in vitro and induced within host cells before phagosomal escape

The anti-eukaryotic T6SS-5 was shown to be expressed when *B. thailandensis* is in the host cell cytosol, however, T6SS-5 expression dynamics has never been analyzed (Wong et al, 2015). In accordance with previous studies (Chen et al, 2011), we detected no expression of T6SS-5 when *B. thailandensis tssB-5-mScarlet-I* was imaged in vitro on agar pads while its expression was induced by expressing VirAG from an arabinose-inducible plasmid (Appendix Fig. S1D).

**Figure 1:**
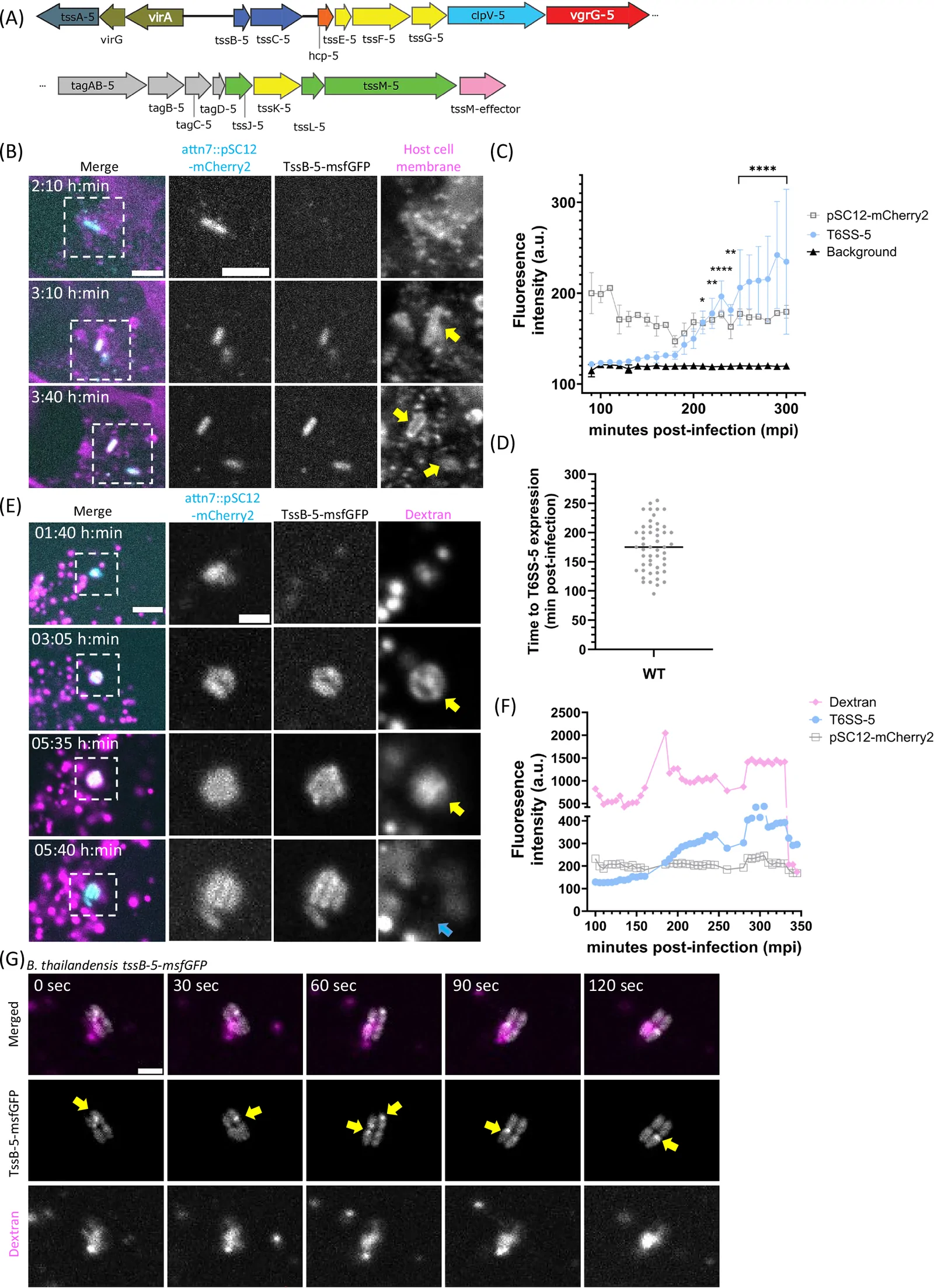
Expression and assembly of T6SS-5 and its regulatory component VirAG inside primary vacuole. (**A**) Scheme of the anti-eukaryotic T6SS-5 genetic cluster. (**B**) Time-lapse imaging of A549 cells infected with *B. thailandensis* expressing cytosolic mCherry2 under ribosomal promoter pSC12 (cyan) and TssB-5-msfGFP (grey) at MOI of 50. Host cell membrane was stained with CellMask deep red (magenta). Yellow arrows indicate host cell membrane around the bacteria. Scale bars represent 5 µm. Images are representative of at least three biological replicates. (**C**) Fluorescence intensity of various channels during the 5-h infection period shown in (**B**). Bacteria were segmented and tracked, and fluorescence intensities of TssB-5-msfGFP (T6SS-5, in blue), bacterial cytosol (pSC12-mCherry2, in grey), and background (black) were plotted. A two-way ANOVA with Tukey’s multiple comparisons test was performed (*P < 0.05, **P < 0.01, ***P < 0.001). Adjusted *P* values for the comparison between reporter and background signals at each time point were: 210 min, *P* = 0.0109; 220 min, *P* = 0.0014; 230 min, *P* = 1.88 × 10⁻⁵; 240 min, *P* = 0.0013; 250 min, *P* = 1.79 × 10⁻⁶; 260 min, *P* = 2.84 × 10⁻⁷; 270 min, *P* = 7.07 × 10⁻⁷; 280 min, *P* = 5.16 × 10⁻⁷; 290 min, *P* = 5.06 × 10⁻⁹; 300 min, *P* = 3.69 ×  10⁻¹⁰. Error bars represent mean ± SD. (**D**) Scatter plot showing the timing of T6SS-5 expression. Each dot represents an individual bacterium (*n* = 50). A549 cells were infected with *B. thailandensis* expressing TssB-5-mScarlet-I at MOI 50-200. Time-lapse images were acquired from 1:30 hpi at either 1 min, 5 min, or 10 min intervals across at least three biological replicates. (**E**) Time-lapse imaging of A549 cells infected with *B. thailandensis* expressing bacterial cytosolic mCherry2 under ribosomal promoter pSC12 (cyan) and TssB-5-msfGFP (grey) at MOI of 200. Host cells were pre-treated with 0.25 mg/mL of fluorescent dextran for 18 h (magenta). Yellow arrows indicate two bacteria expressing T6SS-5 when inside of a dextran-labelled vacuole. Blue arrow indicates the disappearance of the dextran signal. Scale bars represent 5 µm in the overview image and 5 µm in the magnified image. Images are representative of at least three biological replicates. (**F**) Fluorescence intensity of various channels during the 5 h 45 min infection period shown in (**E**). Bacteria were segmented and tracked, and fluorescence intensities of TssB-5-msfGFP (T6SS-5, in blue), bacterial cytosol (pSC12-mCherry2, in grey), and fluorescent dexran (magenta) were plotted. (**G**) *B. thailandensis* assembling T6SS-5 at 3 hpi inside of a dextran-labelled vacuole during infection of A549 cells. A549 cells were infected with *B. thailandensis* expressing TssB-5-msfGFP (grey) at MOI 200. Host cells were pre-treated with 0.25 mg/mL of fluorescent dextran for 18 h (magenta). Yellow arrows indicate T6SS-5 assembly events. Scale bar represents 2 µm. Images are representative of at least three biological replicates. Source data are available online for this figure.

**Figure EV1:**
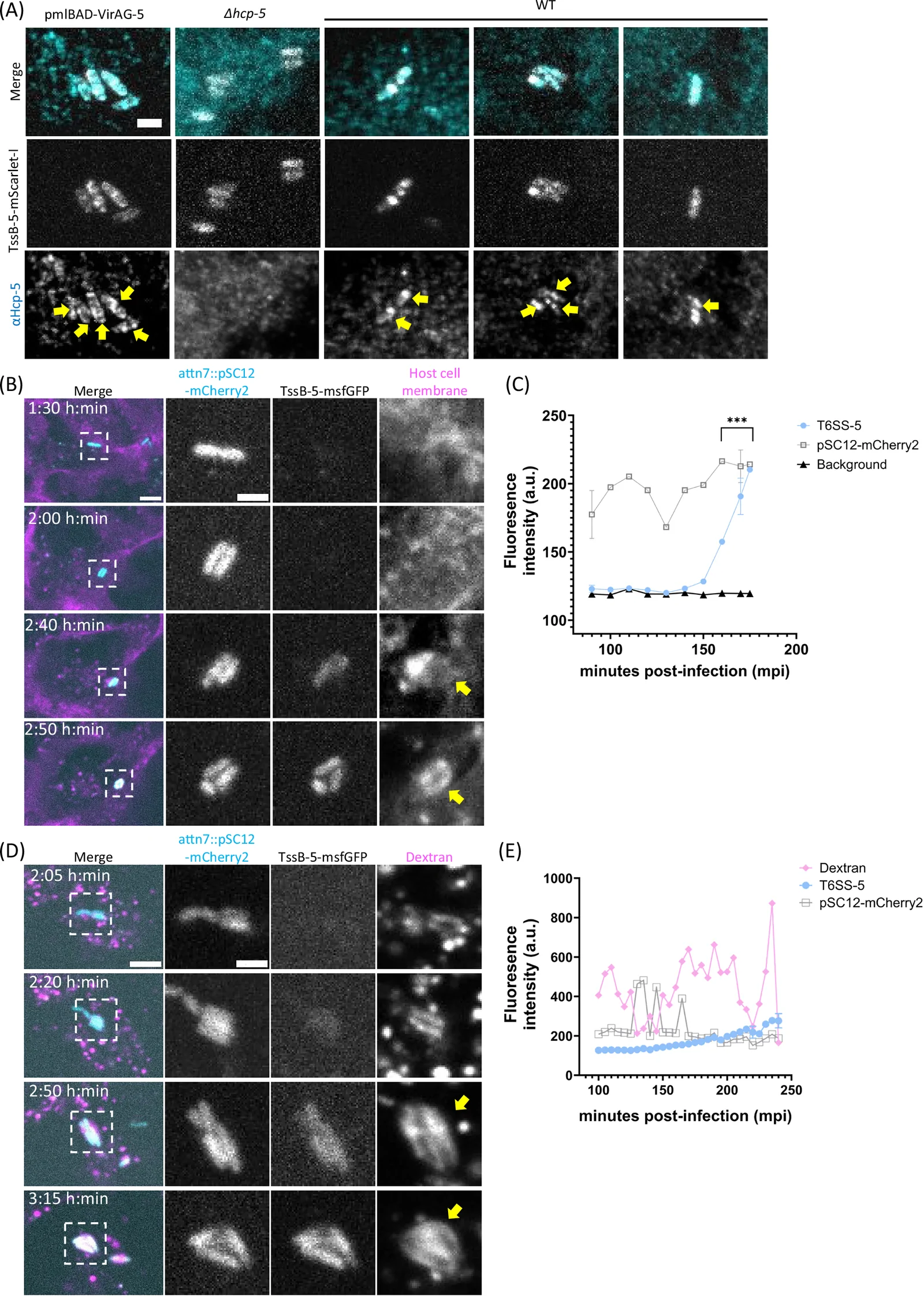
Expression of the anti-eukaryotic T6SS-5 structural and regulatory components in vitro and during infection. (**A**) Immunofluorescence staining of Hcp-5. A549 cells were infected with various *B. thailandensis* strains at MOI 500 and fixed with methanol at 3 hpi. The slide was then permeabilized with lysozyme and mutanolysin for 1 h at 37 degrees. Then stained with anti-hcp-5 antibody at 1 µg/mL for 1 h at room temperature. Then the slide was washed and stained with anti-rabbit-AlexaFluor488 or 647 overnight at 4 degrees. Scale bar represents 2 µm. Yellow arrows indicate Hcp-5 expression. Images representative of at least three biological replicates. (**B**) Time-lapse imaging of A549 cells infected with *B. thailandensis* expressing bacterial cytosolic mCherry2 under ribosomal promoter pSC12 (cyan) and TssB-5-msfGFP (grey) at MOI of 50. Host cell membrane was stained with CellMask deep red (magenta). Yellow arrows indicate host cell membrane around the bacteria. Scale bars represent 5 µm in the overview image and 5 µm in the magnified image. (**C**) Fluorescence intensity of various channels during the infection period shown in (**B**). Bacteria were segmented and tracked, and fluorescence intensities of TssB-5-msfGFP (T6SS-5, in blue), bacterial cytosol (pSC12-mCherry2, in grey), and background (black) were plotted. A two-way ANOVA with Tukey’s multiple comparisons test was performed (***P < 0.0001). Adjusted *P* values for the comparison between reporter and background signals at each time point were: 160 min, *P* = 4.34 × 10⁻⁸; 165 min, *P* = 1.4  × 10⁻¹⁴; 170 min, *P* < 1 × 10⁻¹⁵. Error bars represent mean ± SD. (**D**) Time-lapse imaging of A549 cells infected with *B. thailandensis* expressing bacterial cytosolic mCherry2 under ribosomal promoter pSC12 (cyan) and TssB-5-msfGFP (grey) at MOI of 200. Host cells were pre-treated with 0.25 mg/mL of fluorescent dextran for 18 h (magenta). Yellow arrows indicate bacteria expressing T6SS-5 when inside of a dextran-labelled vacuole. Scale bars represent 5 µm in the overview image and 5 µm in the magnified image. (**E**) Fluorescence intensity of various channels during the infection period shown in (**D**). Bacteria were segmented and tracked, and fluorescence intensities of TssB-5-msfGFP (T6SS-5, in blue), bacterial cytosol (pSC12-mCherry2, in grey), and fluorescent dextran (magenta) were plotted.

To investigate the T6SS-5 expression dynamics during infection, we infected a monolayer of A549 cells with *B. thailandensis tssB-5-mScarlet-I* constitutively expressing cytosolic mCherry2 under *Burkholderia* ribosomal promoter *pSC12* at a Tn7 insertion site. Host cell membranes were stained with CellMask deep red for 5 min and washed before imaging. Kanamycin was added to the medium to kill extracellular bacteria. Images were taken at 1-min intervals to identify the time point when TssB-5-msfGFP is expressed. During a 5-h imaging period, we tracked the bacteria and measured change in TssB-5-msfGFP fluorescence intensity in time. A region free of host cells and bacteria was selected at each timepoint to measure background green fluorescence. Using a two-way ANOVA with Tukey’s multiple comparisons test, we identified that T6SS-5 expression became significantly different from the background signal at approximately 3 h post-infection (Figs. 1B,C and EV1B,C; Movie EV1). Doing similar analysis on 116 bacteria across three biological replicates, we observed that most bacteria express T6SS-5 between 2 and 4 hpi (Fig. 1D).

Since previous studies focused on the expression of Hcp-5 but not TssB-5 (Chen et al, 2011; Wong et al, 2015), we investigated if Hcp-5 was also expressed within our identified time frame of TssB-5 expression. We performed immunofluorescence staining with an anti-Hcp-5 antibody on infected cells fixed at 3 hpi (Fig. EV1A). Hcp-5 was detected in a positive control, a *B. thailandensis* strain expressing VirAG from a pmlBAD plasmid, while no Hcp-5 expression was detected in the negative control ∆*hcp-5* strain and the wild-type *B. thailandensis* expressed both Hcp-5 and TssB-5.

**Figure EV2:**
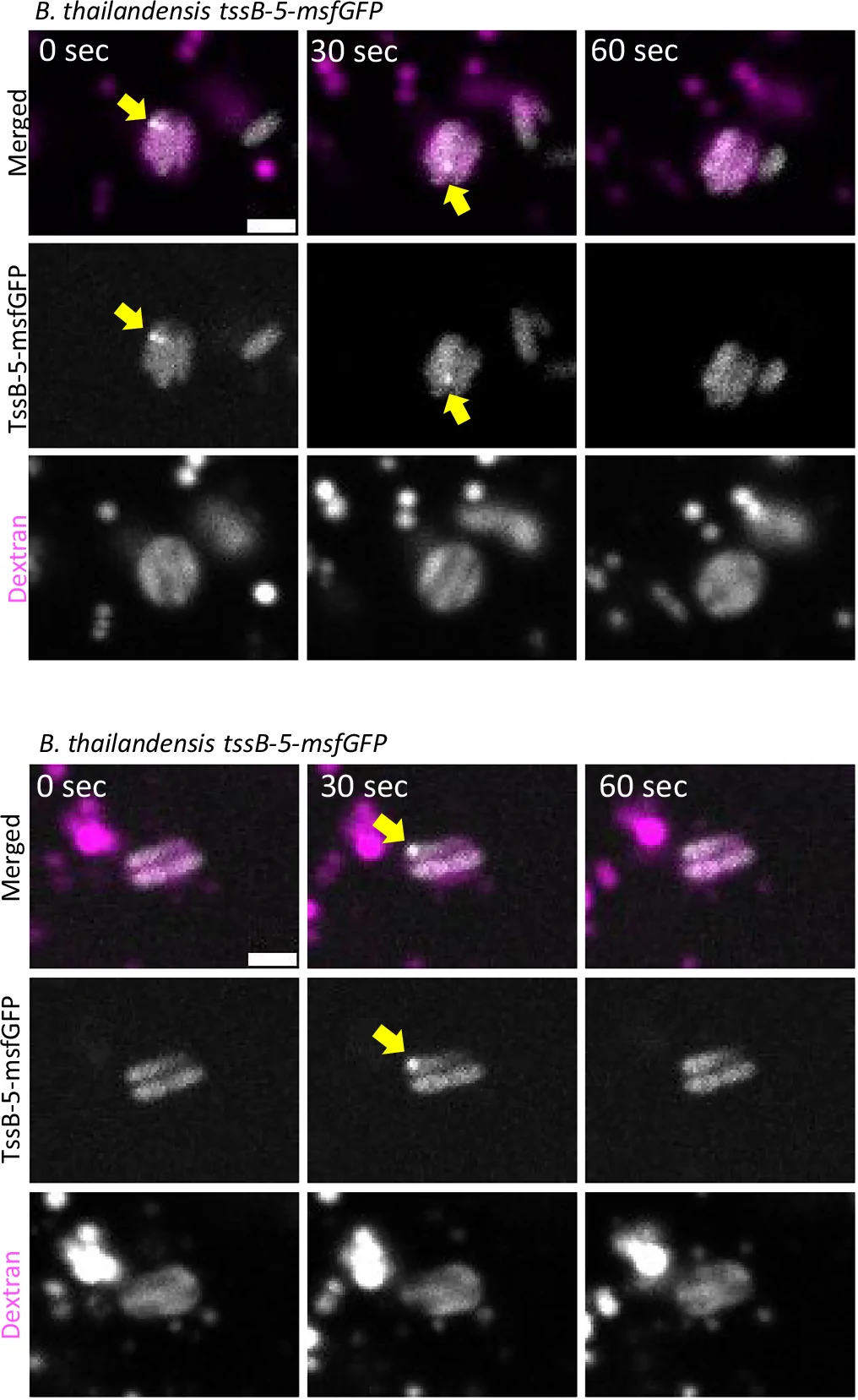
T6SS-5 assemblies in phagosome. Two examples of *B. thailandensis* assembling T6SS-5 (grey) at 3 hpi inside of a dextran-labelled vacuole (magenta) during infection of A549 cells. A549 cells were pre-treated with 0.25 mg/mL of fluorescent dextran to identify bacteria inside phagosomes. Yellow arrows indicate a T6SS-5 assembly event. Scale bar represents 2 µm. Images representative of at least three biological replicates.

Interestingly, we noticed that the bacteria were in a membrane-bound compartment when expressing T6SS-5 (Figs. 1B and EV1B, yellow arrows). We therefore asked if T6SS-5 is expressed while the phagosomal membrane is still intact. To test this, we pre-loaded A549 cells with 0.25 mg/mL fluorescent dextran 100 kDa overnight, infected the cells with *B. thailandensis* in the absence of dextran, washed with Optimem^TM^ with kanamycin (300 µg/mL) after 1 hpi and imaged immediately. We found that *B. thailandensis* expressed TssB-5-msfGFP when colocalized with dextran-labelled vacuoles (Figs. 1E and EV1D; Movie EV2). We then tracked the bacteria and measured the fluorescence intensity of TssB-5-msfGFP throughout the infection time course. Similar to Fig. 1C, fluorescence intensity of the dextran, T6SS-5 and bacterial cytosol (pSC12-mCherry2) were plotted (Figs. 1F and EV1E). The dextran signal remained between 500 and 2000 a.u. and only dropped to 200 a.u. after 2 h of increased T6SS-5 expression. By analyzing 76 bacteria in dextran-treated A549 cells across three biological replicates, we found that 87% of the bacteria expressed T6SS-5 when colocalized with fluorescent dextran. Furthermore, we imaged T6SS-5-expressing bacteria at 10 and 30 s intervals and found that *B. thailandensis* assembled T6SS-5 when inside of the phagosome (Figs. 1G and EV2; Movie EV3). Notably, the T6SS-5 assemblies did not disrupt the phagosomal membrane. This contrasts with the later stages of infection, when T6SS-5 assemblies are known to facilitate fusion of host cell membranes and cell-to-cell spread (Plum et al, 2024; Kostow and Welch, 2022).

### Phagosomal maturation is important for T6SS-5 expression

**Figure 2:**
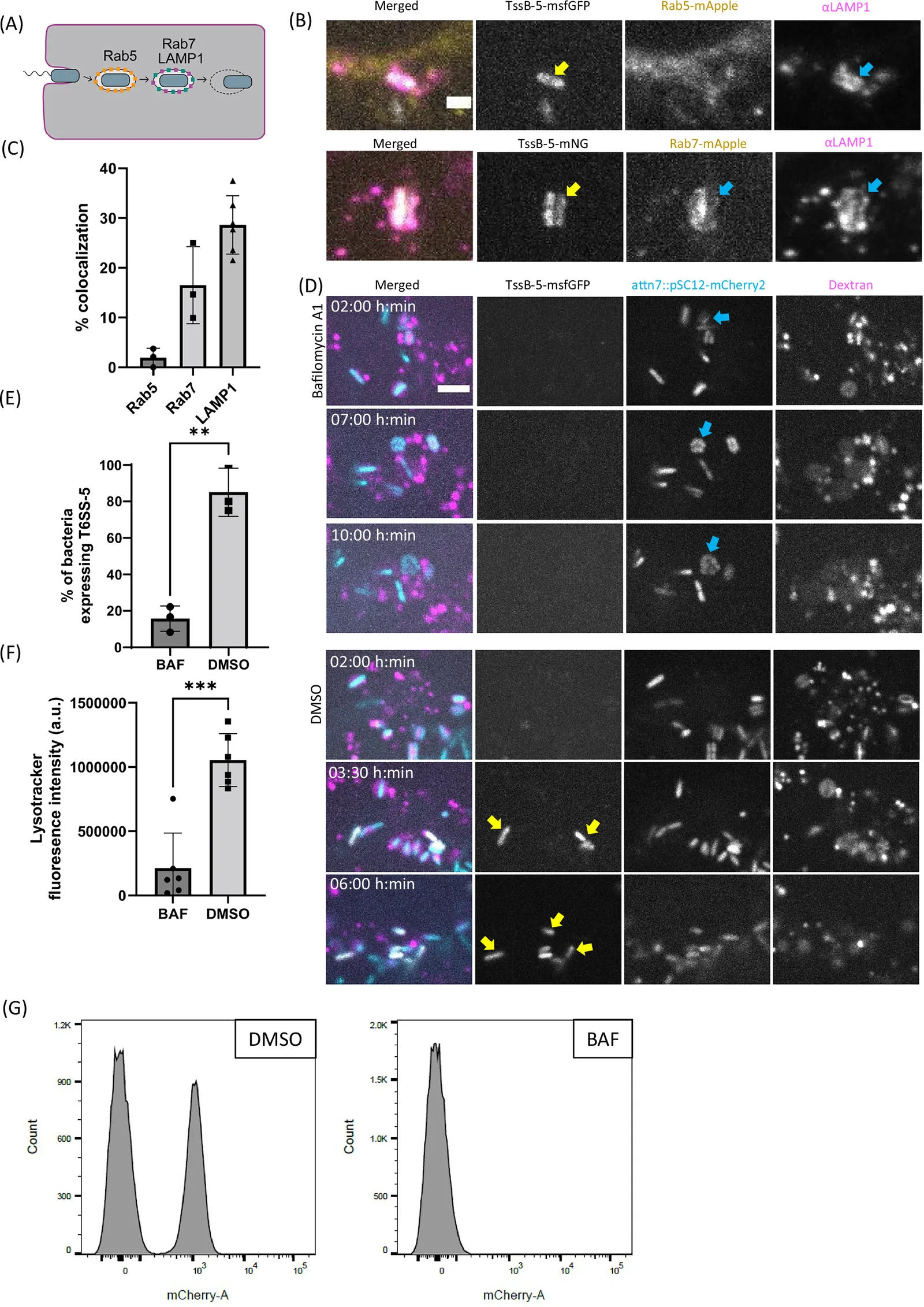
Phagosomal maturation is important for T6SS-5 expression. (**A**) Scheme depicting the phagosomal membrane markers when *B. thailandensis* enters a host cell. (**B**) LAMP-1 immunofluorescence staining of HeLa cells stably expressing Rab5 and Rab7-mApple infected with *B. thailandensis* at MOI 50. Cells were fixed and permeabilized with ice-cold methanol at 3 hpi and stained with anti-LAMP1 antibody at 1 µg/mL for 1 h at room temperature. Then the slide was washed with PBS and stained with anti-mouse-AlexaFluor647 overnight at 4 degrees. Yellow arrows indicate TssB-5 expressing bacteria. Blue arrows indicate the presence of phagosomal markers. Scale bar represents 2 µm. Images representative of three biological replicates. (**C**) Quantification of percentage of T6SS-5 expressing bacteria colocalizing with Rab5, Rab7, or LAMP-1 positive vacuoles at 3 hpi. Each data point represents one biological replicate (*n* = 3). More than 100 bacteria were counted in each biological replicate. Error bars represent mean ± SD. (**D**) Time-lapse imaging of A549 cells treated with bafilomycin A1 (100 nM) or DMSO infected with *B. thailandensis* expressing bacterial cytosolic mCherry2 under ribosomal promoter pSC12 (cyan) and TssB-5-msfGFP (grey) at MOI of 200. A549 cells were pre-treated with 0.25 mg/mL of fluorescent dextran for 18 h to identify intra-phagosomal bacteria. Host cells were pre-treated 1 h before infection, infected with *B. thailandensis* for 1 h without treatment, and bafilomycin A1 or DMSO was added and kept in the media with 300 µg/mL kanamycin from 1 hpi onwards. Host cell membrane was stained with CellMask deep red (magenta). Blue arrows indicate bacteria that do not express T6SS-1 and yellow arrows indicate T6SS-5 expressing bacteria. Scale bar represents 5 µm. Images representative of three biological replicates. (**E**) Percentage of intracellular *B. thailandensis* expressing T6SS-5 when A549 cells were treated with bafilomycin A1 or DMSO. A549 cells were pre-treated with 0.25 mg/mL of fluorescent dextran for 18 h to identify intra-phagosomal bacteria. A total of 109 bacteria were identified and tracked for T6SS-5 expression across three biological replicates. An unpaired *t* test were carried out (*n* = 3, **P < 0.01, *P* = 0.00387). Error bars represent mean ± SD. (**F**) Fluorescence intensity of A549 cells treated with either 100 nM bafilomycin A1 or DMSO stained with Lysotracker deep red. Fluorescent images were adjusted with the same threshold values and integrated density values were measured with ImageJ. An unpaired *t* test were carried out (*n* = 2, ***P < 0.001, *P* = 0.000174). Error bars represent mean ± SD. (**G**) Histogram plots showing the number of bacteria expressing TssB-5-mScarlet-I at 15 hpi in A549 cells treated with DMSO or bafilomycin A1 (BAF). Data are from three biological replicates (*n* = 3), two additional independent biological replicates are shown in Fig. EV3C. Source data are available online for this figure.

To assess phagosomal maturation during infection with *B. thailandensis* expressing T6SS-5, we infected two HeLa cell lines stably expressing Rab5-mApple or Rab7-mApple and stained for LAMP1 (lysosome-associated membrane protein-1) by immunofluorescence. Rab5 is a GTPase associated with early phagosomes, Rab7 functions mainly on late phagosomes, and LAMP1 is a marker for acidified vacuoles (Kitano et al, 2008; Vieira et al, 2003; Hu et al, 2019; Chen et al, 1985a; Huynh et al, 2007; Chen et al, 1985b; Zhang et al, 2023). Figure 2A shows a schematic of the markers associated with the phagosomal pathway. We found that most bacteria express T6SS-5 in late phagosomal maturation stages, indicated by colocalization with Rab7-mApple, and that T6SS-5 expressing bacteria often colocalize with LAMP1-positive vacuoles between 2 and 3 hpi (Fig. 2B). We quantified over 400 T6SS-5 expressing bacteria in at least 3 biological replicates, and tested for colocalization with either Rab5, Rab7, and LAMP1. Only 2% of T6SS-5 expressing bacteria colocalize with Rab5-labelled phagosomes; whereas 16.5% of those colocalize with Rab7-labelled phagosomes and 29% with LAMP1-labelled phagosomes (Fig. 2C).

Since LAMP1 is a common marker for mature, acidified vacuoles (Chen et al, 1985a; Zhang et al, 2023; Huynh et al, 2007), we explored the role of phagosomal maturation as a signal for T6SS-5 expression. Acidification of vacuoles can be inhibited by bafilomycin A1 (Yamamoto et al, 1998; Matsumoto and Nakanishi-Matsui, 2019; Mauvezin and Neufeld, 2015). We pre-treated A549 cells with 100 nM bafilomycin A1 (BAF) 1 h before infection, infected the cells with *B. thailandensis*, washed with kanamycin at 1 hpi, and replenished the media with BAF. At the same time, we also pre-loaded the host cells with 0.25 mg/mL fluorescent dextran 100 kDa overnight to label phagosomes. To ensure BAF inhibits the acidification of vacuoles in our assays, we measured the fluorescence intensity of lysotracker and showed that BAF treatment significantly reduced acidification of vacuoles (Fig. 2F). Importantly, the addition of BAF largely abolished T6SS-5 expression (Fig. 2D; Movie EV4). We quantified the percentage of bacteria expressing T6SS-5 with or without BAF treatment (Fig. 2E). We analysed 69 bacteria in host cells treated with BAF, and 41 bacteria in host cells treated with DMSO across three biological replicates. Only 8 bacteria (11.5%) expressed T6SS-5 when the host cells were treated with BAF, compared to 85% of bacteria expressing T6SS-5 in the DMSO control. Among the eight T6SS-5-expressing bacteria residing in BAF-treated cells, one bacterial cluster escaped from the phagosome, whereas the rest of the bacteria remained confined within the phagosome (Appendix Fig. S2A,B).

**Figure EV3:**
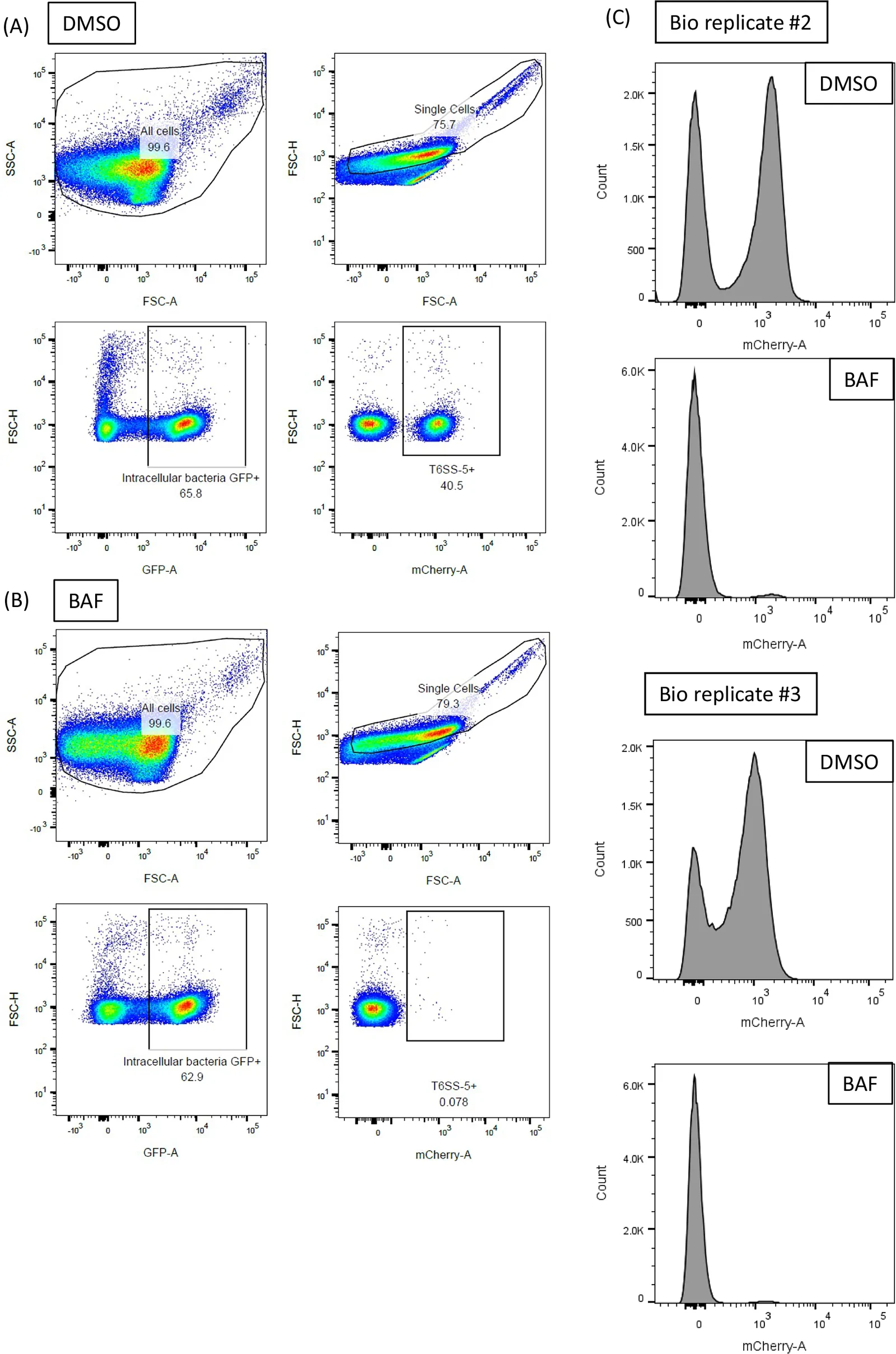
Flow cytometry gating strategy and biological replicates. Representative gating strategy for bacteria in (**A**) DMSO-treated, or (**B**) bafilomycin A1 (BAF) treated A549 cells. Cells were sequentially gated on singlets and mNeonGreen-positive bacteria. Bacteria constitutively expressing bacterial cytosolic mNeonGreen from a ribosomal promoter (Tn7 insertion) were identified, and the proportion of TssB-5-mScarlet-I-positive bacteria was quantified using the mCherry channel during flow cytometry. (**C**) Histograms showing the proportion of TssB-5-mScarlet-I positive bacteria in DMSO-treated or BAF-treated cells from two independent biological replicates. In total, 100,000 events per conditions were recorded.

To obtain a larger sample size, we performed flow cytometry on infected cells treated with BAF or DMSO. In line with our fluorescence imaging data, flow cytometry confirmed the lack of TssB-5-mScarlet-I expression in BAF-treated cells, which is indicated by the absence of mCherry signal (Figs. 2G and EV3C). This indicates that phagosomal maturation and acidification is an important signal for T6SS-5 expression.

### Bacteria remain confined within dextran-labelled phagosomes in BAF-treated cells

**Figure 3:**
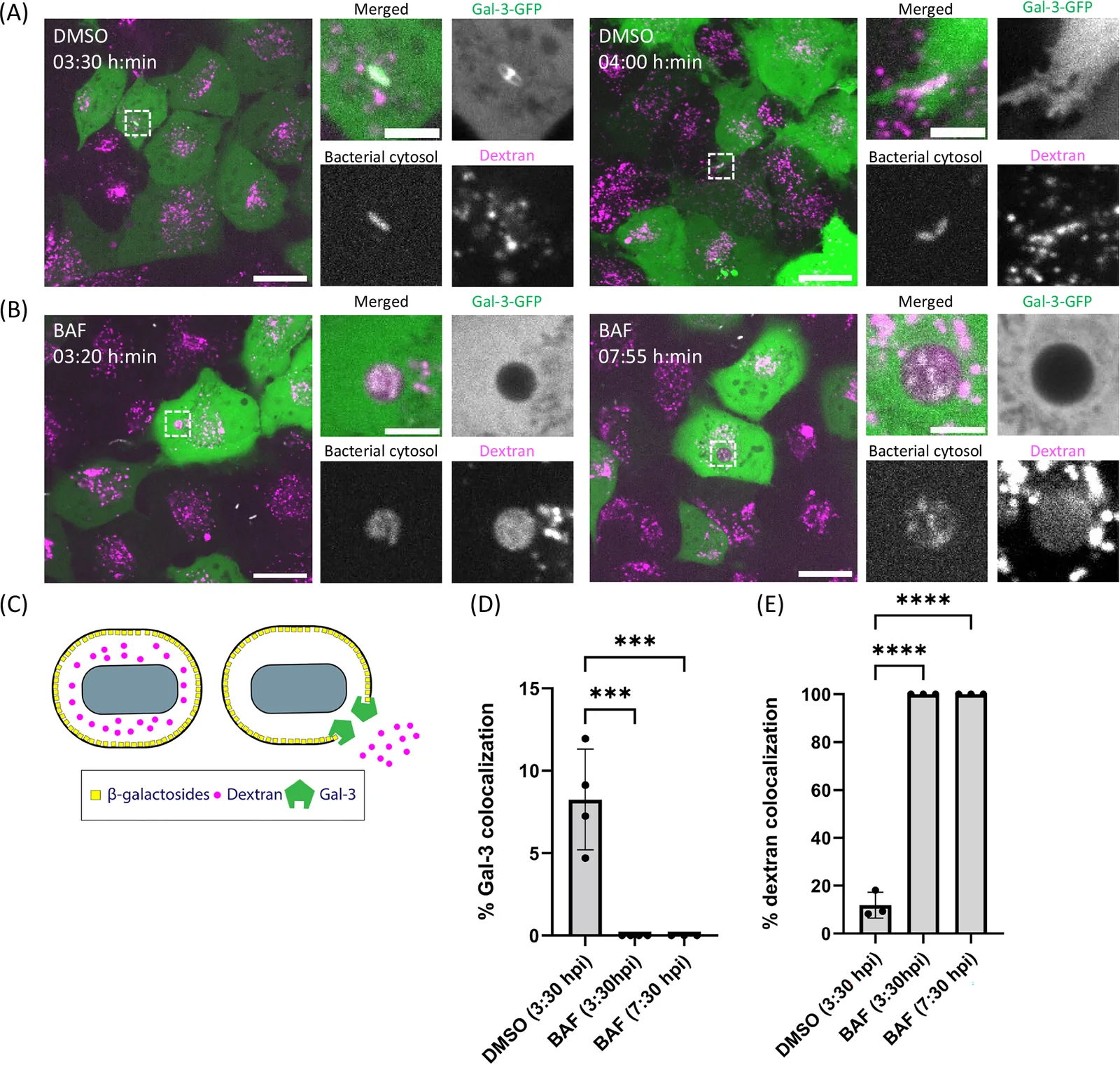
Lack of Galectin-3 colocalization to phagosomes in bafilomycin-treated cells. (**A**) HeLa cells stably expressing galectin-3-GFP (green) were treated with DMSO and infected with *B. thailandensis* expressing cytosolic TssB-5-mScarlet-I (grey) at MOI 200. Host cells were pre-treated with 0.25 mg/mL of fluorescent dextran for 18 h (magenta). Single time-point images were acquired between 3 and 4 hpi using 5 z-slices with 0.5 µm step sizes and displayed as maximum intensity projections. Representative examples of galectin-3 colocalization with bacteria (left) and absence of galectin-3 (right) are shown. Scale bars represent 20 µm in overview images and 5 µm in magnified images. Images representative of at least three biological replicates. (**B**) HeLa cells stably expressing galectin-3-GFP (green) were treated with 100 nM bafilomycin A1 (BAF) and infected with *B. thailandensis* expressing cytosolic mCherry2 under ribosomal promoter (grey) at MOI 200. Host cells were pre-treated with 0.25 mg/mL of fluorescent dextran for 18 h (magenta). Single time-point images were acquired between 3 and 4 hpi and 7–8 hpi using 5 z-slices with 0.5 µm step sizes and displayed as maximum intensity projections. Representative examples of the absence of galectin-3 at early and late infection timepoints are shown. Scale bars represent 20 µm in overview images and 5 µm in magnified images. Images representative of at least three biological replicates. (**C**) A scheme showing dextran retainment in phagosomes with intact membrane and galectin-3 recruitment to β-galactosides exposed upon phagosomal membrane rupture. (**D**, **E**) Quantification of (**D**) galectin-3 colocalization and (**E**) dextran localization to bacteria. HeLa cells were treated and infected as mentioned above. Multiple bacteria residing within the same phagosome were scored as a single event, whereas cytosolic bacteria were quantified individually. At least 50 events were counted per condition per biological replicates. A one-way ANOVA with Tukey’s multiple comparisons test was performed (***P < 0.001, ****P < 0.0001). Adjusted *P* values were as follows: (**D**) [group DMSO (3:30 hpi) vs. BAF (3:30 hpi)], *P* = 4.62 × 10⁻⁴; [group DMSO (3:30 hpi) vs. BAF (7:30 hpi)], *P* = 7.71 × 10⁻⁴. (**E**) [group DMSO (3:30 hpi) vs. BAF (3:30 hpi)], *P* = 7.41 × 10⁻⁸; [group DMSO (3:30 hpi) vs. BAF (7:30 hpi)], *P* = 7.41 × 10⁻⁸. Error bars represent mean ± SD (*n* = 3). Source data are available online for this figure.

In our time-lapse imaging experiments, we observed that bacteria replicated while remaining confined within dextran-labelled vacuoles at 10 hpi (Fig. 2D). To detect phagosomal rupture, we examined galectin-3 (Gal-3) colocalization to bacteria-containing compartments since Gal-3 binds to β-galactosides exposed upon membrane rupture (Jia et al, 2020; Paz et al, 2010; Hughes, 1999; Fig. 3C). We infected HeLa cells stably expressing cytosolic Gal-3-GFP, preincubated the cells with fluorescently labelled dextran as described above, and either treated the cells with BAF or DMSO. Images were acquired at two time points: 3–4 hpi (to assess phagosomal escape) and 7–8 hpi (to assess replication within phagosomes).

Representative images of *B. thailandensis* with or without Gal-3 colocalization in DMSO-treated cells are presented in Fig. 3A. In contrast, no Gal-3 colocalization was observed for bacteria residing within dextran-labelled phagosomes in BAF-treated cells at approximately 3 hpi or 8 hpi (Fig. 3B). We further quantified the proportion of bacteria colocalizing with Gal-3 in cells treated with BAF or DMSO. At 3:30 hpi, amongst 776 bacteria, 60 bacteria showed Gal-3 colocalization (8%) of bacteria colocalized with Gal-3 in DMSO-treated cells, whereas none of the 697 bacteria-containing vacuoles colocalized with Gal-3 at both 3:30 and 7:30 hpi in BAF-treated cells (Fig. 3D). In parallel, we quantified bacterial localization within dextran-labelled vacuoles and found that 100% of bacteria remained within dextran-labelled vacuoles at both 3:30 and 7:30 hpi, compared 12% in DMSO-treated cells at 3:30 hpi (Fig. 3E).

These observations indicate that dextran labelling of phagosomes was not affected by BAF treatment and suggest that bacteria remained confined within intact phagosomes throughout infection, consistent with the absence of phagosomal membrane damage and the retention of dextran within the vacuolar compartment.

### In the host cell cytosol, T6SS-1 is expressed but not assembled

**Figure 4:**
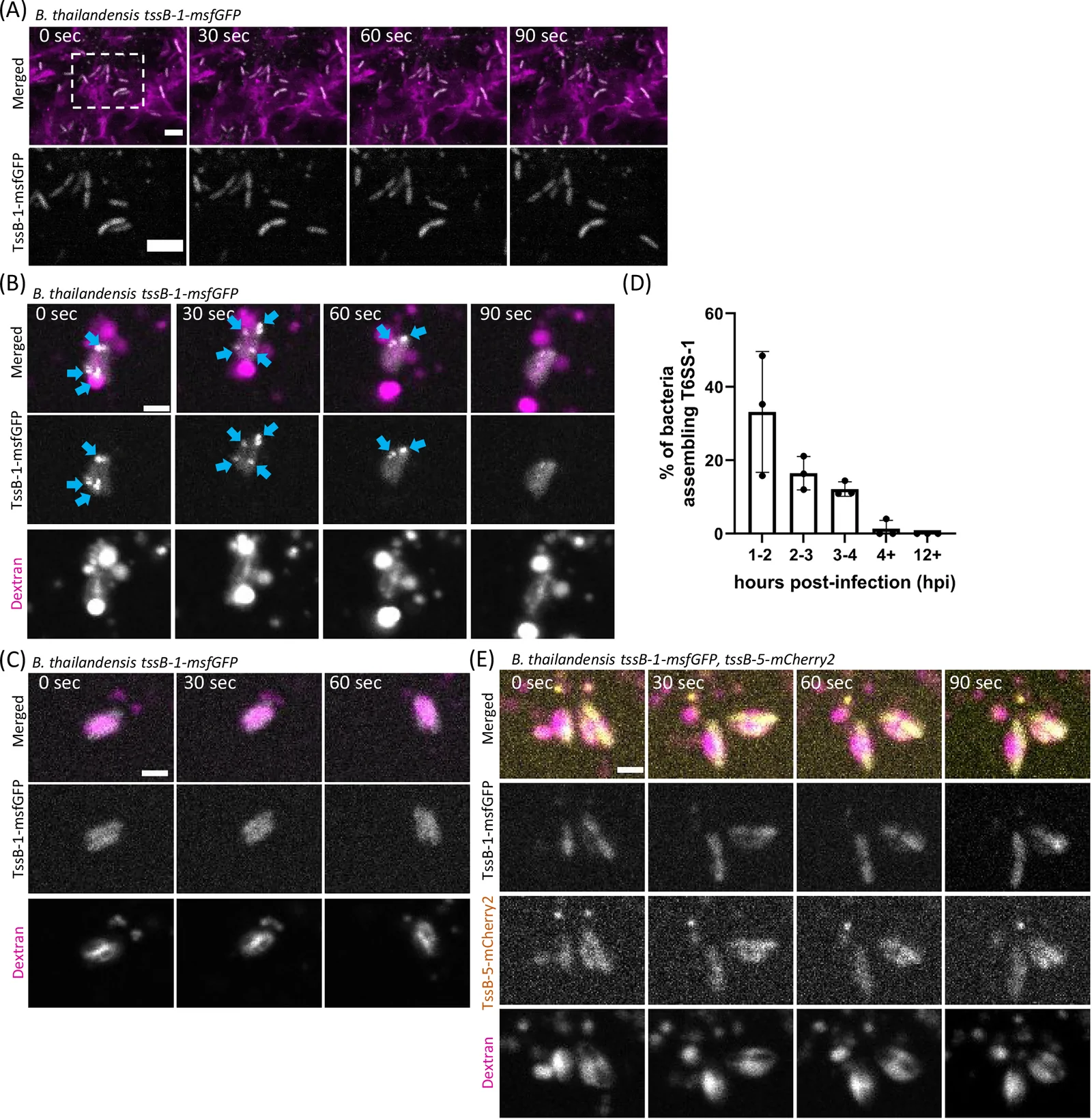
Expression and assembly dynamics of T6SS-1 during infection. (**A**) Time-lapse microscopy of A549 cells infected with *B. thailandensis* TssB-1-msfGFP at MOI of 50 at 13 hpi. Host cell membranes were stained with CellMask deep red (magenta). Scale bars represent 5 µm. Images representative of at least 3 biological replicates. (**B**) Example of *B. thailandensis* assembling T6SS-1 inside of a dextran-labelled phagosome in A549 cells. Host cells were pre-treated with 0.25 mg/mL of fluorescent dextran for 18 h (magenta). Images were taken at 45 min post-infection. Blue arrows indicate T6SS-1 assembly events. Scale bar represents 2 µm. Images representative of at least three biological replicates. (**C**) Example of *B. thailandensis* not assembling T6SS-1 inside of a dextran-labelled phagosome in A549 cells. Host cells were pre-treated with 0.25 mg/mL of fluorescent dextran for 18 h (magenta). Images were taken at 2 hpi. Scale bar represents 2 µm. Images representative of at least three biological replicates. (**D**) Quantification of the percentage of T6SS-1 assembling bacteria during infection of A549 cells at different time points. A549 cells were infected at an MOI of 200, and at least 100 intracellular bacteria were analyzed per biological replicate (*n* = 3). Each bacterium was imaged only once in a unique field of view, with time-lapse images acquired every 30 s over a 5-min period. Error bars represent mean ± SD. (**E**) Time-lapse microscopy of A549 cells infected with *B. thailandensis* expressing TssB-1-msfGFP and TssB-5-mCherry2 at MOI of 500 at 3 hpi. Host cells were pre-treated with 0.25 mg/mL of fluorescent dextran for 18 h (magenta). Scale bar represents 2 µm. Images representative of at least three biological replicates. Source data are available online for this figure.

**Figure EV4:**
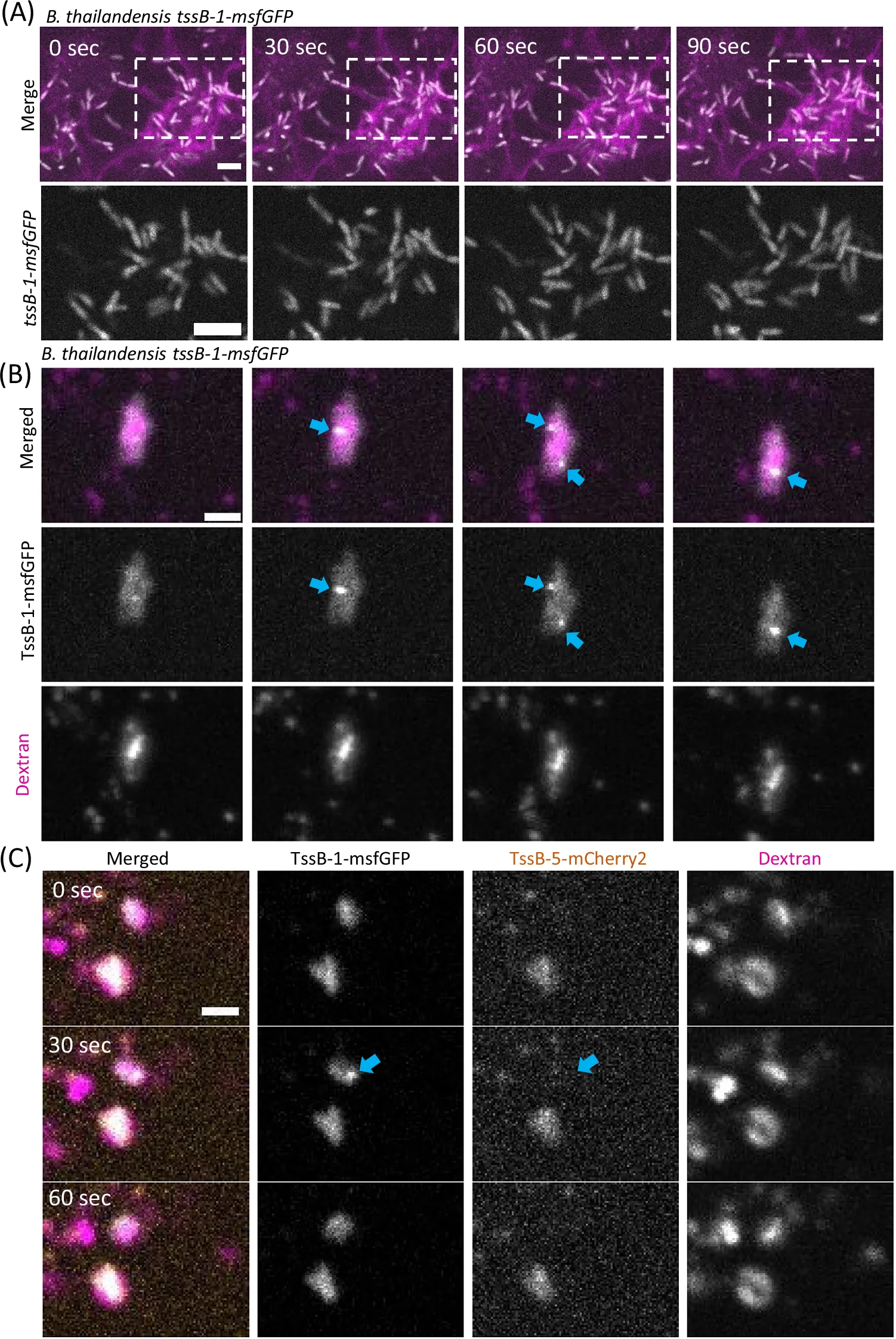
T6SS-1 assembly dynamics during infection. (**A**) Time-lapse microscopy of A549 cells infected with *B. thailandensis* TssB-1-msfGFP at MOI of 50 at 13 hpi. Host cell membranes were stained with CellMask deep red (magenta). Scale bars represent 5 µm. Images representative of at least three biological replicates. (**B**) Example of *B. thailandensis* assembling T6SS-1 inside of a dextran-labelled phagosome in A549 cells. Host cells were pre-treated with 0.25 mg/mL of fluorescent dextran for 18 h (magenta). Images were taken at 1:30 hpi. Blue arrows indicate T6SS-1 assembly events. Scale bar represents 2 µm. Images representative of at least 3 biological replicates. (**C**) Time-lapse microscopy of A549 cells infected with *B. thailandensis* expressing TssB-1-msfGFP and TssB-5-mCherry2 at MOI of 500 at 3 hpi. Host cells were pre-treated with 0.25 mg/mL of fluorescent dextran for 18 h (magenta). Blue arrows indicate a bacteria assembling T6SS-1 and not expressing T6SS-5. Scale bar represents 2 µm. Images representative of three biological replicates.

It was shown previously that *B. thailandensis* constitutively express and assemble T6SS-1 when imaged on agar pads (Lin et al, 2022). Little is known about how they regulate the T6SS-1 during infection. To test this, we infected A549 cells with a *B. thailandensis* strain that expressed the T6SS-1 sheath protein fused to a fluorophore, TssB-1-msfGFP, and performed live-cell imaging after 12 hpi. To our surprise, we observed that the T6SS-1 was expressed but not assembled, as indicated by uniform fluorescence throughout the bacterial cells without the appearance of dynamic foci typically associated with T6SS assembly events (Figs. 4A and EV4A; Movie EV5). To identify the point of T6SS-1 assembly inhibition, we infected A549 cells and acquired time-lapse images of at least 100 different bacteria in unique field of views across different time points. Images were captured every 30 s over a 5-min period to assess whether the bacteria assembled T6SS-1. At early infection time points when the *B. thailandensis* were in dextran-labelled vacuoles, all bacteria expressed T6SS-1, but not all bacteria assembled T6SS-1 (Figs. 4B,C and EV4B; Movie EV6). We quantified the percentage of bacteria assembling T6SS-1 at various time points, and found that 33% of intracellular bacteria assembled T6SS-1 between 1 and 2 hpi. This significantly decreased to 16% when imaged between 2 and 3 hpi. 11% of bacteria assembled T6SS-1 between 3 and 4 hpi and this further decreased to 1% after 4 hpi and 0% after 12 hpi (Fig. 4D). In all the time points, none of the cytosolic bacteria assembled T6SS-1. When we infected A549 cells with a *B. thailandensis* strain with both T6SS-1 and T6SS-5 fluorescently labelled, we found that amongst 93 bacteria, when the T6SS-5 was expressed, there were no T6SS-1 assemblies (Figs. 4E and EV4C). This data suggests that expression of T6SS-5 is negatively correlated with the assembly of T6SS-1.

### VirAG inhibits T6SS-1 assemblies during infection

**Figure 5:**
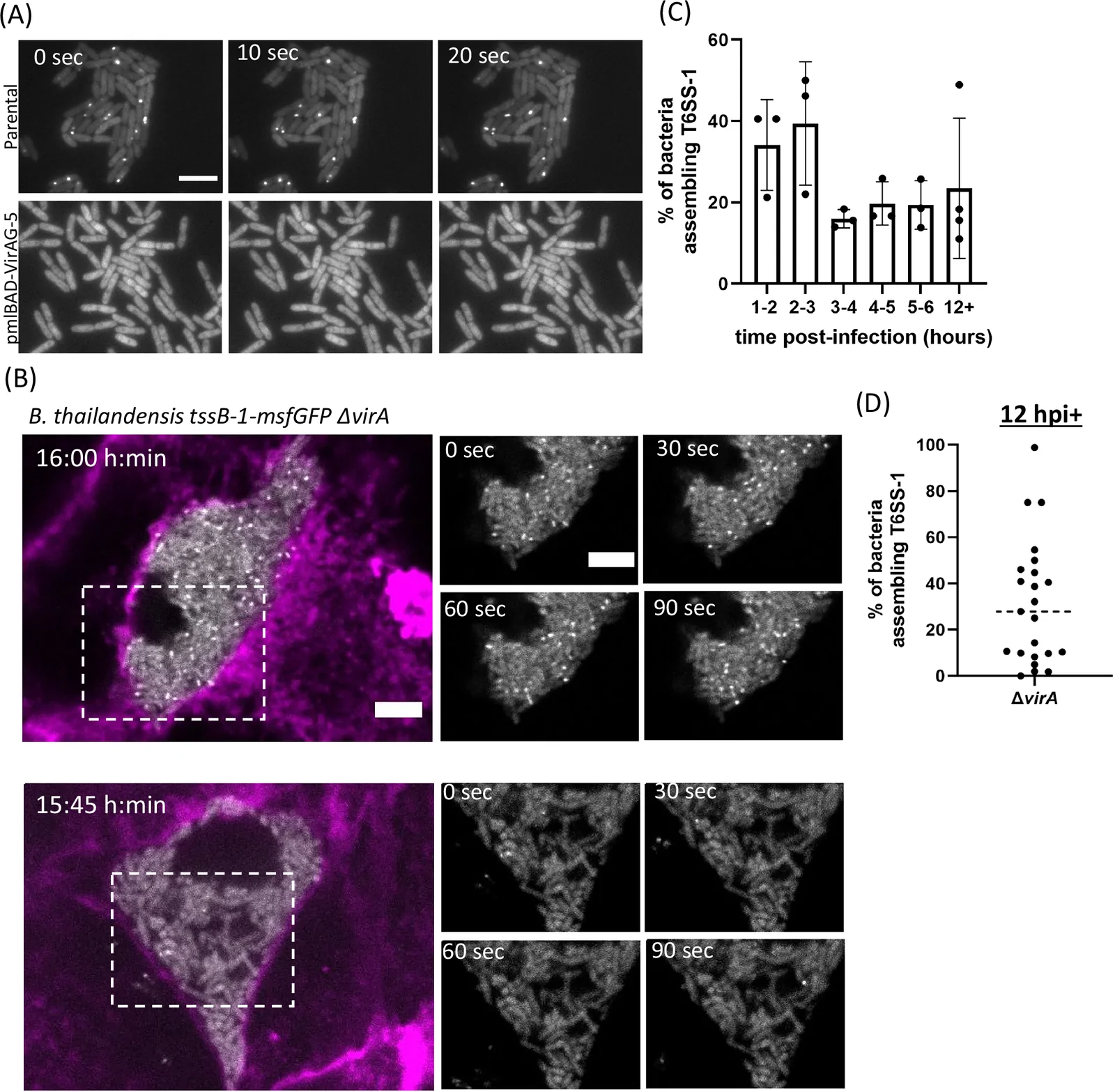
Inhibition of T6SS-1 assemblies by VirAG in vitro and during infection. (**A**) Time-lapse microscopy of *B. thailandensis* TssB-1-msfGFP parental strain (top) and a stain harbouring the arabinose-inducible plasmid pmlBAD-VirAG (bottom) on agar pads. Overnight bacterial cultures were subcultured to mid-log phase with 0.4% L-arabinose in LB and imaged on LB agar pads. Scale bar represents 5 µm. Images representative of at least three biological replicates. (**B**) Two examples of A549 cells infected with *B. thailandensis* ∆*virA* expressingTssB-1-msfGFP at MOI of 50 at 16 and 15:45 hpi. Host cell membranes were stained with CellMask deep red (magenta). Scale bars represent 5 µm. Images representative of at least three biological replicates. (**C**) Quantification of the percentage of *B. thailandensis* ∆*virA* bacteria that assembled T6SS-1 at different infection time points. A549 cells were infected at an MOI of 200, and at least 100 intracellular bacteria were analyzed per biological replicate (*n* = 3). Each bacterium was imaged only once in a unique field of view, with time-lapse images acquired every 30 s over a 5-min period. Error bars represent mean ± SD. (**D**) Scatter plot showing the percentage of *B. thailandensis* ∆*virA* assembling T6SS-1 per host cell. A549 cells were infected at MOI 50 and between 12 and 16 hpi, time-lapse images were acquired every 30 s over a 5-min period. Each data point represents a single host cell, where the total number of bacteria and the number of bacteria assembling T6SS-1 were counted. Data pooled from three biological replicates. Source data are available online for this figure.

Since VirAG is a two-component system that upregulates the T6SS-5 expression at the beginning of infection, we hypothesized that VirAG also plays a role in inhibiting T6SS-1 assembly. We expressed VirAG from an arabinose-inducible plasmid and imaged T6SS-1 assembly dynamics on agar pads in vitro (Fig. 5A; Movie EV7). The parental *B. thailandensis* strain expressing TssB-1-msfGFP assembled T6SS-1 constitutively. However, when VirAG was expressed, T6SS-1 assembly was completely abolished.

**Figure EV5:**
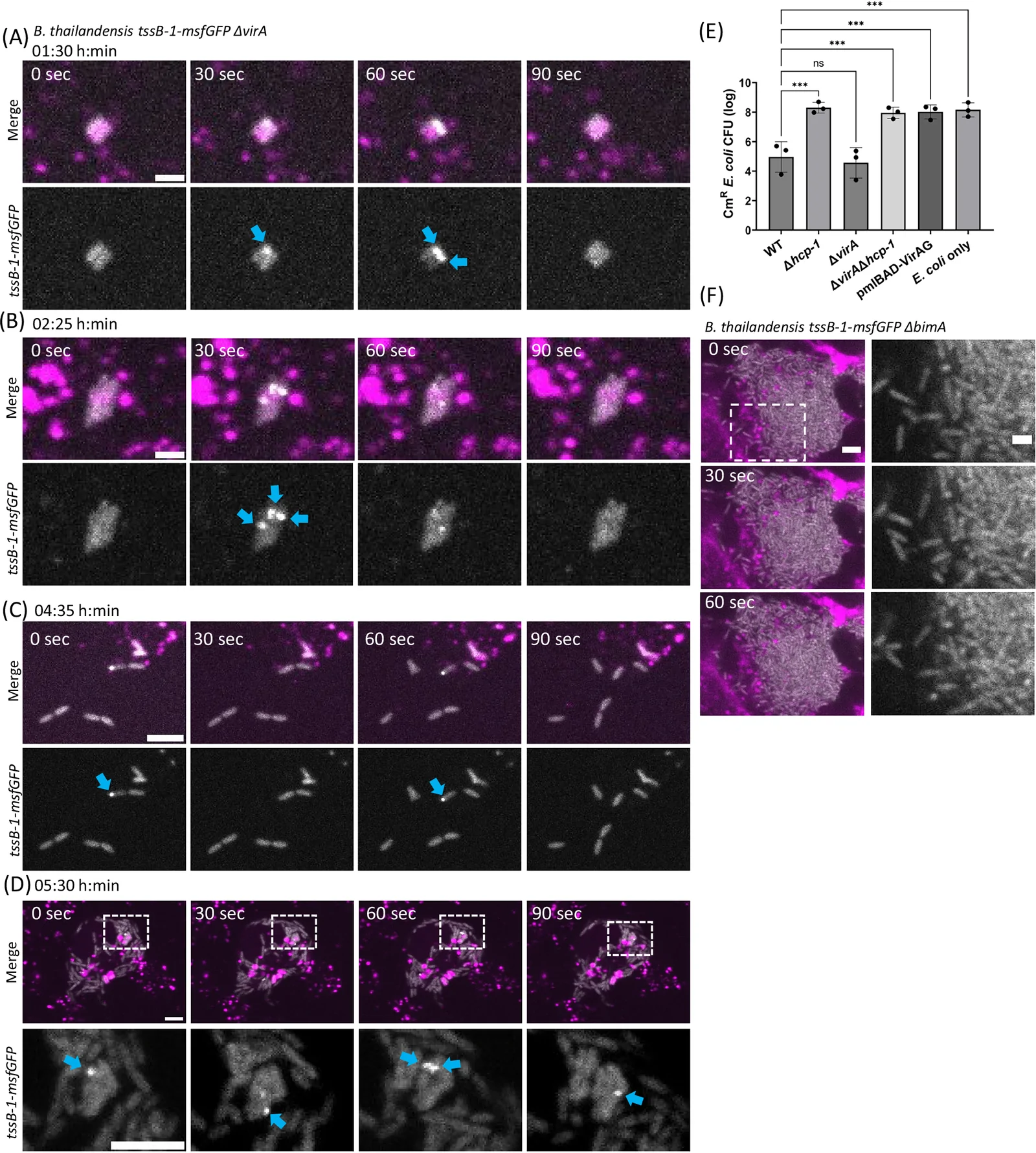
Intracellular T6SS-1 dynamics and bacterial competition assay. Time-lapse microscopy of A549 cells infected with *B. thailandensis ∆virA*, TssB-1-msfGFP at MOI of 50 at (**A**) 1:30 hpi, (**B**) 2:25 hpi, (**C**) 4:35 hpi, and (**D**) 5:30 hpi. A549 cells were pre-treated with 0.25 mg/mL of fluorescent dextran (magenta) to identify bacteria inside phagosomes. Blue arrows indicate T6SS-1 assembly events. Scale bars represent 2 µm for (**A**, **B**) and 5 µm for (**C**, **D**). Images representative of at least three biological replicates. (**E**) Recovery of viable *E. coli* prey after competition with *B. thailandensis* strains. *E. coli* prey harbouring a plasmid conferring chloramphenicol resistance was co-cultured with *B. thailandensis* strains at 1:1 ratio on LB agar plates for 5 h at 30 degrees. The co-cultures were harvested and serially diluted and spotted on LB agar plates containing 30 µg/mL chloramphenicol. Colony forming units (CFU) of *E. coli* was plotted on a logarithmic scale. Ordinary one-way ANOVA with Dunnett’s multiple comparisons test was performed (***P < 0.001, ns = not significant). Adjusted *P* values were as follows: [group WT vs. Δ*hcp-1*], *P* = 3.0 × 10⁻⁴; [group WT vs. Δ*virA*], *P* = 9.260 × 10⁻¹; [group WT vs. Δ*virA*Δ*hcp-1*], *P* = 8.0 × 10⁻⁴; [group WT vs. pmlBAD-VirAG], *P* = 7.0 × 10⁻⁴; [group WT vs. *E. coli*], *P* = 5.0 × 10⁻⁴. Error bars represent mean ± SD. (**F**) Time-lapse microscopy of A549 cells infected with *B. thailandensis* ∆*bimA*, TssB-1-msfGFP at MOI of 50 at 14 hpi. Host cell membranes were stained with CellMask deep red (magenta). Scale bars represent 5 µm. Images representative of three biological replicates.

To test this during infection, we constructed a *virA* knock-out strain. To ensure that the T6SS-1 assemblies of the Δ*virA* mutant are functional, we performed a bacterial competition assay with *E. coli* as prey (Fig. EV5E). Whereas the T6SS-1 deletion strain (Δ*hcp-1*) showed no killing, the WT and Δ*virA* mutant showed similar killing efficiency of *E. coli*, thus confirming that the T6SS-1 assemblies of the Δ*virA* mutant are bactericidal.

We then infected A549 cells with the Δ*virA* mutant and imaged at 16 hpi. Deletion of *virA* allowed *B. thailandensis* to assemble T6SS-1 during infection (Fig. 5B; Movie EV8). The Δ*virA* bacteria accumulated inside of single host cells and failed to spread. This is presumably because the protein required for actin tail motility, BimA, was reported to be not expressed in the absence of VirAG (Chen et al, 2011). We then explored if *B. thailandensis* Δ*virA* assembles T6SS-1 at different time points during infection. We infected A549 cells and acquired time-lapse images of 1834 different bacteria in unique field of views across different time points in three biological replicates. Images were captured every 30 s over a 5-min period to assess whether the bacteria assembled T6SS-1. We found that bacteria assembled T6SS-1 both when inside of dextran-labelled phagosomes or in the cytoplasm (Fig. EV5A–D). Throughout the imaging period, 15–40% of *B. thailandensis* Δ*virA* assembled T6SS-1 from 1 hpi to 6 hpi (Fig. 5C). Interestingly, we noticed a high variability in the percentage of bacteria assembling T6SS-1 at late infection time points (Fig. 5D). We imaged 25 infected cells after 12 hpi, with 1,406 bacteria over three biological replicates, and found that in each infected cell, the percentage of bacteria assembling T6SS-1 varied from 1 to 98%.

In addition, since the Δ*virA* mutant lacks actin-tail motility and accumulated in a host cell at high bacterial cell density, we asked if the T6SS-1 assemblies observed in Fig. 4B could be caused by increased bacterial cell-cell contact rather than deletion of *virA*. To prevent bacterial spreading and induce accumulation in host cytoplasm, we infected A549 cells with a *virA* positive, *bimA* knock-out strain expressing TssB-1-msfGFP (Fig. EV5F). Importantly, in the presence of VirA, even when the bacteria were accumulated inside of one host cell and were in contact with one another, no T6SS-1 assemblies were observed suggesting that mere bacterial cell-cell contact or high number of bacteria in the cytoplasm are insufficient to trigger T6SS-1 assembly.

These results identify VirAG as an inhibitor of T6SS-1 activity during infection, as plasmid expression of VirAG suppresses T6SS-1 firing in vitro, whereas deletion of *virA* restores T6SS-1 assemblies during infection.

### Deletion of T6SS-5 components partially restore T6SS-1 assembly

**Figure 6:**
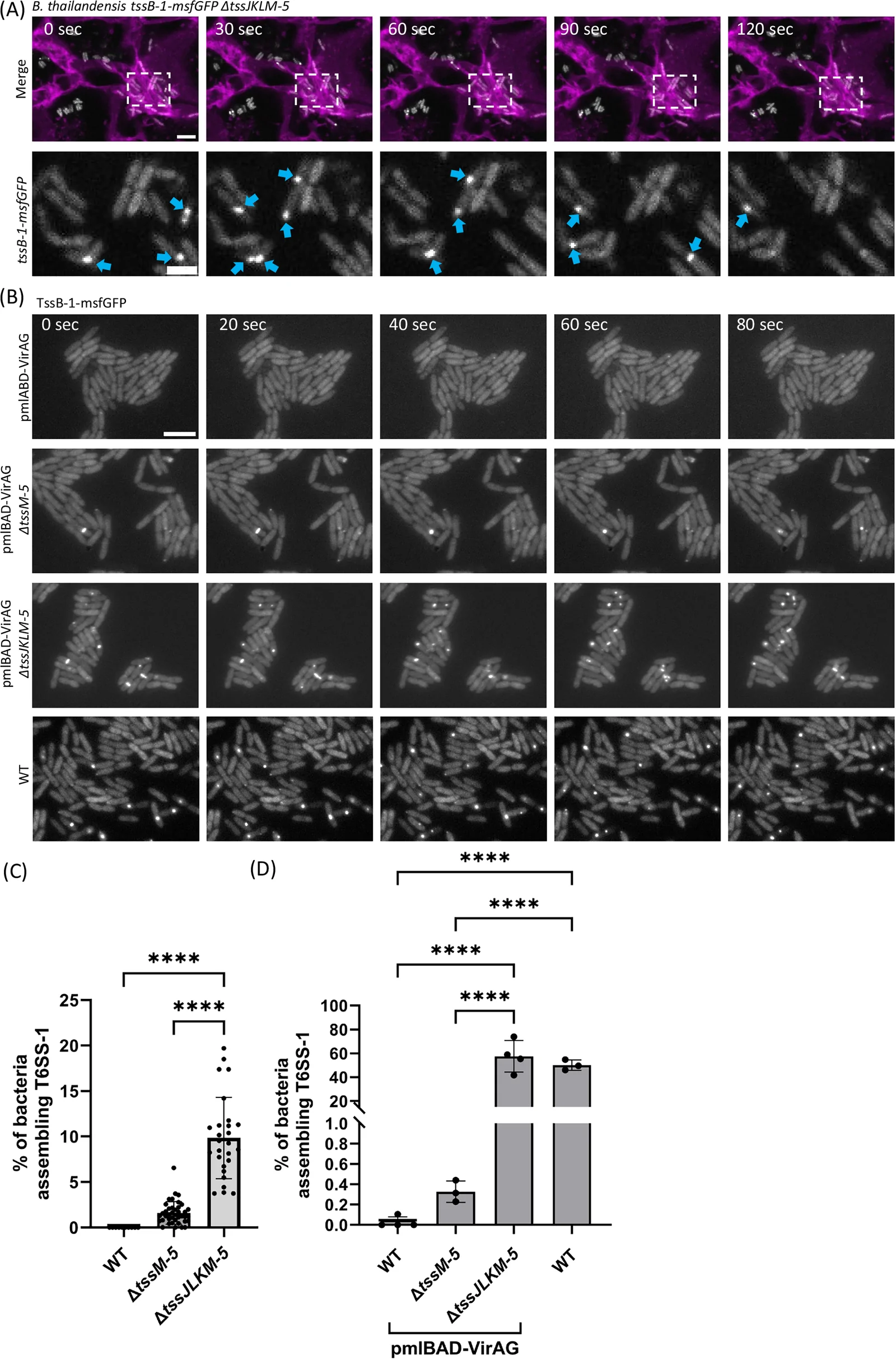
Deletion of T6SS-5 restores T6SS-1 assemblies. (**A**) Time-lapse images of A549 cells infected with *B. thailandensis* ∆*tssJLKM-5* expressing TssB-1-msfGFP (grey) at MOI of 50 at 15:30 hpi. Cells were stained with CellMask deep red (magenta) and images were acquired at 30 s intervals. Blue arrows indicate T6SS-1 assemblies. Scale bar represents 5 µm in the overview image and 2 µm in the magnified image. Images representative of at least three biological replicates. (**B**) Time-lapse microscopy of *B. thailandensis* strains on agar pads. Top row: *B. thailandensis* harbouring the arabinose-inducible plasmid pmlBAD-VirAG. Second row: *B. thailandensis* Δ*tssM-5* harbouring pmlBAD-VirAG. Third row: *B. thailandensis* Δ*tssJLKM-5* harbouring pmlBAD-VirAG. Bottom row: WT *B. thailandensis*. Overnight bacterial cultures were subcultured to mid-log phase with 0.4% L-arabinose in LB and imaged on LB agar pads. Images were acquired at 10 s intervals. Scale bar represents 5 µm. Images representative of at least three biological replicates. (**C**, **D**) Quantification of the percentage of bacteria assembling T6SS-1 (**C**) during infection, and (**D**) on agar pads. Individual bacteria were segmented with Cellpose, and the number of bacteria assembling T6SS-1 were quantified. One-way ANOVA with Tukey’s multiple comparisons test was performed and only significant pairwise comparisons are shown (****P < 0.0001). Adjusted *P* values were as follows: [group WT vs. Δ*tssM-5* (infection)], *P* = 0.2464; [group WT vs. Δ*tssJLKM-5* (infection)], *P* < 1 × 10⁻¹⁶; [group WT, pmlBAD-VirAG vs. Δ*tssM-5*, pmlBAD-VirAG (in vitro)], *P* = 0.99994; [group WT, pmlBAD-VirAG vs. Δ*tssJLKM-5*, pmlBAD-VirAG (in vitro)], *P* = 3.74 × 10⁻⁶; [group WT, pmlBAD-VirAG vs. WT *(*in vitro)], *P* = 2.62 × 10⁻⁵; [group Δ*tssM-5*, pmlBAD-VirAG vs. Δ*tssJLKM-5*, pmlBAD-VirAG (in vitro)], *P* = 7.97 ×  10⁻⁶; [group Δ*tssJLKM-5*, pmlBAD-VirAG vs. WT (in vitro)], *P* = 0.5888. Error bars represent mean ± SD (*n* = 3). Source data are available online for this figure.

**Figure EV6:**
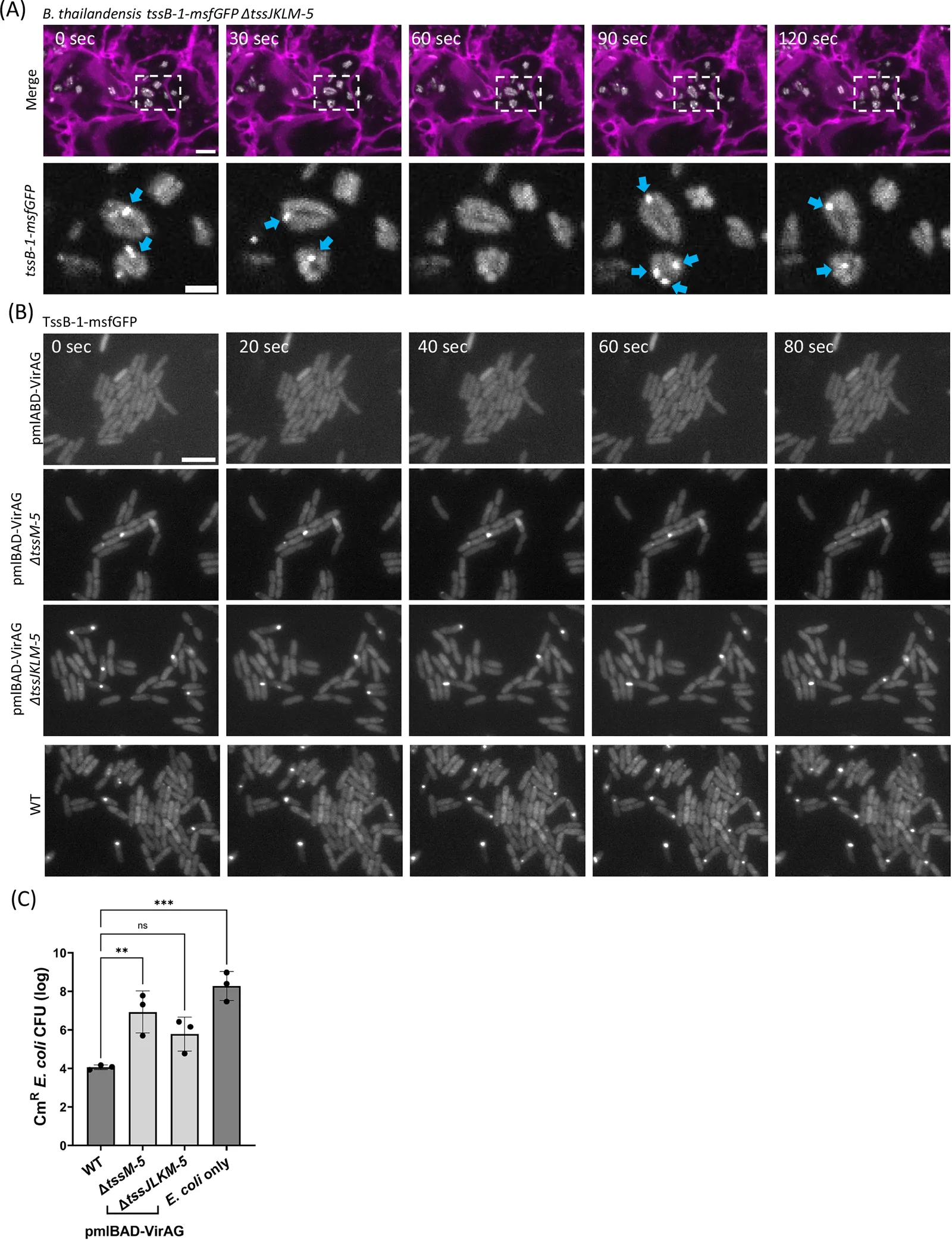
T6SS-1 assemblies are restored by deletion of T6SS-5. (**A**) Time-lapse images of A549 cells infected with *B. thailandensis* ∆*tssJLKM-5* expressing TssB-1-msfGFP (grey) at MOI of 50 at 15:40 hpi. Cells were stained with CellMask deep red (magenta) and images were acquired at 30 s intervals. Blue arrows indicate T6SS-1 assemblies. Scale bars represent 5 µm in the overview image and 2 µm in the magnified image. Images representative of at least three biological replicates. (**B**) Time-lapse microscopy of *B. thailandensis* strains on agar pads. Top row: *B. thailandensis* harbouring the arabinose-inducible plasmid pmlBAD-VirAG. Second row: *B. thailandensis* Δ*tssM-5* harbouring pmlBAD-VirAG. Third row: *B. thailandensis* Δ*tssJLKM-5* harbouring pmlBAD-VirAG. Bottom row: WT *B. thailandensis*. Overnight bacterial cultures were subcultured to mid-log phase with 0.4% L-arabinose in LB and imaged on LB agar pads. Images were acquired at 10 s intervals. Scale bar represents 5 µm. Images representative of at least three biological replicates. (**C**) Recovery of viable *E. coli* prey after competition with *B. thailandensis* strains. *E. coli* prey harbouring a plasmid conferring chloramphenicol resistance was co-cultured with *B. thailandensis* strains at 1:1 ratio on LB agar plates for 5 h at 30 degrees. The co-cultures were harvested and serially diluted and spotted on LB agar plates containing 30 µg/mL chloramphenicol. Colony forming units (CFU) of *E. coli* was plotted on a logarithmic scale. Ordinary one-way ANOVA with Dunnett’s multiple comparisons test was performed (*n* = 3, **P < 0.01, ***P < 0.001, ns = not significant). Adjusted *P* values were as follows: [group WT vs. Δ*tssM-5*, pmlBAD-VirAG], *P* = 6.0 × 10⁻³; [group WT vs. Δ*tssJLKM-5*, pmlBAD-VirAG], *P* = 7.14 × 10⁻²; [group WT vs. *E. coli*], *P* = 5.0 × 10⁻⁴. Error bars represent mean ± SD.

Since VirAG upregulates the expression of the anti-eukaryotic T6SS-5 components, we investigated if T6SS-5 components may inhibit T6SS-1 assemblies, as recently observed for *Bacteroides fragilis* (Sheahan et al, 2024). We deleted *tssM-5*, encoding T6SS-5 membrane complex component TssM-5, or *tssMJKL-5*, encoding the complete T6SS-5 membrane complex and part of the baseplate, and examined T6SS-1 assembly on agar pads and during infection. During infection of A549 cells, we observed a subpopulation of bacteria assembling T6SS-1 (Figs. 6A and EV6A; Movie EV9). To quantify this, we imaged at least 9 fields of view per biological replicate, analysing at least 3000 bacteria per strain across three biological replicates. During infection, 0% of WT bacteria assembled T6SS-1 at 15 hpi, whereas 1.5% of the Δ*tssM-5* mutant and 10% in Δ*tssJLKM-5* mutant assembled T6SS-1 (Fig. 6C).

On agar pads, the expression of VirAG inhibits T6SS-1 assemblies. However, both the Δ*tssM-5* and Δ*tssJLKM-5* mutants assembled T6SS-1 in the presence of VirAG (Figs. 6B and EV6B; Movie EV10). To quantify the change, 12 fields of view containing 1000–3000 bacteria per strain per biological replicate were analysed. The WT strain overexpressing VirAG failed to assemble T6SS-1 in two biological replicates, with one bacterium assembled T6SS-1 in the third replicate. In contrast, 0.3% of the Δ*tssM-5* mutant with VirAG overexpressed assembled T6SS-1, while 57% were assembled in Δ*tssJLKM-5* deletion strain, which is comparable to 50% of WT bacteria assembling T6SS-1 (Fig. 6D). This increase also partially restored killing of *E. coli* (Fig. EV6C).

This suggests that the expression of T6SS-5 membrane complex components inhibits T6SS-1 assemblies during infection.

### T6SS-1 activity induces mitochondrial damage

The anti-bacterial T6SS-1 effector clusters contains multiple lipases. In which BTH_I2698 was identified as the homolog of Tle1 lipase (Appendix Fig. S3A) (Majerczyk et al, 2016). We hypothesize that the secretion of T6SS-1 effector lipases could have cross-kingdom effect on the host cells. This led us to examine mitochondrial integrity as a potential intracellular target of these effectors.

**Figure 7:**
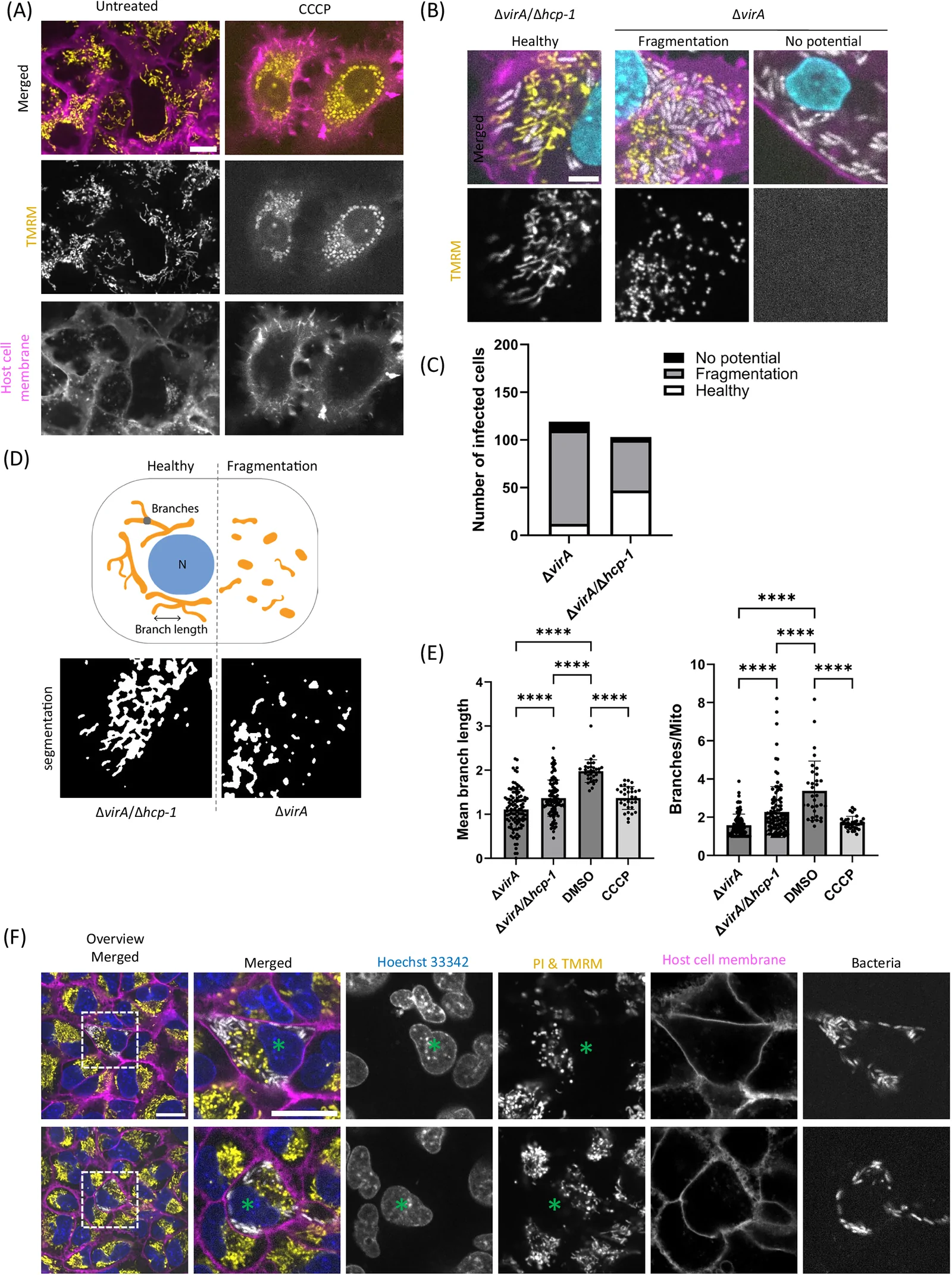
T6SS-1 activity induces mitochondrial damage. (**A**) Tetramethylrhodamine, methyl ester (TMRM) staining of A549 cells under various conditions. A549 cells were either uninfected or treated with 2 µM of CCCP for 30 min. Cells were stained with 0.1 µM of TMRM (yellow) and CellMask deep red (magenta). Scale bar represents 10 µm. Images representative of at least three biological replicates. (**B**) Example images of healthy, fragmented mitochondria, and mitochondria with no membrane potential. A549 cells were infected with *B. thailandensis* ∆*virA* or *B. thailandensis* ∆*virA /* ∆*hcp-1* (grey) at MOI 500 for 9–10 h. Cells were stained with 0.1 µM of TMRM (yellow), CellMask deep red (magenta), and Hoechst 33342 (cyan). Scale bar represents 5 µm. Images representative of at least three biological replicates. (**C**) Proportion of A549 cells infected *B. thailandensis* ∆*virA* or *B. thailandensis* ∆*virA /* ∆*hcp-1* displaying no mitochondrial membrane potential, mitochondrial fragmentation and healthy mitochondrial phenotypes. Data pooled from three biological replicates. (**D**) Schematic representation of healthy and fragmented mitochondrial morphologies illustrating the definitions of “branches per mitochondrion” and “branch length.” N denotes the nucleus. The lower panel shows examples of mitochondria segmented by MitoAnalyzer from the images in (**B**). (**E**) Automated image analysis of the same dataset from (**A**, **B**) showing the number of mitochondrial branches per mitochondria and mean mitochondrial branch length performed by the Mitochondria Analyzer plugin for ImageJ. Each data point represents one host cell. One-way ANOVA with Tukey’s multiple comparisons test was performed and *P* values were displayed (****P < 0.0001). Error bars represent mean ± SD (*n* = 3). Adjusted *P* values were as follows: [Branches per mitochondrion] Δ*virA* vs. Δ*virA/Δhcp-1*, *P* = 1.94 × 10⁻⁵; Δ*virA* vs. DMSO, *P* = 3.38 × 10⁻¹³; Δ*virA* vs. CCCP, *P* = 0.936; Δ*virA/Δhcp-1* vs. DMSO, *P* = 1.52 × 10⁻⁵; Δ*virA/Δhcp-1* vs. CCCP, *P* = 0.0402; DMSO vs. CCCP, *P* = 3.27 × 10⁻⁹. [Branch length] Δ*virA* vs. Δ*virA*/Δhcp-1, *P* = 3.59 × 10⁻⁵; ΔvirA vs. DMSO, *P* = 2.16 × 10⁻¹³; ΔvirA vs. CCCP, *P* = 0.00811; Δ*virA/Δhcp-1* vs. DMSO, *P* = 1.60 × 10⁻¹²; Δ*virA/Δhcp-1* vs. CCCP, *P* = 0.99997; DMSO vs. CCCP, *P* = 1.34 × 10⁻⁸. (**F**) Propidium iodide (PI) uptake assay of A549 cells infected with *B. thailandensis* ∆*virA* at MOI 500 at 7 hpi. Cells were stained with Hoechst 33342 (blue), PI and TMRM (yellow) and CellMask deep red (magenta). Scale bars represent 20 µm. Images representative of at least three biological replicates. Source data are available online for this figure.

To test if *B. thailandensis* T6SS-1 activity causes mitochondrial damage, we infected A549 cells with the *B. thailandensis* Δ*virA* strain that assembles T6SS-1 during infection. There was no difference in the number of bacteria (colony-forming units) in the infected cells between Δ*virA* and Δ*virA/*Δ*hcp-1* mutant (Appendix Fig. S3B,C) suggesting that the bacterial burden was comparable. To visualize mitochondria, we stained the host cells with Tetramethylrhodamine methyl ester (TMRM), a dye that is fluorescent when sequestered by mitochondria with intact membrane potential. A549 cells were infected, stained and images were taken between 9 and 10 hpi. Host cells treated with 2 μM of carbonyl cyanide 3-chlorophenylhydrazone (CCCP) was used as a positive control for mitochondrial fragmentation, and untreated uninfected cells were included as a negative control (Fig. 7A). We classified three phenotypes of mitochondrial status—healthy, fragmented mitochondria, or mitochondria with no membrane potential (Fig. 7B). Healthy mitochondria are tubular, fragmented mitochondria appear as discrete dots, and no fluorescence was detected in mitochondria without membrane potential following TMRM staining. Over 100 infected cells were analyzed for the T6SS-1 assembling Δ*virA* mutant, and the non-assembling Δ*virA/*Δ*hcp-1* mutant. For the Δ*virA* mutant, 12 infected cells displayed a normal, filamentous mitochondrial phenotype, 98 cells showed fragmentation, and 9 cells had no mitochondrial potential (Fig. 7C). For the Δ*virA/*Δ*hcp-1* mutant, 47 infected cells displayed a normal, filamentous mitochondrial phenotype, 53 cells showed fragmentation, and 3 cells had no mitochondrial potential.

To quantitatively measure mitochondrial damage, we used the ImageJ plugin Mitochondria Analyzer and quantified the number of branches per mitochondria and the mean mitochondrial branch length (Hemel et al, 2021). Fragmented mitochondria appear rounder and as isolated puncta, and thus has less branches per mitochondria and shorter branch length. Cells infected with the Δ*virA* mutant showed on average 1.5 branches per mitochondria and with a mean branch length of 1 µm (Fig. 7E). Whereas for cells infected with the Δ*virA/*Δ*hcp-1* mutant, they showed on average 2.3 branches per mitochondria and with a mean branch length of 1.4 µm. DMSO-treated uninfected host cells showed on average 3.3 branches per mitochondria and with a mean branch length of 1 µm; whereas CCCP-treated host cells showed on average 1.7 branches per mitochondria and with a mean branch length of 1.3 µm. One-way ANOVA with Tukey’s multiple comparisons test was performed and cells infected with the Δ*virA* mutant had significantly less mitochondrial branching and shorter length, indicating a higher level of mitochondrial fragmentation.

Together, our data indicate that T6SS-1 assemblies damage host cell mitochondria, leading to a higher proportion of fragmented mitochondria and mitochondria lacking membrane potential.

### T6SS-1 activity does not impact eukaryotic plasma membrane

To assess whether T6SS-1 activity affects the eukaryotic plasma membrane, we examined propidium iodide (PI) uptake in infected cells, as PI is only permeable to cells with compromised membrane integrity. A549 cells were infected with the Δ*virA* mutant for 6-7 h and stained with Hoechst 33342 to label all nuclei, PI and TMRM to assess plasma and mitochondrial membrane integrity prior to imaging. Uninfected cells treated with ethanol were included as a positive control. In this condition, mitochondrial membrane potential is lost and nuclei were PI-positive (Appendix Fig. S3D). In untreated, uninfected cells (negative control), mitochondrial morphology appeared normal and nuclei were PI-negative. We then assessed 198 infected cells across three biological replicates, and observed mitochondrial fragmentation, while host cell nuclei were PI-negative (Green asterisks, Fig. 7F). Hence, this shows that T6SS-1 assemblies do not damage eukaryotic plasma membrane.

### T6SS-1 activity triggers apoptosis

**Figure 8:**
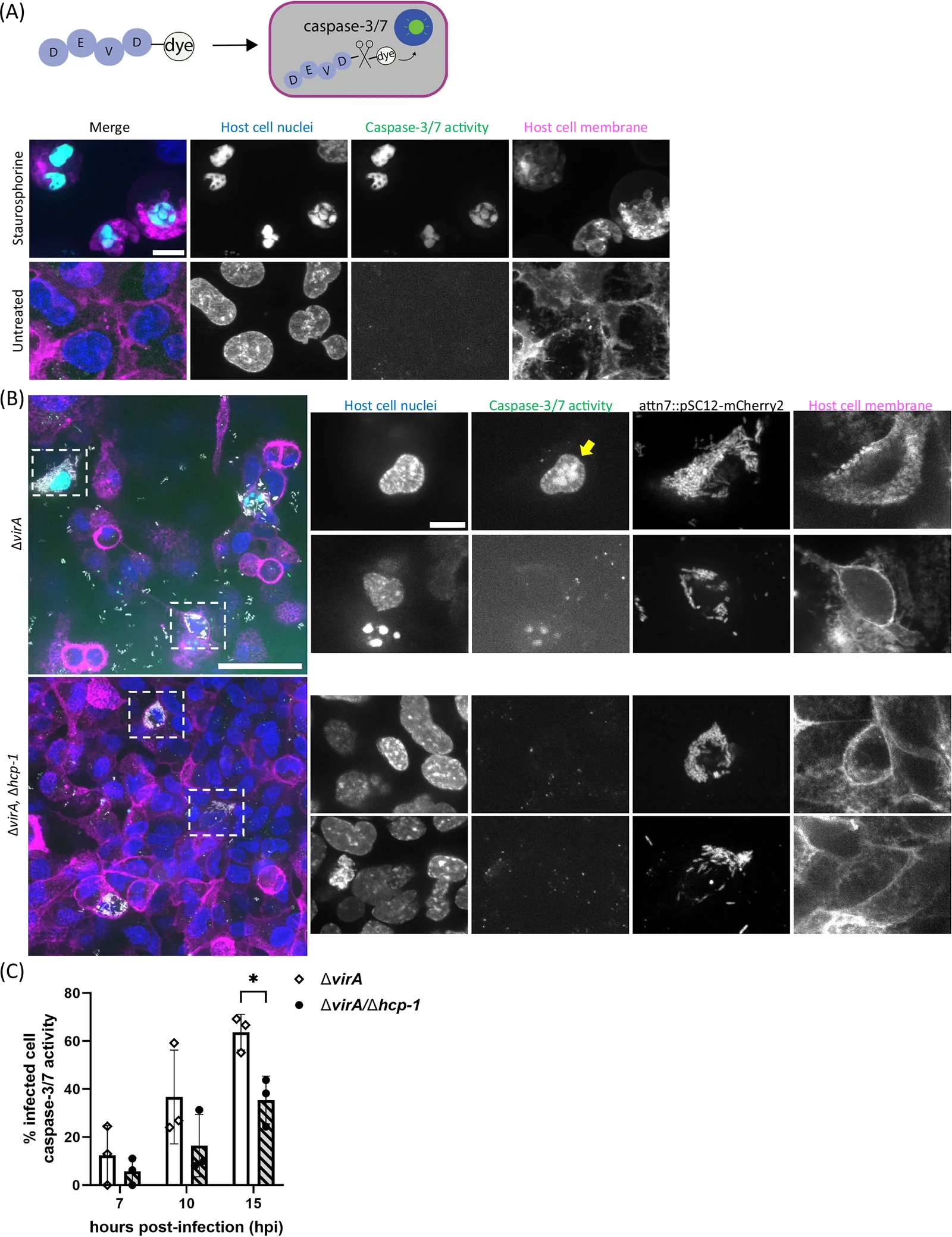
T6SS-1 activity triggers apoptotic cell death. (**A**) Scheme showing the working principle of CellEvent™ Caspase-3/7 Detection Reagent staining and example images of A549 cells that are untreated or treated with staurosporine (1 µM, 18 h) stained with CellEvent™ Caspase-3/7 Detection Reagent (green). Cells were also stained with Hoechst 33342 (blue) and CellMask deep red (magenta). Scale bar represents 10 µm. Images representative of at least three biological replicates. (**B**) Examples of A549 cells stained with CellEvent™ Caspase-3/7 Detection Reagent (green) and infected with *B. thailandensis* ∆*virA* or *B. thailandensis* ∆*virA/*∆*hcp-1* (grey) at MOI 500 for 15 h. A549 cells were also stained with Hoechst 33342 (blue) and CellMask deep red (magenta). Yellow arrow indicates host cell nuclei with active caspase-3/7 activity. Scale bars represent 50 µm in the overview image and 10 µm in the magnified image. Images representative of at least 3 biological replicates. (**C**) Quantification of the percentage of infected A549 cells displaying caspase-3/7 activity. Cells were infected with either *B. thailandensis* ∆*virA* or *B. thailandensis* ∆*virA/*∆*hcp-1* (grey) at MOI 500 for 7, 10, or 15 hpi. The total number of infected cells and the number of infected cell nuclei positive for CellEvent™ Caspase-3/7 Detection Reagent were counted. An ordinary two-way ANOVA with Bonferroni’s multiple comparisons test was performed (*P < 0.05). Adjusted *P* values are: 7 hpi, *P* = 1.00 × 10⁰; 10 hpi, *P* = 1.91 × 10⁻¹; 15 hpi, *P* = 0.0441. Error bars represent mean ± SD (*n* = 3). Source data are available online for this figure.

As mitochondrial fragmentation leads to apoptosis (Jagasia et al, 2005; Ellis, 1986), we asked if T6SS-1 activity could trigger apoptosis. We used a caspase-3/7 activity detection reagent containing a fluorophore-conjugated peptide substrate specific to caspase-3/7. Upon cleavage by active caspase-3/7, the released fluorophore intercalates with nuclear DNA and emits fluorescence. To optimize the staining conditions, a monolayer of A549 cells were either untreated or treated with staurosphorine, an apoptosis-inducing chemical, were stained with the caspase-3/7 activity detection reagent and apoptotic cells were identified (Fig. 8A).

A549 cells were infected with either the Δ*virA* mutant or the Δ*virA/*Δ*hcp-1* mutant, stained with caspase-3/7 activity detection reagent at 7, 10, 15 hpi, and images were acquired to quantify the number of apoptotic infected cells. At least 20 fields of view (302.28 × 302.28 μm) were acquired in each biological replicate. Figure 8B shows two fields of view of infected A549 cells stained with caspase-3/7 activity detection reagent. Host cell nuclei were identified by Hoechst 33342 staining, and a colocalization of the caspase-3/7 activity detection reagent signal with the nuclei was used to identify apoptotic cells. At 7 hpi and 10 hpi, there were approximately twice as many infected cells with active caspase-3/7 when infected with Δ*virA* mutant than when the cells were infected with the Δ*virA/*Δ*hcp-1* mutant in each biological replicate (Fig. 8C). Although some variability was observed in the percentage of apoptotic infected cells, by 15 hpi, 63% of the cells infected the Δ*virA* mutant are apoptotic, while only 35% of those infected with the Δ*virA/*Δ*hcp-1* mutant are apoptotic. This difference was statistically significant and indicates that infection with the T6SS-1 firing strain leads to a higher proportion of apoptotic cells than with the non-firing strain.

## Discussion

The T6SS-1 and T6SS-5 play different roles during *B. thailandensis* lifestyle. Our study showed that expression of T6SS-5 induced by the histidine kinase VirAG system in *Burkholderia* spp. inhibits T6SS-1 assemblies during infection and thus prevents T6SS-1 mediated increase in apoptosis via mitochondrial damage.

The lack of intracellular expression of T6SS-2, -4 and -6 is consistent with their unclear role in infection. In the study Heacock-Kang et al, 2021, the authors used a single-bacterium transcriptomics approach to compare the transcriptomes of individual intracellular *B. pseudomallei* with those of bacteria grown in liquid culture. Although they assessed the expression of T6SS-2, T6SS-4, and T6SS-6 under these conditions, only a limited subset of T6SS genes (1–3 components per system) was detected, indicating that no complete transcriptional activation of these T6SS clusters was observed. In addition, previous studies on T6SS-2 and T6SS-6 focused on mice infections and identified no role of the two T6SS in virulence (Schwarz et al, 2010). T6SS-4 is reported active under oxidative stress in vitro (Si et al, 2017; DeShazer, 2019). The anti-eukaryotic T6SS-5’s role in infection has been widely studied in literature (Schwarz et al, 2010, 2014; Toesca et al, 2014, 2014; Schell et al, 2007), however, the temporal dynamics of T6SS-5 expression in live cells remained uncharacterised. Moreover, the anti-bacterial T6SS-1 has primarily been studied in the context of bacterial competition assays (Schwarz et al, 2010).

Here, we identified VirAG as a switch between the T6SS-1 and T6SS-5. The VirAG dimer was reported to be reduced to its active monomer form upon exposure to host cytosolic glutathione (GSH), leading to activation of T6SS-5 expression (Wong et al, 2015). In our study, we observed T6SS-5 expression occurring also in late phagosomes before the bacteria appear in the host cytoplasm. Interestingly, we observed T6SS-5 assemblies inside late phagosomes. Although rare, this suggests that the other T6SS-5 components are also expressed at this stage, enabling the formation of functional assemblies. It is plausible that acidification and redox changes accompanying phagosome maturation are sufficient to initiate T6SS activation, which is then accelerated after bacterial escape to the cytosol. The discrepancy between previous work and out work is likely due to the fact that we focused on a different infection stage. Where previous work focused on cytosolic bacteria (6 hpi), we focused on bacteria within phagosomes (3 hpi). Moreover, in a related species *B. mallei*, T6SS expression was shown to occur prior to escape from late phagosomes, further supporting the findings of this study (Burtnick et al, 2010). Ultimately, both studies support the same overarching conclusion that VirAG is activated and T6SS-5 is expressed during infection.

We show that phagosomal maturation is important for T6SS-5 expression and is consistent with previous studies. Chua et al demonstrated that pH alkalinisation suppresses the T6SS-5 of *B. pseudomallei, B. mallei*, and *B. thailandensis*, as evidenced by the lack of MNGC formation (Chua et al, 2017). As MNGC formation is a hallmark for T6SS-5 activity in *Burkholderia*, these findings are consistent with our observation that inhibition of phagosomal acidification traps *B. thailandensis* inside vacuoles and greatly reduce T6SS-5 expression. The rare exceptions that we observed, in which *B. thailandensis* expressed T6SS-5 in bafilomycin-treated cells, may reflect incomplete inhibition of phagosomal maturation in individual host cells, or instances in which cytosolic signals leaked into the phagosome, thereby inducing T6SS-5 expression. Moreover, we concluded that *B. thailandensis* expresses T6SS-5 between 2 and 3 hpi, and the bacteria mostly are located in Rab7 and LAMP-1 positive late phagosomes. As our data represent single timepoint measurements, bacteria that do not colocalize with Rab5/7/LAMP1-positive phagosomes are presumed to be in the host cytosol, or in a phagosome not labelled by the markers mentioned above. Notably, a study by Hu et al reported that between 2 and 4 hpi, *B. pseudomallei* also co-localizes to Rab7 and LAMP-1 positive phagosomes (Hu et al, 2019). This suggests that *B. thailandensis* and *B. pseudomallei* likely share the similar intracellular trafficking dynamics.

Using Gal-3 recruitment as a marker for phagosomal rupture is common for studies of intracellular bacteria (Paz et al, 2010; Hong et al, 2021). Our observation that 8% of bacteria colocalizing with Gal-3 is consistent with a similar study by Szczesna et al (2024), which reported at comparable hpi, fewer than 10% of *B. thailandensis* colocalized with LC3, an autophagy marker recruited downstream of Gal-3 (Szczesna et al, 2024). The three exceptions of Gal-3 localization to dextran-labelled phagosomes may have resulted from transient or minor phagosomal membrane permeabilization insufficient for dextran diffusion out of the phagosome. Nevertheless, no such events were observed in BAF-treated cells, where bacteria remained confined to dextran-labelled phagosomes and showed no detectable colocalization with Gal-3.

The T6SS-1 has been shown to assemble in vitro, with several accessory proteins contributing to the regulation of T6SS assembly, whereas quorum sensing regulates the expression of T6SS effectors and the tip protein VgrG (Majerczyk et al, 2016; Lin et al, 2022). Surprisingly, we find that the T6SS-1 is expressed but not assembled during infection. The gradual decrease in T6SS-1 assemblies matching increase in expression of the anti-eukaryotic T6SS-5 indicates a switch between the two T6SS. By deleting *virA*, we found that T6SS-1 assemblies during infection is restored. Since VirAG controls the expression of T6SS-5, we explored the possibility that expression of T6SS-5 inhibiting T6SS-1 assemblies. Expression of a T6SS inhibiting another T6SS assemblies was described in *Bacteroides fragilis* (Sheahan et al, 2024). There, deletion of T6SS membrane complex genes partially restored assemblies of the other T6SS. Interestingly, deletion of *tssM-5*, one of the T6SS-5 membrane complex components, partially restored firing of T6SS-1 during infection. Whereas deletion of the full membrane complex and part of the baseplate (Δ*tssJLKM-5*) resulted in a much higher proportion of bacteria firing T6SS-1 during infection. This suggests a similar mechanism of assembly inhibition as described *B. fragilis* (Sheahan et al, 2024).

It is unclear why T6SS-1 expression would be maintained during infection. One possible explanation is that *B. thailandensis* maintains a pool of T6SS-1 proteins ready to assemble once it exits the host and re-enters the environment. This suggests that the suppression of T6SS-1 inside host cells may be a temporary, reversible adaptation. Interestingly, *B. mallei* has undergone significant genome reduction, and is believed to be evolving towards a more specialized, intracellular lifestyle (Losada et al, 2010; Moore et al, 2004). Consistent with this shift, the anti-bacterial T6SS-1 of *B. mallei* is truncated and likely non-functional (Angus et al, 2014). This hints that T6SS-1 is selected against during the evolution of *B. mallei* in the intracellular environment. Hence, we can speculate that *B. thailandensis* inhibits T6SS-1 assemblies during infection as an adaptation to surviving an intracellular environment.

The VgrGs and effectors associated with the anti-bacterial T6SS-1 were identified in three gene clusters located outside the core T6SS locus, each situated at distinct chromosomal locations (Majerczyk et al, 2016). The T6SS-1 effectors remain uncharacterised. The presence of transposase genes in effector clusters II and III suggests that these loci arose through gene duplication events (Appendix Fig. S3A). Bioinformatic analysis identified several effectors with lipase domains (BTH_I2698, BTH_I2701), and with BTH_I2691 belonging to the VasX toxin family. Intriguingly, BTH_I2698 is a homolog of Tle1, a lipase in *Pseudomonas aeruginosa* (Hu et al, 2014). Although T6SS-1 effectors are primarily considered antagonistic towards other bacteria, they may also exert transkingdom effects on eukaryotic cells. Moreover, we found that all T6SS-1 VgrGs contain the DUF2345 domain (Appendix Fig. S3A). This domain is shown to be present in the C-terminal of VgrGs from other bacteria such as VgrG2b from *Pseudomonas aeruginosa*, VgrGi of a clinical strain of *Acinetobacter baumannii*, and VgrG4 from *Klebsiella pneumonia*, all shown to contribute to virulence (Sá-Pessoa et al, 2023; Lopez et al, 2020; Berni et al, 2019).

We showed that the Δ*virA* mutant that constitutively assembles T6SS-1 during infection causes more mitochondrial damage and apoptotic cell death than the Δ*virA/*Δ*hcp-1* mutant with inactivated T6SS-1. This was not due to a difference in bacterial load in cells infected with the Δ*virA* mutant since image-based measurements and colony-forming unit analyses showed no significant differences (Appendix Fig. S3B,C). The fraction of mitochondria fragmenting in a T6SS-1 independent manner by the Δ*virA/*Δ*hcp-1* mutant may be due to impaired intracellular survival upon deletion of the virulence regulator VirAG. It is possible that the bacterium expresses virulence factors regulated by VirAG that inhibit mitochondrial damage. In addition, because Δ*virA* mutants is unable to spread from cell to cell, the increased intracellular bacterial burden may contribute to the observed phenotype. Nevertheless, by comparing a T6SS-1 assembling strain (Δ*virA)* and the non-assembling strain (Δ*virA/*Δ*hcp-1)* mutant, we have elucidated that T6SS-1 assemblies induce mitochondrial damage, and thus triggering apoptosis. It is therefore possible that *B. thailandensis* evolved an ability to inhibit T6SS-1 to prevent triggering host cell death and thus preserve its replicative niche.

Overall, our findings demonstrate that VirAG acts as a switch between the anti-eukaryotic T6SS-5 and anti-bacterial T6SS-1 of *B. thailandensis*, enabling the bacterium to better adapt when transitioning from being in the environment to being intracellular. This highlights a regulatory mechanism that governs the utilization of distinct T6SS systems in response to environmental cues.

## Methods

**Table:** Reagents and tools table

Reagent/resource	Reference or source	Identifier or catalog number
**Experimental models**		
*Burkholderia thailandensis* E264 wild-type	Ku et al 2020	N/A
*Burkholderia thailandensis Δhcp-5, tssB-5-mScarletI*	Plum et al 2024	N/A
*Burkholderia thailandensis* tssB-5-mScarlet-I	Plum et al 2024	N/A
*Burkholderia thailandensis* tssB-5-msfGFP	Plum et al 2024	N/A
*Burkholderia thailandensis* tssB-1-msfGFP	Lin et al 2022	N/A
*Burkholderia thailandensis* tssB-1-msfGFP, TssB-5-mCherry2	This study	N/A
*Burkholderia thailandensis* tssB-2-mNeonGreen	This study	N/A
*Burkholderia thailandensis* tssB-4-mNeonGreen	This study	N/A
*Burkholderia thailandensis* tssB-6-mNeonGreen	This study	N/A
*Burkholderia thailandensis* tssB-5-mScarlet-I, pmlBAD-VirAG-5	This study	N/A
*Burkholderia thailandensis BTH_I_TN7L::pCS12-mCherry2, tssB-5-msfGFP*	This study	N/A
*Burkholderia thailandensis* E264 Δ*virA*	This study	N/A
*Burkholderia thailandensis* E264 Δ*virA* Δ*hcp-1*	This study	N/A
*Burkholderia thailandensis* E264 Δ*virA*, tssB-1-msfGFP	This study	N/A
*Burkholderia thailandensis* E264 Δ*virA* Δ*hcp-1*, tssB-1-msfGFP	This study	N/A
*Burkholderia thailandensis ΔbimA, tssB-1-msfGFP*	This study	N/A
*Burkholderia thailandensis ΔtssM-5, tssB-1-msfGFP*	This study	N/A
*Burkholderia thailandensis ΔtssJLKM-5, tssB-1-msfGFP*	This study	N/A
Human: A549 cells	ATCC	Cat# 86012804 Lot# 18F020
Human: HeLa mApple-Rab5	Podinovskaia et al 2021	
Human: HeLa mApple-Rab7	Podinovskaia et al 2021	
Human: HeLa Galectin-3-GFP	Plum et al 2024	
**Recombinant DNA**		
pDONRPEX18Tp-SceI-pheS	Addgene	Plasmid#11608
pUC18T-mini-Tn7T-Tp	Addgene	Plasmid#65024
pUC18T-mini-Tn7T-Tp-*pSC12-mCherry2*	Plum et al 2024	N/A
pTNS2	Addgene	Plasmid#64968
pmlBAD	Chen et al 2011	N/A
pmlBAD-VirAG-5	This study	N/A
pDONRPEX18Tp-TssB-2-mNeonGreen	This study	N/A
pDONRPEX18Tp-TssB-4-mNeonGreen	This study	N/A
pDONRPEX18Tp-TssB-6-mNeonGreen	This study	N/A
pDONRPEX18Tp-Δ*hcp-1*	This study	N/A
pDONRPEX18Tp-Δ*virA*	This study	N/A
pDONRPEX18Tp-Δ*bimA*	This study	N/A
pDONRPEX18Tp-Δ*tssM-5*	This study	N/A
pDONRPEX18Tp-Δ*tssJLK-5*	This study	N/A
**Antibodies**		
Recombinant Anti-LAMP1 antibody (mouse)	Abcam	ab289548
Goat Anti-Mouse IgG H&L (Alexa Fluor® 647)	Abcam	ab150115
Goat anti-Rabbit IgG (H + L) Highly Cross-Adsorbed Secondary Antibody, Alexa Fluor™ Plus 647	ThermoFisher Scientific	#A32733
Goat anti-Rabbit IgG (H + L) Highly Cross-Adsorbed Secondary Antibody, Alexa Fluor™ Plus 488	ThermoFisher Scientific	#A32731
Anti-Hcp-5 Antigen sequence: MPMPCYLTLEGQTQGSIEGSCDIQGHEGKILVQAVEHTIEIPKSPQTGLPTSKRLHGPMTLTKEIDKSSPKLSQALASGEQMKSVVLEFYRILKEGKEEHYYTVKLENAILTSIRSWTPNCLVLDNKQLGHMEDVSFTYEKITWTYVPDGIEAEDSWRAPKV	GeneScript	Quote No. U9954GE260
**Oligonucleotides and other sequence-based reagents**		
TN7L_PC12-mCherry2.FOR	Plum et al 2024	TCAGTAGGGCCCTTACTTGTACAGCTCGTCCATGCC
TN7L_PC12-mCherry2.REV		AGTCATGGTACCAGCTGTTGACTCGCTTGGGATTTTCGGAATATCATGCCGGGTCTGACAATCGGATCGAGCTTCGAAAGGACAAGCATATGGTGAGCAAGGGCGAGGAGGATAAC
BTH_II_TN7_Det.FOR		GTCGGTGACGGTGGAGTAG
BTH_II_TN7_Det.Rev		TGTGAATGGTCAGACGCTGTTCG
BTH_I_TN7_Det.FOR		GCGTTCGTCGTCCACTGGGA
BTH_I_TN7_Det.Rev		CCGTGACGGTGGAGTAAAG
pmlBAD-VirAG-5_KpnI-EcoRI.FOR	This study	TACTGTTTCTCCATACCCGTTTTTTTGGGCTAGCAGGAGGAATTCTTGCTGCTGCTGTTCTACGCG
pmlBAD-VirAG-5_KpnI-EcoRI.REV		TTGCATGCCTGCAGGTCGACTCTAGAGGATCCCCGGGTACCTCATTCCCATAGCGTCTCCACGT
TssB-2-mNG-F1.F		CACGACGTTGTAAAACGACGGCCAGTCTTAAGCTCGGGCCCGACACGCATCCGGTTG
TssB-2-mNG-F1.R		ACTCCTCCTCCTGCGGCCGCGTGCTTTTCCTTCGATTCCC
TssB-2-mNG-F2.F		GGGAATCGAAGGAAAAGCACGCGGCCGCAGGAG
TssB-2-mNG-F2.R		CAGTGCTTTTCCTTCGATTCTTTGTACAGCTCATCCATACCC
TssB-2-mNG-F3.F		GTATGGATGAGCTGTACAAAGAATCGAAGGAAAAGCACTGATTC
TssB-2-mNG-F3.R		GGAAACAGCTATGACATGATTACGAATTCGAGCTCGGTACCCGGCAGGTTCTCGACGAC
TssB-2-mNG-det.1		GTTCTGATCGTCGGGCATTC
TssB-2-mNG-det.2		ATCAACCATTGCTGCCTGAAA
TssB-2-mNG-det.3		GTTTCTGGAAATCGTCGTGAT
TssB-2-mNG-det.4		GTCGGTGTTCTTTCGATGAAT
TssB-4-mNeonGreen_F1.FOR		CACGACGTTGTAAAACGACGGCCAGTCTTAAGCTCGGGCCCCGTGAGCGCGAGGTAGCG
TssB-4-mNeonGreen_F1.REV		GTATGGATGAGCTGTACAAACCGGAAGAGGGTGCG
TssB-4-mNeonGreen_F2.FOR		CATTTCGCACCCTCTTCCGGTTTGTACAGCTCATCCATACC
TssB-4-mNeonGreen_F2.REV		AACCGGAAGAGGGTGCGAAAGCGGCCGCAGGAG
TssB-4-mNeonGreen_F3.FOR		ACTCCTCCTCCTGCGGCCGCTTTCGCACCCTCTTCCG
TssB-4-mNeonGreen_F3.REV		GGAAACAGCTATGACATGATTACGAATTCGAGCTCGGTACCGGATCGTCGAGTACTTCC
TssB-4-mNG-det.For		CATCGCATAGGCGGAGTTC
TssB-4-mNG-det.Rev		CTGAGATCGAGGCAGGAAGTC
T6SS-6-mNG_F1.FOR		CACGACGTTGTAAAACGACGGCCAGTCTTAAGCTCGGGCCCGCGAACGGCCGCG
T6SS-6-mNG_F1.REV		ACTCCTCCTCCTGCGGCCGCTTCATGGCGAATCTCCGAATCG
T6SS-6-mNG_F2.FOR		ATTCGGAGATTCGCCATGAAGCGGCCGCAGGAGGAG
T6SS-6-mNG_F2.REV		TGGCGAATCTCCGAATCGTCTTTGTACAGCTCATCCATACCCATCACATC
T6SS-6-mNG_F3.FOR		GTATGGATGAGCTGTACAAAGACGATTCGGAGATTCGCC
T6SS-6-mNG_F3.REV		GGAAACAGCTATGACATGATTACGAATTCGAGCTCGGTACCGAATTCGCCCACGTATAGCGGT
TssB-6-mNG-seq.1		ACACGAGCGACTTCGAGA
TssB-6-mNG-seq.2		ATGACGTTCGAGAGCATCGAC
TssB-6-mNG-seq.3		GATGAACGAACGGCATGA
Hcp1-KO-F1.FOR		TCAGTAAAGCTTGGCACTGCACGCC
Hcp1-KO-F1.REV		GCATTTGAAGGACAAGACCTAC
Hcp1-KO-F2.FOR		GGTCTTGTCCTTCAAATGCAT
Hcp1-KO-F2.REV		TCAGTATCTAGAAGCAGATGTCGCATGT
dHcp1_chromo_det.1		CACTGCACGCCTCGAATC
dHcp1_chromo_det.end		CTCTTCAAGAAGGTGTACGAAGAA
dVirA_F1.F		CACGACGTTGTAAAACGACGGCCAGTCTTAAGCTCGGGCCCGCGTGATCGGATACGC
dVirA_F1.R		CATCGTTTCCTCTCCTGTACCGCGTAGAACAGCAGCAG
dVirA_F2.F		TGCTGCTGCTGTTCTACGCGGTACAGGAGAGGAAACGATGAATG
dVirA_F2.R		GGAAACAGCTATGACATGATTACGAATTCGAGCTCGGTACGAGCAGCCATTGCAGCG
dVirA_chromo_det_beg		CTCGACGAGGAACTTCAGC
dVirA_chromo_det_2		GATGTTGACCCGCTCTTTC
dVirA_chromo_det.end		ATGCTCGGCGAAGGAAAG
BimA-KO_F1.FOR		CACGACGTTGTAAAACGACGGCCAGTCTTAAGCTCGGGCCCCGCGCGCCGAGC
BimA-KO_F1.REV		TGTGTTCATCCATCGCACCGGGCATGAGCTGGCAATGG
BimA-KO_F2.FOR		CACCATTGCCAGCTCATGCCCGGTGCGATGGATGAACAC
BimA-KO_F2.REV		GGAAACAGCTATGACATGATTACGAATTCGAGCTCGGTACCGGCTGGTTCGGCTATGCG
BimA-KO-Det.For		CAAATGCCGCGTTGACAT
BimA-KO-Det.Rev		CATACCGATACGCCGCTGT
TssM-5-KO_F1.FOR		CACGACGTTGTAAAACGACGGCCAGTCTTAAGCTCGGGCCCGAATTGGGCCGTCAGC
TssM-5-KO_F1.REV		AGACGCTTTGCTCGATGCTCGACGGCGGCATGC
TssM-5-KO_F2.FOR		CAAGGGCGCATGCCGCCGTCGAGCATCGAGCAAAGCG
TssM-5-KO_F2.REV		GGAAACAGCTATGACATGATTACGAATTCGAGCTCGGTACCCCGCGGCTGCCGCG
TssM-5_KO_Det.FOR		GATCTCGTAGCGGCTGTTC
TssM-5_KO_Det.REV		GGATCGCCACCTGATCTATCT
pDON-TssJKL-KO-F1.F		CACGACGTTGTAAAACGACGGCCAGTCTTAAGCTCGGGCCCCATCCGCATCCTCTTGCG
pDON-TssJKL-KO-F1.R		GGGAATACCGAGTCGACCATAATCCGTCGATCGCTCATGC
pDON-TssJKL-KO-F2.F		GCATGAGCGATCGACGGATTATGGTCGACTCGGTATTCC
pDON-TssJKL-KO-F2.R		GGAAACAGCTATGACATGATTACGAATTCGAGCTCGGTACCTCGACACGATGTCGTCGA
TssJKL-KO-Det.1		CCCGAACGATGACTCACGAC
TssJKL-KO-Det.2		GTAACGGATTGCATCGGCGTC
**Chemicals, enzymes and other reagents**		
Lysozyme	Sigma-Aldrich	# L6876
Mutanolysin	Sigma-Aldrich	# M9901
DMEM high glucose	Sigma-Aldrich Chemie GmbH	# D5796
Ham’s F-12 Nutrient Mix, GlutaMAX™ Supplement	ThermoFisher Scientific	# 31765035
Gibco™ Opti-MEM™ I Reduced Serum Medium, no phenol red	Fisher Scientific	#11520386
Foetal Calf Serum (FCS), Heat Inactivated	BioConcept Ltd.	# 2-01F16-I
Bovine Serum Albumin	Sigma-Aldrich	# A9647
4-Chloro-DL-phenylalanine, 98 + %	Chemie Brunschwig AG	# ALFA13323-18
Dimethyl sulfoxide	Sigma-Aldrich	# 41639
Hoechst 33342 Solution (20 mM)	ThermoFisher	# 62249
CellMask™ Plasma Membrane Stains	ThermoFisher	# C10046
Dextran, Alexa Fluor™ 647; 10,000 MW, Anionic, Fixable	ThermoFisher	# I34361
Image-iT TMRM Reagent	Life Technologies Europe BV	# 10004265
Carbonyl cyanide 3-chlorophenylhydraz	Sigma-Aldrich Chemie	# 10002272
CellEvent™ Caspase-3/7 Detection Reagents	Life Technologies Europe BV	# C10723
Staurosphorine	Merck	# 569397
Propidium iodide	Merck	# 81845
Bafilomycin A1 Ready Made Solution	Sigma-Aldrich	# SML1661
M9, Minimal Salts, 5X	Sigma-Aldrich	# M6030
**Software**		
GraphPad Prism 9	N/A	http://www.graphpad.com
Fiji ImageJ	National Institute of Health	https://imagej.net/
FlowJo	FlowJo LLC	RRID: SCR_008520
**Other**		

### Bacterial strains, plasmids and culturing conditions

Bacterial strains, plasmids, and primers used in this study are listed in Reagents and Tools table. Bacterial strains were grown in LB media overnight at 37 °C with aeration. When appropriate, antibiotics were added at the following concentrations: 50 μg/mL of trimethoprim, 30 μg/mL of gentamicin, or 30 μg/mL of chloramphenicol for *E. coli*; and 200 μg/mL of trimethoprim, 300 μg/mL of kanamycin, or 30 μg/mL of chloramphenicol for *B. thailandensis*.

### Construction of genetic mutants and reporters

Genetic mutants were constructed via allelic exchange via the vector pDONRPEX-18Tp-SceI-pheS or pTOX (Fazli et al, 2015; Lazarus et al, 2019). The homologous flanking regions were at least 1000 bp. pDONRPEX-18Tp-SceI-pheS or pTOX derivatives were conjugated into *B. thailandensis* strains by *E. coli* SM10 λpir. For pDONRPEX-18Tp-SceI-pheS, positive conjugants were selected by trimethoprim resistance and counter selection were performed on M9 minimal medium agar plates supplemented with 0.4% glucose and 0.1% (w/v) 4-chloro-phenylalanine. For pTOX derivatives, all steps were performed in the presence of 2% glucose to prevent premature toxin induction. Conjugants were selected by chloramphenicol resistance and counter selection were performed on M9 minimal medium agar plates supplemented with 2% (w/v) rhamnose.

Bacterial strains constitutively expressing cytoplasmic fluorophores and reporter strains were constructed by chromosomal Tn7 transposase insertion (Choi et al, 2006). For constitutive expression, P_*S12*_ promoter (*Burkholderia rpsL* promoter) was employed. pUC18tminiTN7Tp and the helper plasmid pTNS2 were conjugated into *B. thailandensis* strains by *E. coli* SM10 λpir and conjugants were selected by trimethoprim resistance. Successful chromosomal insertion into *att*Tn*7* site downstream of the *glmS* gene were screened by PCR.

### Mammalian cell lines and culturing conditions

The cell lines HeLa cells (CCL-2, ATCC), A549 lung cells (CCL-185, ATCC) were used in this study. HeLa cell lines stably expressing Rab 5 and Rab 7-mApple were provided by Prof. Dr. Anne Spang (Podinovskaia et al, 2021), and HeLa cells stably expressing Galectin-3-GFP were generated previous study from our group (Plum et al, 2024). The HeLa cells were cultured in high-glucose DMEM with L-glutamine (Sigma-Aldrich) supplemented with 10% heat-inactivated fetal calf serum (FCS, BioConcept AG); and A549 lung cells with Ham’s F-12 media with GlutaMAX™ (Sigma-Aldrich) supplemented with 10% heat-inactivated FCS at 37 °C with 5% CO_2_. All experiments were performed between passages 2 and 10 and the cell lines were tested negative for mycoplasma contamination by MycoStrip (InvivoGen).

### Infection assays

Infection assays were performed as described previously (Plum et al, 2024). In brief, A549 and HeLa cells were seeded at 8 × 10^4^ cells per well on μ-slide eight well glass bottom (Ibidi GmbH) 24 h prior to infection. Bacterial strains were grown overnight in low-salt LB at 37 °C with aeration. On the day of the infection assay, overnight bacterial cultures were subcultured in a 1:100 dilution and grown to mid-log phase in low-salt LB. Both the bacterial and mammalian cells were washed twice in Opti-MEM™ Reduced Serum Medium with GlutaMAX™ supplement (Sigma-Aldrich). Bacterial cells were added to the mammalian cells at the specified MOI and the μ-slide eight well glass bottom was spun down at 300 × *g* for 5 min. After 1 h post-infection, Opti-MEM™ supplemented with 300 μg/mL of kanamycin was added to kill extracellular bacteria.

### Immunofluorescence

Cells were infected as described above. For labelling LAMP1, cells were fixed with ice cold 100% methanol for 15 min at specified timepoints. The fixed cells were then washed twice with PBS. For labelling Hcp-5, cells were fixed with ice cold 100% methanol for 5 min, washed twice with PBS, and then treated with 1 mg/mL lysozyme (Sigma-Aldrich) and 160 U/mL mutanolysin (Sigma-Aldrich) for 1 h at 37 °C with agitation. The fixed cells were then incubated with LAMP1 mouse monoclonal antibody (Abcam) or anti-Hcp-5 rabbit antibody (generated by GeneScript) were diluted to 1 μg/mL in 1% Bovine Serum Albumin (BSA, Sigma-Aldrich) at room temperature for 1 h. Cells were then washed twice with PBS. Anti-mouse or rabbit (Abcam) secondary antibodies were diluted 1:300 in 1% BSA and incubated with the cells overnight at 4 degrees. Cells were then washed twice with PBS and imaged.

### Fluorescence microscopy

Infection imaging was performed using a Nikon ECLIPSE Ti2 inverted microscope equipped with a CrestOptics X-Light V3 spinning disk confocal system and a Kinetix sCMOS camera. Images were acquired using a ×60 oil immersion objective (NA = 1.42; field of view: 302.28 × 302.28 µm) under live-cell conditions (37 °C, 5% CO₂). Unless otherwise stated, eukaryotic cells were stained with CellMask deep red (5 µg/mL) and Hoechst 33342 (40 µM) for 5 min at 37 °C with 5% CO_2._

For imaging agar pads, images were acquired on a Nikon Ti-E inverted motorized microscope with Plan Apo 100x Oil Ph3 DM (NA = 1.4; field of view – 122.64 × 122.88 μm) objective lens.

### Fluorescence intensity quantification

Time-lapse single plane images were acquired using the confocal microscopy setup described above. Bacteria were segmented with the Otsu thresholding algorithm in ImageJ. The segmented cells were saved as Regions Of Interest (ROIs), and the average fluorescence intensity within each ROI was measured for the relevant fluorescence channels. To account for background signals, fluorescence intensity was also measured from randomly selected ROIs in cell-free regions of the same channel.

### Quantification of T6SS-1 assemblies

Time-lapse imaging of infected samples was performed using the confocal microscopy setup described above, with images acquired every 30 s over a 5-min period. At each timepoint, a z-stack of three optical sections was captured with a step size of 0.5 µm. Each bacterium was imaged only once in a unique field of view to avoid duplicate counts across timepoints. The images were subsequently processed by maximum intensity projection for analysis. The total number of intracellular bacteria per field was segmented with Cellpose (Stringer et al, 2021) on the first time point, and cells assembling T6SS-1 were identified and quantified manually across the remaining time-lapse images. At least 200 bacteria were counted in each of the three biological replicates.

To quantify the proportion of bacteria assembling T6SS on agar pads, a single z-slice image of bacteria on agar pads were segmented with Cellpose to determine the total number of bacteria in each field of view. Time-lapse images were taken every 10 s over a 5-min period. For the pmlBAD-VirAG, and pmlBAD-VirAG *ΔtssM-*5 strains, which exhibited lower firing frequencies and a greater tendency to form cellular aggregates, T6SS-1 assemblies were manually quantified. With the other strains with higher firing frequencies, T6SS-1 assemblies throughout the time-lapse series were identified using the Find Maxima function in ImageJ, and bacteria containing at least one maxima spot were scored as T6SS-1 active. The ImageJ macro script is provided in Appendix. Selected images were manually inspected for quality control. At least 5000 bacteria were quantified for each strain across three biological replicates.

### Flow cytometry

On the day prior to infection, 5 × 10^5^ cells were seeded on a six-well plate. Infection was performed as described above. At 15 hpi, cells were lysed with 0.1% Triton-X 100, filtered through a 35 μm mesh, and kept on ice until analyzed. Cell analysis was conducted using a BS LSRFortessa Cell Analyzer. A total of 100,000 events were analysed per condition. Flow cytometry data were analyzed and gated using FlowJo.

### Mitochondrial damage quantification

Mitochondria were stained with Tetramethylrhodamine, Methyl Ester, Perchlorate (TMRM, Thermo Fischer) at 100 nM for 15 min at 37 °C with 5% CO_2_. For positive control, eukaryotic cells were treated with 2 µM of carbonyl cyanide m-chlorophenyl hydrazone (CCCP, Sigma-Aldrich) for 30 min. Mitochondrial morphology was quantified using the ImageJ plugin Mitochondria Analyzer (https://github.com/AhsenChaudhry/Mitochondria-Analyzer). At least 100 images per experimental condition were acquired across three biological replicates. Infected cells were manually segmented to isolate individual cells for analysis. The mitochondria were thresholded and analyzed with Mitochondria Analyzer. The number of branches per mitochondria and mean branch length were plotted for comparative analysis.

### Cell death assays

Apoptotic cell death were quantified by caspase-3/7 activity (ThermoFischer). Cells were first stained with CellMask deep red (5 µg/mL) and Hoechst 33342 (40 µM) for 5 min at 37 °C with 5% CO_2_, and washed twice with optimum containing 300 µg/mL kanamycin. CellEvent™ Caspase-3/7 green detection reagent were diluted 1:1000 from a stock concentration of 2 mM to a final working concentration of 2 µM, added directly to the same, and incubated for 30 min at 37 °C. The cells were imaged without additional washing. Green fluorescence was measured with the 470 nm/510 nm filter. As a positive control for apoptosis, cells were treated with 1 µM of staurosporine for 18 h.

To quantify propidium iodide uptake in host cells, cells were stained with propidium iodide (5 μg/mL), TMRM (100 nM), CellMask deep red (5 µg/mL) and Hoechst 33342 (40 µM) for 5 min at 37 °C with 5% CO_2_. They were then washed twice and imaged as single timepoint images. As a positive control, uninfected cells were permeabilized with 40% ethanol for 2 min prior to PI staining. In total, 198 infected cells were quantified across three biological replicates.

### Bacterial competition assay

For quantitative killing assays, mid-log phase bacteria were adjusted to an optical density at 600 nm (OD_600_) of 5. Predator and prey species were mixed at 1:1 ratio, and 20 µL of the mixture was spotted on dry LB agar plates and incubated at 30 °C for 5 h. After incubation, the spots were harvested and serially diluted for plating on selective recovery plates (chloramphenicol 30 μg/mL for *E. coli*). CFU was counted after ~18 h of incubation at 37 °C. Three independent biological replicates were analyzed.

### Image and statistical analysis

The images were analysed with ImageJ/Fiji and GraphPad Prism 9.1.0. were used for data analysis. Two-tailed *t* test was performed when comparing two means. Ordinary one-way or two-way ANOVA with Tukey’s, Bonferroni’s or Dunnett’s multiple comparison test were performed when comparing multiple means. Statistical significance was defined at *P* < 0.05 and the specific statistical testing for each experiment is listed in the figure legends.

## Supplementary information

Appendix

Peer Review File

Movie EV1

Movie EV2

Movie EV3

Movie EV4

Movie EV5

Movie EV6

Movie EV7

Movie EV8

Movie EV9

Movie EV10

Source data Fig. 1

Source data Fig. 2

Source data Fig. 3

Source data Fig. 4

Source data Fig. 5

Source data Fig. 6

Source data Fig. 7

Source data Fig. 8

Expanded View Figures

## Data Availability

This study includes no data deposited in external repositories. The source data of this paper are collected in the following database record: biostudies:S-SCDT-10_1038-S44319-026-00905-2.

## References

[CR1] Angus AA, Agapakis CM, Fong S, Yerrapragada S, Estrada-de los Santos P, Yang P, Song N, Kano S, Caballero-Mellado J, de Faria SM et al (2014) Plant-associated symbiotic burkholderia species lack hallmark strategies required in mammalian pathogenesis. PLoS ONE 9:e83779 10.1371/journal.pone.0083779 PMC3885511 24416172

[CR2] Aschtgen M, Gavioli M, Dessen A, Lloubès R, Cascales E (2010) The SciZ protein anchors the enteroaggregative *Escherichia coli* Type VI secretion system to the cell wall. Mol Microbiol 75:886–899 10.1111/j.1365-2958.2009.07028.x 20487285

[CR3] Barnes JL, Ketheesan N (2005) Route of infection in melioidosis. Emerg Infect Dis 11:638–639 10.3201/eid1104.041051 PMC3320332 15834987

[CR4] Basler M, Mekalanos JJ (2012) Type 6 secretion dynamics within and between bacterial cells. Science 337:815–815 10.1126/science.1222901 PMC3557511 22767897

[CR5] Basler M, Pilhofer M, Henderson GP, Jensen GJ, Mekalanos JJ (2012) Type VI secretion requires a dynamic contractile phage tail-like structure. Nature 483:182–186 10.1038/nature10846 PMC3527127 22367545

[CR6] Berni B, Soscia C, Djermoun S, Ize B, Bleves S (2019) A Type VI secretion system trans-kingdom effector is required for the delivery of a novel antibacterial toxin in *Pseudomonas aeruginosa*. Front Microbiol 10:1218 10.3389/fmicb.2019.01218 PMC6560169 31231326

[CR7] Birnie E, Virk HS, Savelkoel J, Spijker R, Bertherat E, Dance DAB, Limmathurotsakul D, Devleesschauwer B, Haagsma JA, Wiersinga WJ (2019) Global burden of melioidosis in 2015: a systematic review and data synthesis. Lancet Infect Dis 19:892–902 10.1016/S1473-3099(19)30157-4 PMC6867904 31285144

[CR8] Bönemann G, Pietrosiuk A, Diemand A, Zentgraf H, Mogk A (2009) Remodelling of VipA/VipB tubules by ClpV-mediated threading is crucial for Type VI protein secretion. EMBO J 28:315–325 10.1038/emboj.2008.269 PMC2646146 19131969

[CR9] Brunet YR, Zoued A, Boyer F, Douzi B, Cascales E (2015) The Type VI Secretion TssEFGK-VgrG phage-like baseplate is recruited to the TssJLM membrane complex via multiple contacts and serves as assembly platform for tail tube/sheath polymerization. PLoS Genet 11:e1005545 10.1371/journal.pgen.1005545 PMC4604203 26460929

[CR10] Burtnick MN, DeShazer D, Nair V, Gherardini FC, Brett PJ (2010) *Burkholderia mallei* cluster 1 Type VI secretion mutants exhibit growth and actin polymerization defects in RAW 264.7 murine macrophages. Infect Immun 78:88–99 10.1128/IAI.00985-09 PMC2798217 19884331

[CR11] Chantratita N, Phunpang R, Yarasai A, Dulsuk A, Yimthin T, Onofrey LA, Coston TD, Thiansukhon E, Chaisuksant S, Tanwisaid K et al (2023) Characteristics and one year outcomes of melioidosis patients in Northeastern Thailand: a prospective, multicenter cohort study. Lancet Reg Health - Southeast Asia 9:100118 10.1016/j.lansea.2022.100118 PMC9788505 36570973

[CR12] Chen JW, Murphy TL, Willingham MC, Pastan I, August JT (1985a) Identification of two lysosomal membrane glycoproteins. J Cell Biol 101:85–95 10.1083/jcb.101.1.85 PMC2113627 2409098

[CR13] Chen JW, Pan W, D’souza MP, August JT (1985b) Lysosome-associated membrane proteins: characterization of LAMP-1 of macrophage P388 and mouse embryo 3T3 cultured cells. Arch Biochem Biophys 239:574–586 10.1016/0003-9861(85)90727-1 3923938

[CR14] Chen Y, Schröder I, French CT, Jaroszewicz A, Yee XJ, Teh B-E, Toesca IJ, Miller JF, Gan Y-H (2014) Characterization and analysis of the *Burkholderia pseudomallei* BsaN virulence regulon. BMC Microbiol 14:206 10.1186/s12866-014-0206-6 PMC4236580 25085508

[CR15] Chen Y, Wong J, Sun GW, Liu Y, Tan G-YG, Gan Y-H (2011) Regulation of Type VI Secretion System during *Burkholderia pseudomallei* Infection. Infect Immun 79:3064–3073 10.1128/IAI.05148-11 PMC3147588 21670170

[CR16] Choi K-H, DeShazer D, Schweizer HP (2006) mini-Tn7 insertion in bacteria with multiple glmS-linked attTn7 sites: example *Burkholderia mallei* ATCC 23344. Nat Protoc 1:162–169 10.1038/nprot.2006.25 17406228

[CR17] Chua J, Senft JL, Lockett SJ, Brett PJ, Burtnick MN, DeShazer D, Friedlander AM (2017) pH alkalinization by chloroquine suppresses pathogenic burkholderia type 6 secretion system 1 and multinucleated giant cells. Infect Immun 85:e00586–16 10.1128/IAI.00586-16 PMC5203652 27799332

[CR18] Currie BJ, Mayo M, Ward LM, Kaestli M, Meumann EM, Webb JR, Woerle C, Baird RW, Price RN, Marshall CS et al (2021) The Darwin Prospective Melioidosis Study: a 30-year prospective, observational investigation. Lancet Infect Dis 21:1737–1746 10.1016/S1473-3099(21)00022-0 34303419

[CR19] DeShazer D (2019) A novel contact-independent T6SS that maintains redox homeostasis via Zn2+ and Mn2+ acquisition is conserved in the *Burkholderia pseudomallei* complex. Microbiol Res 226:48–54 10.1016/j.micres.2019.05.007 31284944

[CR20] Durand E, Nguyen VS, Zoued A, Logger L, Péhau-Arnaudet G, Aschtgen M-S, Spinelli S, Desmyter A, Bardiaux B, Dujeancourt A et al (2015) Biogenesis and structure of a Type VI secretion membrane core complex. Nature 523:555–560 10.1038/nature14667 26200339

[CR21] Ellis H (1986) Genetic control of programmed cell death in the nematode *C. elegans*. Cell 44:817–829 10.1016/0092-8674(86)90004-8 3955651

[CR22] Fazli M, Harrison JJ, Gambino M, Givskov M, Tolker-Nielsen T (2015) In-frame and unmarked gene deletions in *Burkholderia cenocepacia* via an allelic exchange system compatible with gateway technology. Appl Environ Microbiol 81:3623–3630 10.1128/AEM.03909-14 PMC4421043 25795676

[CR23] Felisberto-Rodrigues C, Durand E, Aschtgen M-S, Blangy S, Ortiz-Lombardia M, Douzi B, Cambillau C, Cascales E (2011) Towards a structural comprehension of bacterial Type VI secretion systems: characterization of the TssJ-TssM complex of an *Escherichia coli* pathovar. PLoS Pathog 7:e1002386 10.1371/journal.ppat.1002386 PMC3213119 22102820

[CR24] Filloux A, Freemont P (2016) Structural biology: baseplates in contractile machines. Nat Microbiol 1:16104 10.1038/nmicrobiol.2016.104 27572982

[CR25] Haraga A, West TE, Brittnacher MJ, Skerrett SJ, Miller SI (2008) *Burkholderia thailandensis* as a model system for the study of the virulence-associated Type III secretion system of *Burkholderia pseudomallei*. Infect Immun 76:5402–5411 10.1128/IAI.00626-08 PMC2573339 18779342

[CR26] Heacock-Kang Y, McMillan IA, Norris MH, Sun Z, Zarzycki-Siek J, Bluhm AP, Cabanas D, Norton RE, Ketheesan N, Miller JF et al (2021) The Burkholderia pseudomallei intracellular ‘TRANSITome. Nat Commun 12:1907 10.1038/s41467-021-22169-1 PMC7998038 33772012

[CR27] Hemel IMGM, Engelen BPH, Luber N, Gerards M (2021) A hitchhiker’s guide to mitochondrial quantification. Mitochondrion 59:216–224 10.1016/j.mito.2021.06.005 34102326

[CR28] Hong M-H, Weng I-C, Li F-Y, Lin W-H, Liu F-T (2021) Intracellular galectins sense cytosolically exposed glycans as danger and mediate cellular responses. J Biomed Sci 28:16 10.1186/s12929-021-00713-x PMC7931364 33663512

[CR29] Hood RD, Singh P, Hsu F, Güvener T, Carl MA, Trinidad RRS, Silverman JM, Ohlson BB, Hicks KG, Plemel RL et al (2010) A Type VI secretion system of *Pseudomonas aeruginosa* targets a toxin to bacteria. Cell Host Microbe 7:25–37 10.1016/j.chom.2009.12.007 PMC2831478 20114026

[CR30] Hu H, Zhang H, Gao Z, Wang D, Liu G, Xu J, Lan K, Dong Y (2014) Structure of the Type VI secretion phospholipase effector Tle1 provides insight into its hydrolysis and membrane targeting. Acta Crystallogr D Biol Crystallogr 70:2175–2185 10.1107/S1399004714012899 25084336

[CR31] Hu Z, Rao C, Tang M, Zhang Y, Lu X, Chen J, Mao C, Deng L, Li Q, Mao X (2019) Rab32 GTPase, as a direct target of miR-30b/c, controls the intracellular survival of *Burkholderia pseudomallei* by regulating phagosome maturation. PLoS Pathog 15:e1007879 10.1371/journal.ppat.1007879 PMC6594657 31199852

[CR32] Hughes R (1999) Secretion of the galectin family of mammalian carbohydrate-binding proteins. Biochim Biophys Acta BBA - Gen Subj 1473:172–185 10.1016/s0304-4165(99)00177-4 10580137

[CR33] Huynh KK, Eskelinen E-L, Scott CC, Malevanets A, Saftig P, Grinstein S (2007) LAMP proteins are required for fusion of lysosomes with phagosomes. EMBO J 26:313–324 10.1038/sj.emboj.7601511 PMC1783450 17245426

[CR34] Jagasia R, Grote P, Westermann B, Conradt B (2005) DRP-1-mediated mitochondrial fragmentation during EGL-1-induced cell death in *C. elegans*. Nature 433:754–760 10.1038/nature03316 15716954

[CR35] Jia J, Claude-Taupin A, Gu Y, Choi SW, Peters R, Bissa B, Mudd MH, Allers L, Pallikkuth S, Lidke KA et al (2020) Galectin-3 coordinates a cellular system for lysosomal repair and removal. Dev Cell 52:69–87.e8 10.1016/j.devcel.2019.10.025 PMC6997950 31813797

[CR36] Kapitein N, Bönemann G, Pietrosiuk A, Seyffer F, Hausser I, Locker JK, Mogk A (2013) ClpV recycles VipA/VipB tubules and prevents non-productive tubule formation to ensure efficient Type VI protein secretion. Mol Microbiol 87:1013–1028 10.1111/mmi.12147 23289512

[CR37] Kitano M, Nakaya M, Nakamura T, Nagata S, Matsuda M (2008) Imaging of Rab5 activity identifies essential regulators for phagosome maturation. Nature 453:241–245 10.1038/nature06857 18385674

[CR38] Kostow N, Welch MD (2022) Plasma membrane protrusions mediate host cell–cell fusion induced by *Burkholderia thailandensis*. Mol Biol Cell 33:ar70 10.1091/mbc.E22-02-0056 PMC9635284 35594178

[CR83] Ku JWK, Chen Y, Lim BJW, Gasser S, Crasta KC, Gan YH (2020) Bacterial-induced cell fusion is a danger signal triggering cGAS-STING pathway via micronuclei formation. Proc Natl Acad Sci USA 117:15923–15934 10.1073/pnas.2006908117 PMC7355030 32571920

[CR39] Kudryashev M, Wang RY-R, Brackmann M, Scherer S, Maier T, Baker D, DiMaio F, Stahlberg H, Egelman EH, Basler M (2015) Structure of the Type VI secretion system contractile sheath. Cell 160:952–962 10.1016/j.cell.2015.01.037 PMC4359589 25723169

[CR40] Lazarus JE, Warr AR, Kuehl CJ, Giorgio RT, Davis BM, Waldor MK (2019) A new suite of allelic-exchange vectors for the scarless modification of proteobacterial genomes. Appl Environ Microbiol 85:e00990–19 10.1128/AEM.00990-19 PMC6677854 31201277

[CR41] Lennings J, Makhlouf M, Olejnik P, Mayer C, Brötz-Oesterhelt H, Schwarz S (2019) Environmental and cellular factors affecting the localization of T6SS proteins in *Burkholderia thailandensis*. Int J Med Microbiol 309:151335 10.1016/j.ijmm.2019.151335 31378704

[CR42] Limmathurotsakul D, Golding N, Dance DAB, Messina JP, Pigott DM, Moyes CL, Rolim DB, Bertherat E, Day NPJ, Peacock SJ et al (2016) Predicted global distribution of *Burkholderia pseudomallei* and burden of melioidosis. Nat Microbiol 1:15008 10.1038/nmicrobiol.2015.8 27571754

[CR43] Limmathurotsakul D, Wongratanacheewin S, Teerawattanasook N, Wongsuvan G, Chaisuksant S, Chetchotisakd P, Chaowagul W, Day NPJ, Peacock SJ (2010) Increasing incidence of human melioidosis in Northeast Thailand. Am Soc Trop Med Hyg 82:1113–1117 10.4269/ajtmh.2010.10-0038 PMC2877420 20519609

[CR44] Lin L, Capozzoli R, Ferrand A, Plum M, Vettiger A, Basler M (2022) Subcellular localization of Type VI secretion system assembly in response to cell–cell contact. EMBO J 41:e108595 10.15252/embj.2021108595 PMC9251886 35634969

[CR45] Lopez J, Ly PM, Feldman MF (2020) The tip of the VgrG Spike is essential to functional Type VI secretion system assembly in *Acinetobacter baumannii*. mBio 11:e02761–19 10.1128/mBio.02761-19 PMC6960284 31937641

[CR46] Losada L, Ronning CM, DeShazer D, Woods D, Fedorova N, Kim HS, Shabalina SA, Pearson TR, Brinkac L, Tan P et al (2010) Continuing evolution of *Burkholderia mallei* through genome reduction and large-scale rearrangements. Genome Biol Evol 2:102–116 10.1093/gbe/evq003 PMC2839346 20333227

[CR47] Ma AT, McAuley S, Pukatzki S, Mekalanos JJ (2009) Translocation of a *Vibrio cholerae* Type VI secretion effector requires bacterial endocytosis by host cells. Cell Host Microbe 5:234–243 10.1016/j.chom.2009.02.005 PMC3142922 19286133

[CR48] Majerczyk C, Schneider E, Greenberg EP (2016) Quorum sensing control of Type VI secretion factors restricts the proliferation of quorum-sensing mutants. eLife 5:e14712 10.7554/eLife.14712 PMC4868534 27183270

[CR49] Matsumoto N, Nakanishi-Matsui M (2019) Proton pumping V-ATPase inhibitor bafilomycin A1 affects Rab7 lysosomal localization and abolishes anterograde trafficking of osteoclast secretory lysosomes. Biochem Biophys Res Commun 510:421–426 10.1016/j.bbrc.2019.01.118 30717974

[CR50] Mauvezin C, Neufeld TP (2015) Bafilomycin A1 disrupts autophagic flux by inhibiting both V-ATPase-dependent acidification and Ca-P60A/SERCA-dependent autophagosome-lysosome fusion. Autophagy 11:1437–1438 10.1080/15548627.2015.1066957 PMC4590655 26156798

[CR51] Meumann EM, Limmathurotsakul D, Dunachie SJ, Wiersinga WJ, Currie BJ (2023) *Burkholderia pseudomallei* and melioidosis. Nat Rev Microbiol 22:155–169 10.1038/s41579-023-00972-5 37794173

[CR52] Moore RA, Reckseidler-Zenteno S, Kim H, Nierman W, Yu Y, Tuanyok A, Warawa J, DeShazer D, Woods DE (2004) Contribution of gene loss to the pathogenic evolution of *Burkholderia pseudomallei* and *Burkholderia mallei*. Infect Immun 72:4172–4187 10.1128/IAI.72.7.4172-4187.2004 PMC427422 15213162

[CR53] Nazarov S, Schneider JP, Brackmann M, Goldie KN, Stahlberg H, Basler M (2018) Cryo- EM reconstruction of Type VI secretion system baseplate and sheath distal end. EMBO J 37:e97103 10.15252/embj.201797103 PMC5813253 29255010

[CR54] Paz I, Sachse M, Dupont N, Mounier J, Cederfur C, Enninga J, Leffler H, Poirier F, Prevost M-C, Lafont F et al (2010) Galectin-3, a marker for vacuole lysis by invasive pathogens. Cell Microbiol 12:530–544 10.1111/j.1462-5822.2009.01415.x 19951367

[CR55] Pietrosiuk A, Lenherr ED, Falk S, Bönemann G, Kopp J, Zentgraf H, Sinning I, Mogk A (2011) Molecular basis for the unique role of the AAA+ chaperone ClpV in Type VI protein secretion. J Biol Chem 286:30010–30021 10.1074/jbc.M111.253377 PMC3191042 21733841

[CR56] Plum MTW, Cheung HC, Iscar PR, Chen Y, Gan Y-H, Basler M (2024) *Burkholderia thailandensis* uses a Type VI secretion system to lyse protrusions without triggering host cell responses. Cell Host Microbe 32:676–692.e5 10.1016/j.chom.2024.03.013 38640929

[CR57] Podinovskaia M, Prescianotto-Baschong C, Buser DP, Spang A (2021) A novel live-cell imaging assay reveals regulation of endosome maturation. eLife 10:e70982 10.7554/eLife.70982 PMC8635980 34846303

[CR58] Pukatzki S, Ma AT, Revel AT, Sturtevant D, Mekalanos JJ (2007) Type VI secretion system translocates a phage tail spike-like protein into target cells where it cross-links actin. Proc Natl Acad Sci USA 104:15508–15513 10.1073/pnas.0706532104 PMC2000545 17873062

[CR59] Pukatzki S, Ma AT, Sturtevant D, Krastins B, Sarracino D, Nelson WC, Heidelberg JF, Mekalanos JJ (2006) Identification of a conserved bacterial protein secretion system in *Vibrio cholerae* using the *Dictyostelium* host model system. Proc Natl Acad Sci USA 103:1528–1533 10.1073/pnas.0510322103 PMC1345711 16432199

[CR60] Rhodes KA, Schweizer HP (2016) Antibiotic resistance in Burkholderia species. Drug Resist Updat 28:82–90 10.1016/j.drup.2016.07.003 PMC5022785 27620956

[CR61] Sanchez-Villamil JI, Tapia D, Borlee GI, Borlee BR, Walker DH, Torres AG (2020) *Burkholderia pseudomallei* as an enteric pathogen: identification of virulence factors mediating gastrointestinal infection. Infect Immun 89:e00654–20 10.1128/IAI.00654-20 PMC7927940 33106293

[CR62] Sá-Pessoa J, López-Montesino S, Przybyszewska K, Rodríguez-Escudero I, Marshall H, Ova A, Schroeder GN, Barabas P, Molina M, Curtis T et al (2023) A trans-kingdom T6SS effector induces the fragmentation of the mitochondrial network and activates innate immune receptor NLRX1 to promote infection. Nat Commun 14:871 10.1038/s41467-023-36629-3 PMC9935632 36797302

[CR63] Schell MA, Ulrich RL, Ribot WJ, Brueggemann EE, Hines HB, Chen D, Lipscomb L, Kim HS, Mrázek J, Nierman WC et al (2007) Type VI secretion is a major virulence determinant in *Burkholderia mallei*: Type VI secretion in *B. mallei* virulence. Mol Microbiol 64:1466–1485 10.1111/j.1365-2958.2007.05734.x 17555434

[CR64] Schwarz S, Singh P, Robertson JD, LeRoux M, Skerrett SJ, Goodlett DR, West TE, Mougous JD (2014) VgrG-5 is a Burkholderia Type VI secretion system-exported protein required for multinucleated giant cell formation and virulence. Infect Immun 82:1445–1452 10.1128/IAI.01368-13 PMC3993412 24452686

[CR65] Schwarz S, West TE, Boyer F, Chiang W-C, Carl MA, Hood RD, Rohmer L, Tolker-Nielsen T, Skerrett SJ, Mougous JD (2010) Burkholderia Type VI secretion systems have distinct roles in eukaryotic and bacterial cell interactions. PLoS Pathog 6:e1001068 10.1371/journal.ppat.1001068 PMC2928800 20865170

[CR66] Sheahan ML, Flores K, Coyne MJ, García-Bayona L, Chatzidaki-Livanis M, Holst AQ, Smith RC, Sundararajan A, Barquera B, Comstock LE (2024) A ubiquitous mobile genetic element changes the antagonistic weaponry of a human gut symbiont. Science 386:414–420 10.1126/science.adj9504 PMC12132083 39446952

[CR67] Shneider MM, Buth SA, Ho BT, Basler M, Mekalanos JJ, Leiman PG (2013) PAAR-repeat proteins sharpen and diversify the Type VI secretion system spike. Nature 500:350–353 10.1038/nature12453 PMC3792578 23925114

[CR68] Si M, Zhao C, Burkinshaw B, Zhang B, Wei D, Wang Y, Dong TG, Shen X (2017) Manganese scavenging and oxidative stress response mediated by Type VI secretion system in *Burkholderia thailandensis*. Proc Natl Acad Sci USA 114:E2233–E2242 10.1073/pnas.1614902114 PMC5358365 28242693

[CR69] Stevens MP, Stevens JM, Jeng RL, Taylor LA, Wood MW, Hawes P, Monaghan P, Welch MD, Galyov EE (2005) Identification of a bacterial factor required for actin-based motility of Burkholderia pseudomallei: actin-based motility of *B. pseudomallei*. Mol Microbiol 56:40–53 10.1111/j.1365-2958.2004.04528.x 15773977

[CR70] Stevens MP, Wood MW, Taylor LA, Monaghan P, Hawes P, Jones PW, Wallis TS, Galyov EE (2002) An Inv/Mxi-Spa-like Type III protein secretion system in *Burkholderia pseudomallei* modulates intracellular behaviour of the pathogen: role of a TTSS in *B. pseudomallei*-host cell interactions. Mol Microbiol 46:649–659 10.1046/j.1365-2958.2002.03190.x 12410823

[CR71] Stringer C, Wang T, Michaelos M, Pachitariu M (2021) Cellpose: a generalist algorithm for cellular segmentation. Nat Methods 18:100–106 10.1038/s41592-020-01018-x 33318659

[CR72] Szczesna M, Huang Y, Lacoursiere RE, Bonini F, Pol V, Koc F, Ward B, Geurink PP, Pruneda JN, Thurston TLM (2024) Bacterial esterases reverse lipopolysaccharide ubiquitylation to block host immunity. Cell Host Microbe 32:913–924.e7 10.1016/j.chom.2024.04.012 PMC11271751 38870903

[CR73] Szwedziak P, Pilhofer M (2019) Bidirectional contraction of a type six secretion system. Nat Commun 10:1565 10.1038/s41467-019-09603-1 PMC6450956 30952865

[CR74] Toesca IJ, French CT, Miller JF (2014) The Type VI secretion system spike protein VgrG5 mediates membrane fusion during intercellular spread by Pseudomallei group Burkholderia species. Infect Immun 82:1436–1444 10.1128/IAI.01367-13 PMC3993413 24421040

[CR76] Vieira OV, Bucci C, Harrison RE, Trimble WS, Lanzetti L, Gruenberg J, Schreiber AD, Stahl PD, Grinstein S (2003) Modulation of Rab5 and Rab7 recruitment to phagosomes by phosphatidylinositol 3-kinase. Mol Cell Biol 23:2501–2514 10.1128/MCB.23.7.2501-2514.2003 PMC150733 12640132

[CR77] Whiteley L, Meffert T, Haug M, Weidenmaier C, Hopf V, Bitschar K, Schittek B, Kohler C, Steinmetz I, West TE et al (2017) Entry, intracellular survival, and multinucleated-giant-cell-forming activity of *Burkholderia pseudomallei* in human primary phagocytic and nonphagocytic cells. Infect Immun 85:e00468–17 10.1128/IAI.00468-17 PMC5607410 28760929

[CR78] Wiersinga WJ, van der Poll T, White NJ, Day NP, Peacock SJ (2006) Melioidosis: insights into the pathogenicity of *Burkholderia pseudomallei*. Nat Rev Microbiol 4:272–282 10.1038/nrmicro1385 16541135

[CR79] Wong J, Chen Y, Gan Y-H (2015) Host cytosolic glutathione sensing by a membrane histidine kinase activates the Type VI secretion system in an intracellular bacterium. Cell Host Microbe 18:38–48 10.1016/j.chom.2015.06.002 26094804

[CR80] Wood TE, Howard SA, Wettstadt S, Filloux A (2019) PAAR proteins act as the ‘sorting hat’ of the Type VI secretion system. Microbiology 165:1203–1218 10.1099/mic.0.000842 PMC7376260 31380737

[CR81] Yamamoto A, Tagawa Y, Yoshimori T, Moriyama Y, Masaki R, Tashiro Y (1998) Bafilomycin A1 prevents maturation of autophagic vacuoles by inhibiting fusion between autophagosomes and lysosomes in rat hepatoma cell line, H-4-II-E cells. Cell Struct Funct 23:33–42 10.1247/csf.23.33 9639028

[CR82] Zhang J, Zeng W, Han Y, Lee W-R, Liou J, Jiang Y (2023) Lysosomal LAMP proteins regulate lysosomal pH by direct inhibition of the TMEM175 channel. Mol Cell 83:2524–2539.e7 10.1016/j.molcel.2023.06.004 PMC10528928 37390818

