# Supplementary material for: Regulation of Type VI Secretion Systems of Burkholderia during transition to intracellular lifestyle
Source: EMBO Rep. 2026 Sep 2;27(19):5659–93. doi: 10.1038/s44319-026-00905-2 (PMC13650128; doi:10.1038/s44319-026-00905-2)
Supplement: Supplementary file 1 [file 44319_2026_905_MOESM1_ESM.pdf]

Appendix

Regulation of Type VI Secretion Systems of *Burkholderia* during transition to intracellular lifestyle

Table of contents

Appendix Figure S#1	1
Figure legend for Figure S#1	2
Appendix Figure S#2	3
Figure legend for Figure S#2	4
Appendix Figure S#3	5
Figure legend for Figure S#3	6
Fiji macro script	7-8

Appendix Figure S#1

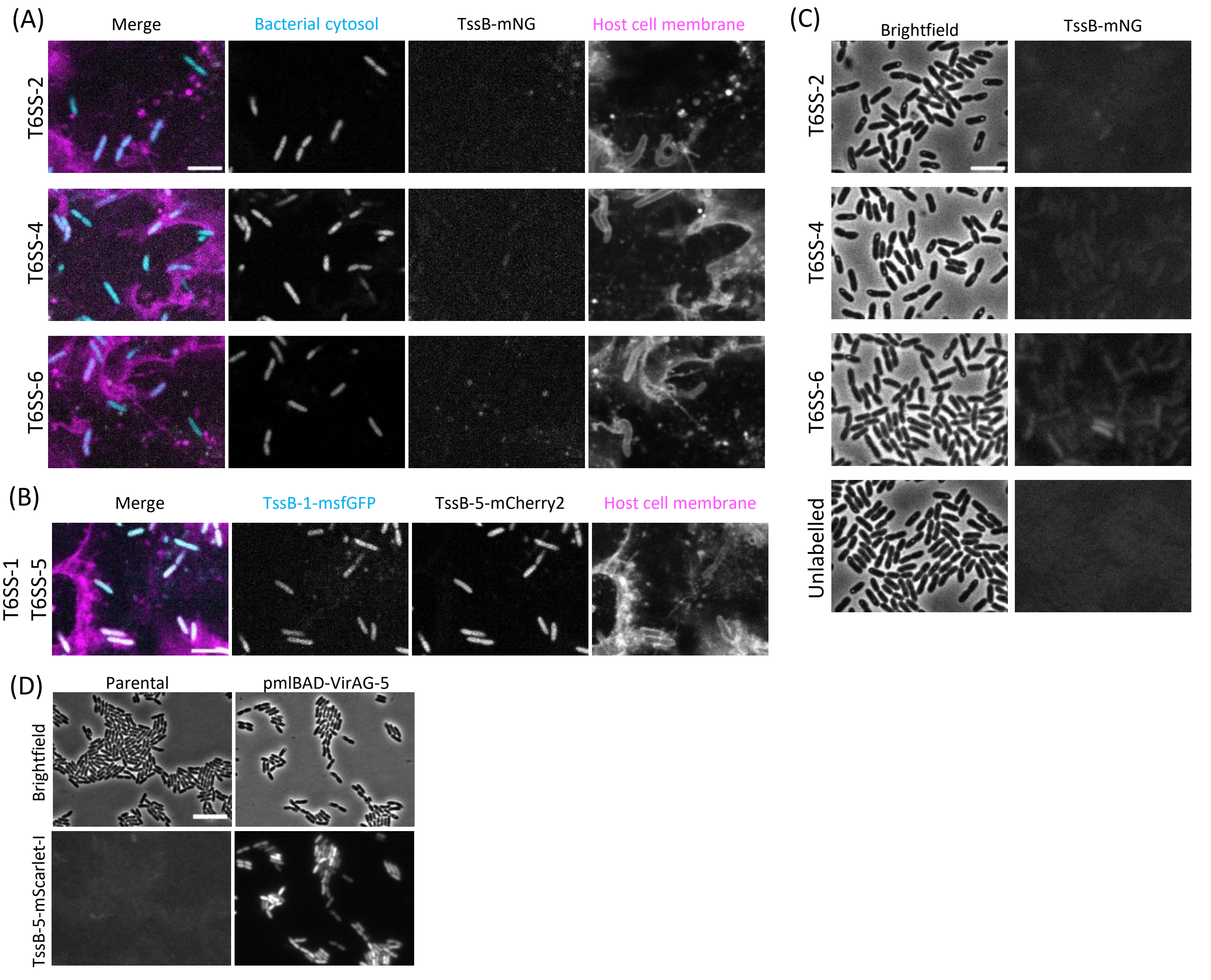

Appendix Figure S#1. Expression of *B. thailandensis* T6SS. (A,B) Representative confocal fluorescence images of *B. thailandensis* E264 infection in A549 cells. Host cell membranes are visualised with CellMask deep red stain (magenta). Images were taken between 11 to 12 hpi and representative of three biological replicates. **(A)** The sheath protein TssB of T6SS-2, T6SS-4 and T6SS-6 were fused with mNeonGreen (mNG) and were shown in grey. The fluorophore mCherry2 was expressed under the *B. thailandensis* ribosomal pSC12 promoter at a mini-Tn7 insertion site on the bacterial chromosome for visualisation and localisation of bacteria in confocal microscopy (cyan). **(B)** TssB-1-msfGFP was shown in cyan and TssB-5-mScarlet-I was shown in grey. Scale bars represent 5 µm. **(C)** Wide-field agar pad microscopy of *B. thailandensis* E264 strains shown in (A). Overnight bacterial cultures were subcultured to mid-log phase in LB, adjusted to OD 10, and spotted on LB agar pads. The sheath protein TssB of T6SS-2, T6SS-4 and T6SS-6 were fused with mNeonGreen (mNG) and were shown in grey. Images were adjusted to the same contrast settings and representative of 3 biological replicates. Scale bar represents 5 µm. **(D)** Wide-field agar pad microscopy of parental *B. thailandensis* with chromosomal TssB-5-mScarlet-I and *B. thailandensis* with chromosomal TssB-5-mScarlet-I harbouring the arabinose-inducible plasmid pmlBAD-VirAG. Overnight bacterial cultures were subcultured to mid-log phase in LB supplemented with 0.4% L-arabinose. The cultures were adjusted to OD 10 and spotted on LB agar pads. Scale bar represents 10 µm. Images representative of at least 3 biological replicates.

Appendix Figure S#2

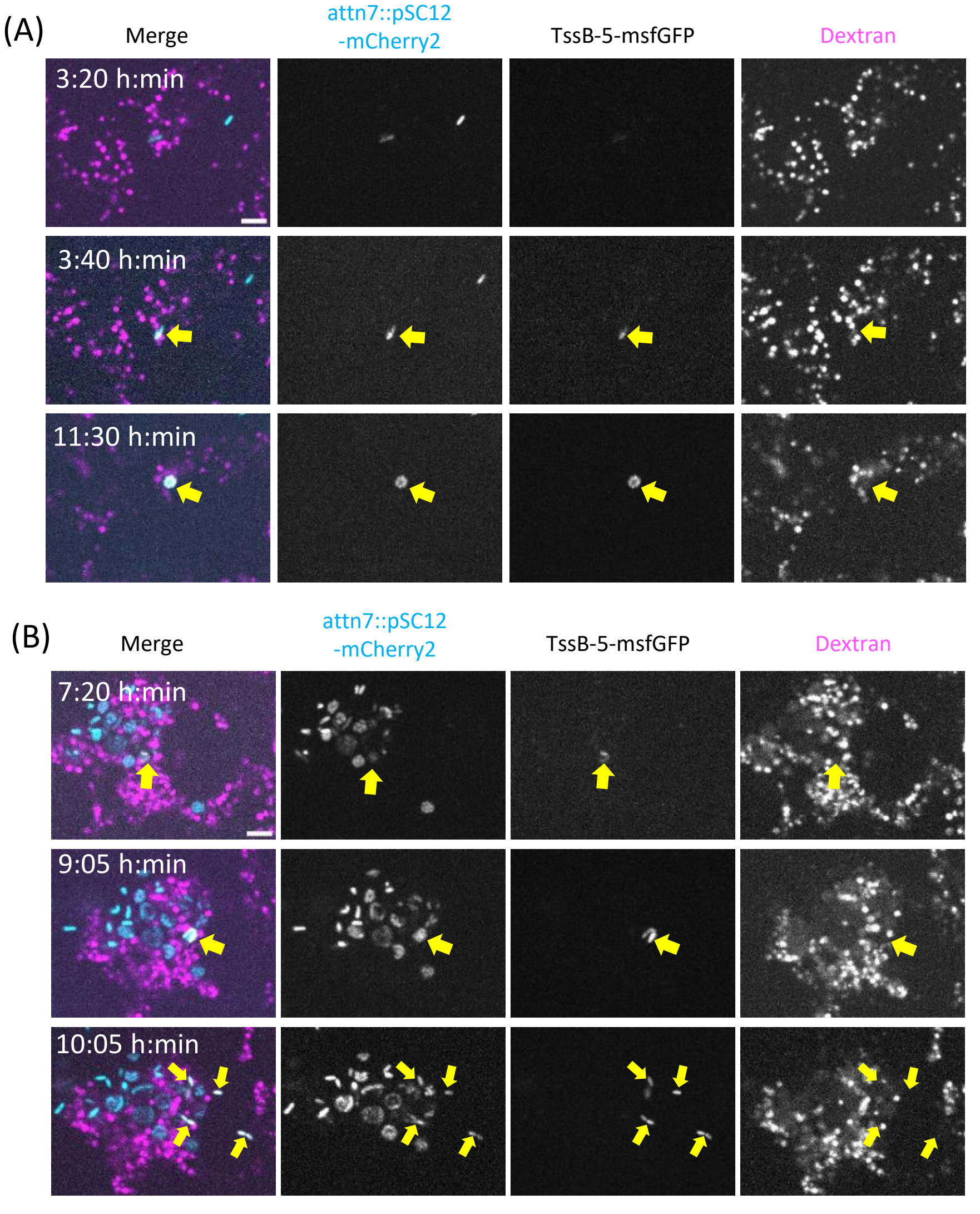

Appendix Figure S#2. Representative examples of T6SS-5 expression upon bafilomycin A1 treatment. Examples of bacterial clusters that (A) remain within the phagosome and (B) escape from the phagosome. Time-lapse imaging of A549 cells treated with bafilomycin A1 (100nM) or DMSO infected with B. thailandensis expressing bacterial cytosolic mCherry2 under ribosomal promoter pSC12 (cyan) and TssB-5-msfGFP (grey) at MOI of 200. A549 cells were pre-treated with 0.25 mg/mL of fluorescent dextran (magenta) for 18 hours to identify intra-phagosomal bacteria. Host cells were pre-treated with either bafilomycin A1 or DMSO 1 hour before infection, infected with B. thailandensis for 1 hour without treatment, and bafilomycin A1 or DMSO was added and kept in the media with 300 µg/mL kanamycin from 1 hpi onwards. Images were taken at 5-minute intervals. Yellow arrows indicate bacteria that express T6SS-5. Scale bars represent 5 µm. Images representative of at least 3 biological replicates.

Appendix Figure S#3

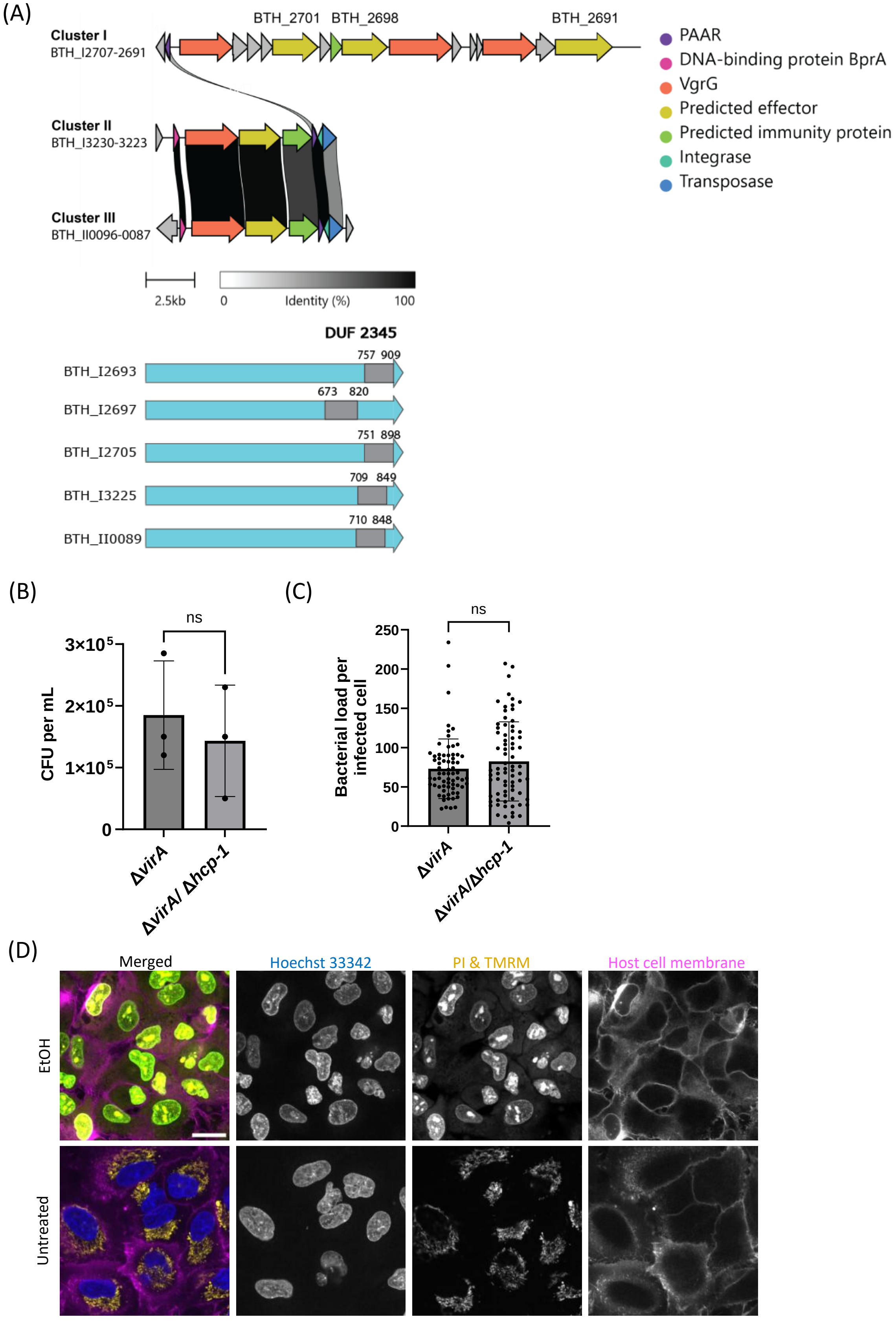

Appendix Figure S#3. Genomic organization of the T6SS-1 cluster, bacterial burden, and propidium iodide uptake in infected cells. (A) Genomic clusters of the T6SS-1 components. Spike protein VgrGs are colour-coded blue, PAAR motif in green, and transposon elements in red. Synteny was determined by CAGEcat(Gilchrist & Chooi, 2021). **(B)** Colony forming units (CFU) and the number of bacteria per A549 cells infected with *B. thailandensis* ∆*virA,* or *B. thailandensis* ∆*virA /* ∆*hcp-1*. Infected cells were lysed at 15 hpi with 0.1% triton X-100 and the lysate was serially diluted and plated on LB plate for counting CFU. Number of bacteria per infected cell was manually quantified based on the dataset in Figure 7. Data represents three biological replicates. Unpaired t-test with Welch's corrections was performed (ns = not significant, P=0.5973). Error bars represent mean ± SD. **(C)** Number of bacteria per infected cell. A549 cells were infected with either *B. thailandensis* ∆*virA,* or *B. thailandensis* ∆*virA /* ∆*hcp-1*. Images were acquired between 9-10 hpi, and the number of bacteria per infected cell were manually counted. Each point represents an infected. Data pooled from three biological replicates. Unpaired t-test with Welch's corrections was performed (ns = not significant, P=0.2086). Error bars represent mean ± SD. **(D)** Propidium iodide (PI) uptake assay of uninfected A549 cells treated with ethanol or untreated. Cells were stained with Hoechst 33342 (blue), PI and TMRM (yellow) and CellMask deep red (magenta). Scale bar represents 20 µm. Images representative of 3 biological replicates.

Fiji macro script

Quantification of percentage of T6SS-1 firing bacteria on agar pads

```
// Ensure an image and ROI Manager are open
if (nImages == 0 || roiManager("count") == 0) {
   exit("Error: Please open your time-lapse image and populate the ROI Manager first.");
}

// Get basic image dimensions
getDimensions(width, height, channels, slices, frames);

nFrames = frames;
if (nFrames == 1 && slices > 1)
   nFrames = slices; // Handle Z vs T naming bugs

nCells = roiManager("count");
origID = getImageID();

setBatchMode(true);

// Array to track cell activity:
// 0 = No firing
// 1 = Fired at least once
cellFiredStatus = newArray(nCells);
// Loop through each frame
for (f = 1; f <= nFrames; f++) {

   selectImage(origID);
   setSlice(f);

   // Find maxima points
   run("Find Maxima...",
      "prominence=1000 output=[Single Points] strict");

   maximaMapID = getImageID();

   // Check each cell ROI
   for (c = 0; c < nCells; c++) {

      // Skip cells already marked as active
      if (cellFiredStatus[c] == 1)
         continue;

      selectImage(maximaMapID);
      roiManager("select", c);

      getStatistics(area, mean, min, max);
```

```
// A maximum value of 255 indicates a spot
      if (max == 255)
         cellFiredStatus[c] = 1;
   }

   // Close temporary maxima image
   selectImage(maximaMapID);
   close();
}

setBatchMode(false);
// Calculate results
totalFiredCells = 0;

for (i = 0; i < nCells; i++) {
    if (cellFiredStatus[i] == 1)
        totalFiredCells++;
}

percentage = (totalFiredCells / nCells) * 100;

// Print results
print("\\Clear");
print("--- T6SS Activity Analysis ---");
print("Total Bacteria Segmented: " + nCells);
print("Active Bacteria (>=1 T6SS spot): " + totalFiredCells);
print("Activity Rate: " + d2s(percentage, 2) + "%");
```
