# Supplementary material for: Regulation of Type VI Secretion Systems of Burkholderia during transition to intracellular lifestyle
Source: EMBO Rep. 2026 Sep 2;27(19):5659–93. doi: 10.1038/s44319-026-00905-2 (PMC13650128; doi:10.1038/s44319-026-00905-2)
Supplement: Supplementary file 2 [file 44319_2026_905_MOESM21_ESM.pdf]

Expanded View Figures

Figure EV1. Expression of the anti-eukaryotic T6SS-5 structural and regulatory components in vitro and during infection.

(**A**) Immunofluorescence staining of Hcp-5. A549 cells were infected with various *B. thailandensis* strains at MOI 500 and fixed with methanol at 3 hpi. The slide was then permeabilized with lysozyme and mutanolysin for 1 h at 37 degrees. Then stained with anti-hcp-5 antibody at 1 μg/mL for 1 h at room temperature. Then the slide was washed and stained with anti-rabbit-AlexaFluor488 or 647 overnight at 4 degrees. Scale bar represents 2 μm. Yellow arrows indicate Hcp-5 expression. Images representative of at least three biological replicates. (**B**) Time-lapse imaging of A549 cells infected with *B. thailandensis* expressing bacterial cytosolic mCherry2 under ribosomal promoter pSC12 (cyan) and TssB-5-msfGFP (grey) at MOI of 50. Host cell membrane was stained with CellMask deep red (magenta). Yellow arrows indicate host cell membrane around the bacteria. Scale bars represent 5 μm in the overview image and 5 μm in the magnified image. (**C**) Fluorescence intensity of various channels during the infection period shown in (**B**). Bacteria were segmented and tracked, and fluorescence intensities of TssB-5-msfGFP (T6SS-5, in blue), bacterial cytosol (pSC12-mCherry2, in grey), and background (black) were plotted. A two-way ANOVA with Tukey's multiple comparisons test was performed (***$P < 0.0001$). Adjusted P values for the comparison between reporter and background signals at each time point were: 160 min, $P = 4.34 \times 10^{-8}$; 165 min, $P = 1.4 \times 10^{-14}$; 170 min, $P < 1 \times 10^{-15}$. Error bars represent mean ± SD. (**D**) Time-lapse imaging of A549 cells infected with *B. thailandensis* expressing bacterial cytosolic mCherry2 under ribosomal promoter pSC12 (cyan) and TssB-5-msfGFP (grey) at MOI of 200. Host cells were pre-treated with 0.25 mg/mL of fluorescent dextran for 18 h (magenta). Yellow arrows indicate bacteria expressing T6SS-5 when inside of a dextran-labelled vacuole. Scale bars represent 5 μm in the overview image and 5 μm in the magnified image. (**E**) Fluorescence intensity of various channels during the infection period shown in (**D**). Bacteria were segmented and tracked, and fluorescence intensities of TssB-5-msfGFP (T6SS-5, in blue), bacterial cytosol (pSC12-mCherry2, in grey), and fluorescent dextran (magenta) were plotted.

 © The Author(s)

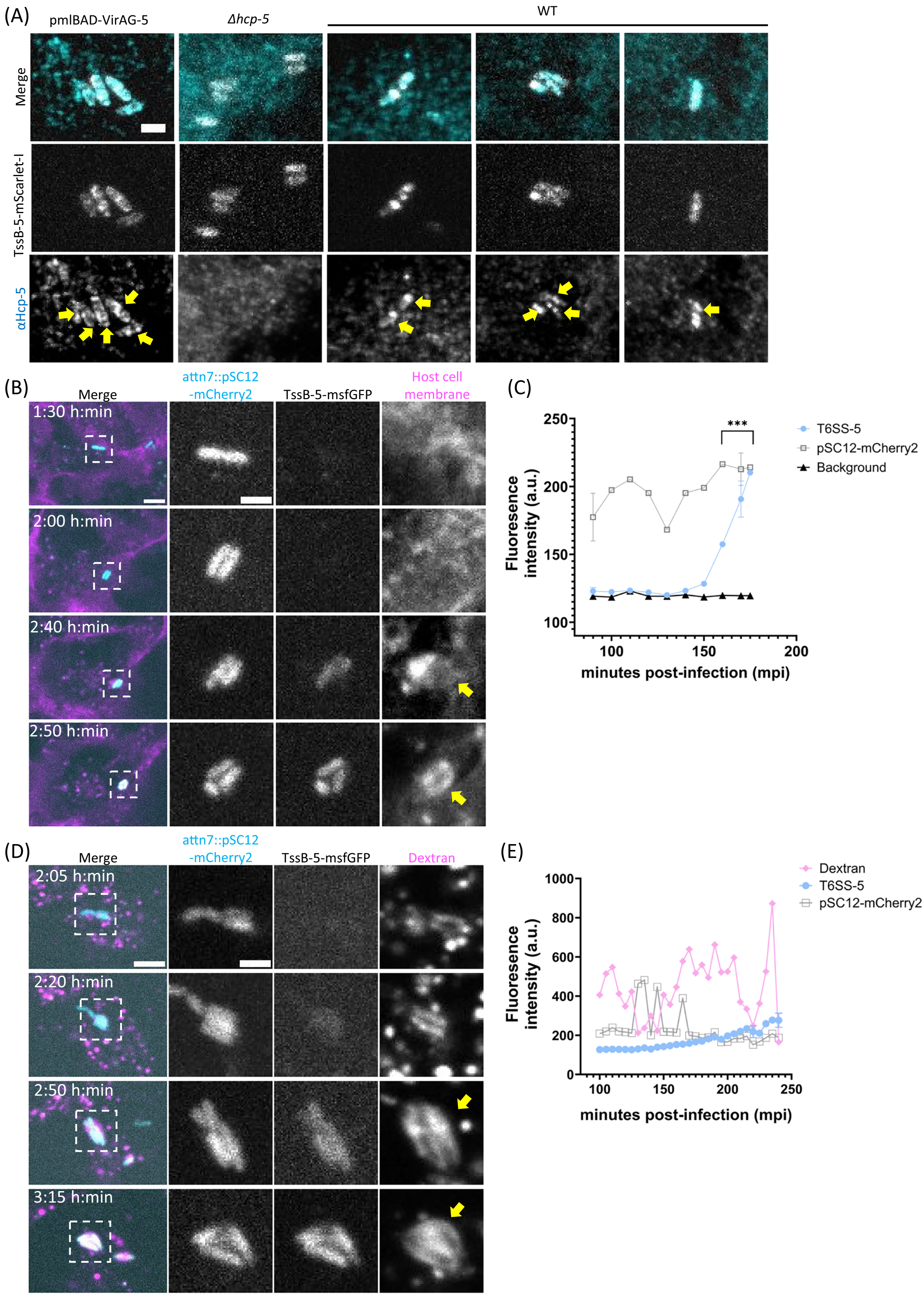
(A)
pmlBAD-VirAG-5
Δhcp-5
WT
Merge
TssB-5-mScarlet-I
αHcp-5
(B)
Merge
attn7::pSC12
-mCherry2
TssB-5-msfGFP
Host cell
membrane
1:30 h:min
2:00 h:min
2:40 h:min
2:50 h:min
(C)
***
T6SS-5
pSC12-mCherry2
Background
Fluoresence
intensity (a.u.)
minutes post-infection (mpi)
(D)
Merge
attn7::pSC12
-mCherry2
TssB-5-msfGFP
Dextran
2:05 h:min
2:20 h:min
2:50 h:min
3:15 h:min
(E)
Dextran
T6SS-5
pSC12-mCherry2
Fluoresence
intensity (a.u.)
minutes post-infection (mpi)

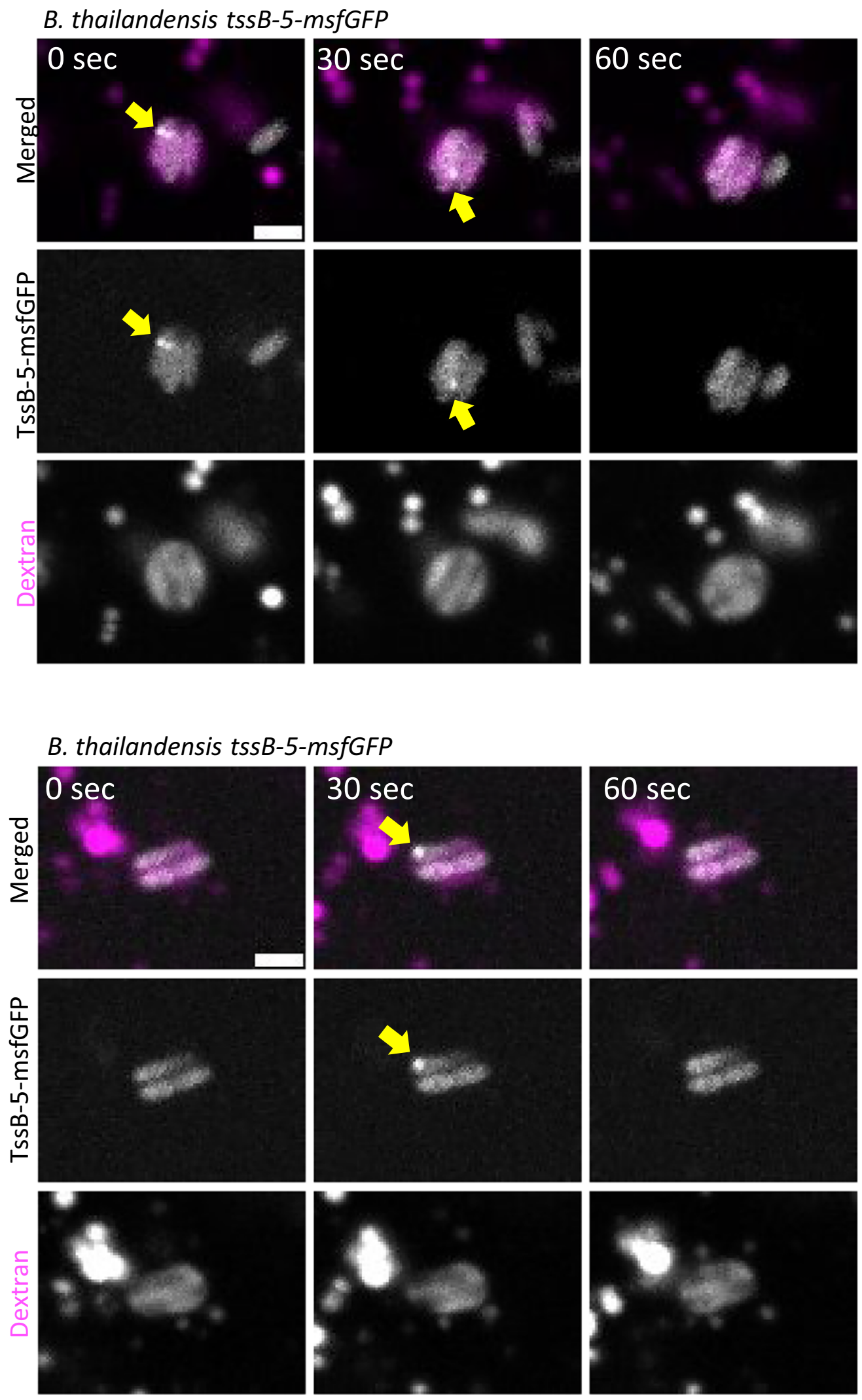

Figure EV2. T6SS-5 assemblies in phagosome.

Two examples of *B. thailandensis* assembling T6SS-5 (grey) at 3 hpi inside of a dextran-labelled vacuole (magenta) during infection of A549 cells. A549 cells were pre-treated with 0.25 mg/mL of fluorescent dextran to identify bacteria inside phagosomes. Yellow arrows indicate a T6SS-5 assembly event. Scale bar represents 2 μm. Images representative of at least three biological replicates.

 © The Author(s)

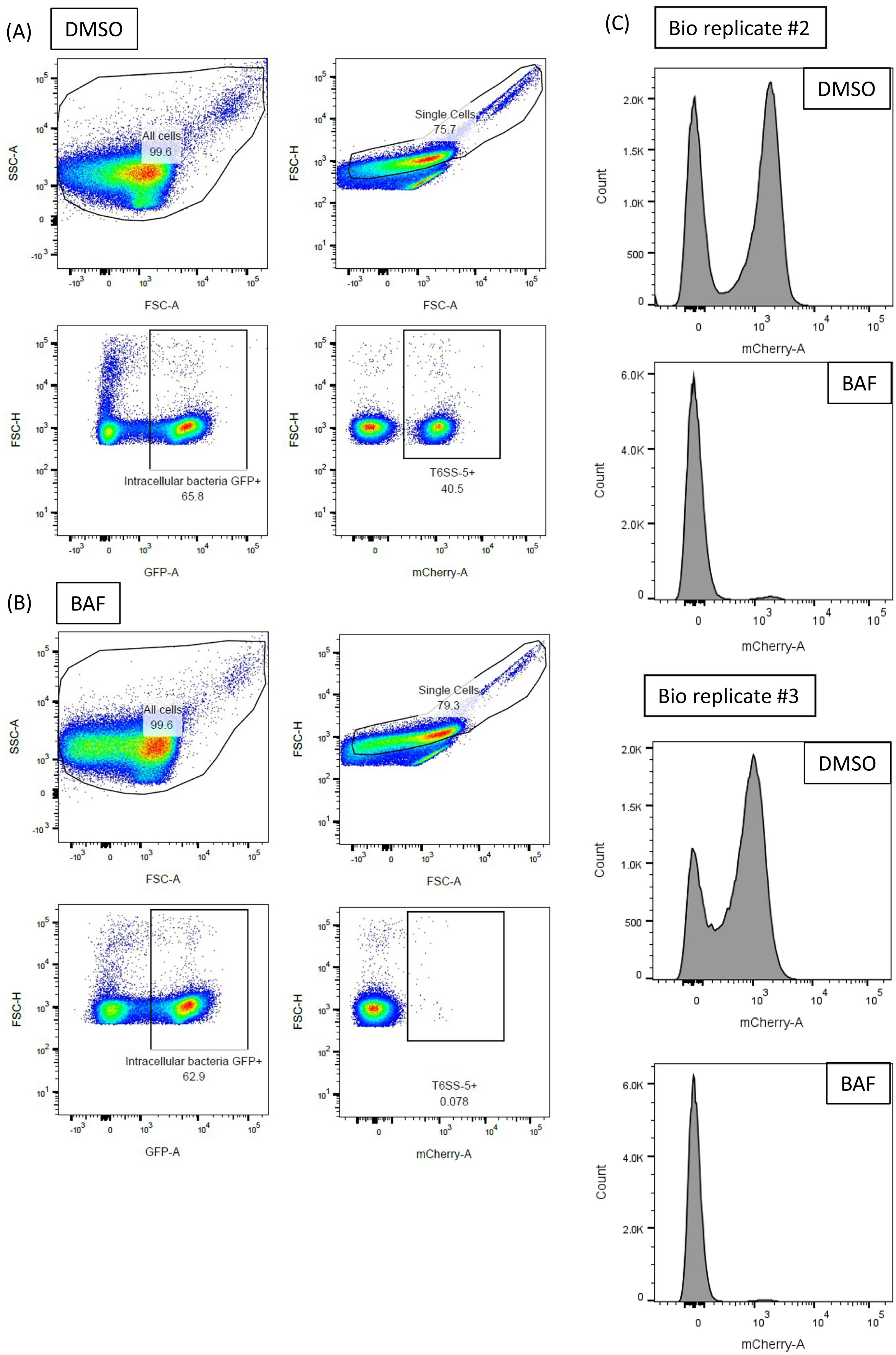
(A)
DMSO
All cells
99.6
SSC-A
FSC-A
Single Cells
75.7
FSC-H
Intracellular bacteria GFP+
65.8
GFP-A
T6SS-5+
40.5
mCherry-A
(B)
BAF
All cells
99.6
Single Cells
79.3
Intracellular bacteria GFP+
62.9
T6SS-5+
0.078
(C)
Bio replicate #2
DMSO
BAF
Count
mCherry-A
Bio replicate #3
DMSO
BAF

Figure EV3. Flow cytometry gating strategy and biological replicates.

Representative gating strategy for bacteria in (**A**) DMSO-treated, or (**B**) bafilomycin A1 (BAF) treated A549 cells. Cells were sequentially gated on singlets and mNeonGreen-positive bacteria. Bacteria constitutively expressing bacterial cytosolic mNeonGreen from a ribosomal promoter (Tn7 insertion) were identified, and the proportion of TssB-5-mScarlet-I-positive bacteria was quantified using the mCherry channel during flow cytometry. (**C**) Histograms showing the proportion of TssB-5-mScarlet-I positive bacteria in DMSO-treated or BAF-treated cells from two independent biological replicates. In total, 100,000 events per conditions were recorded.

 © The Author(s)

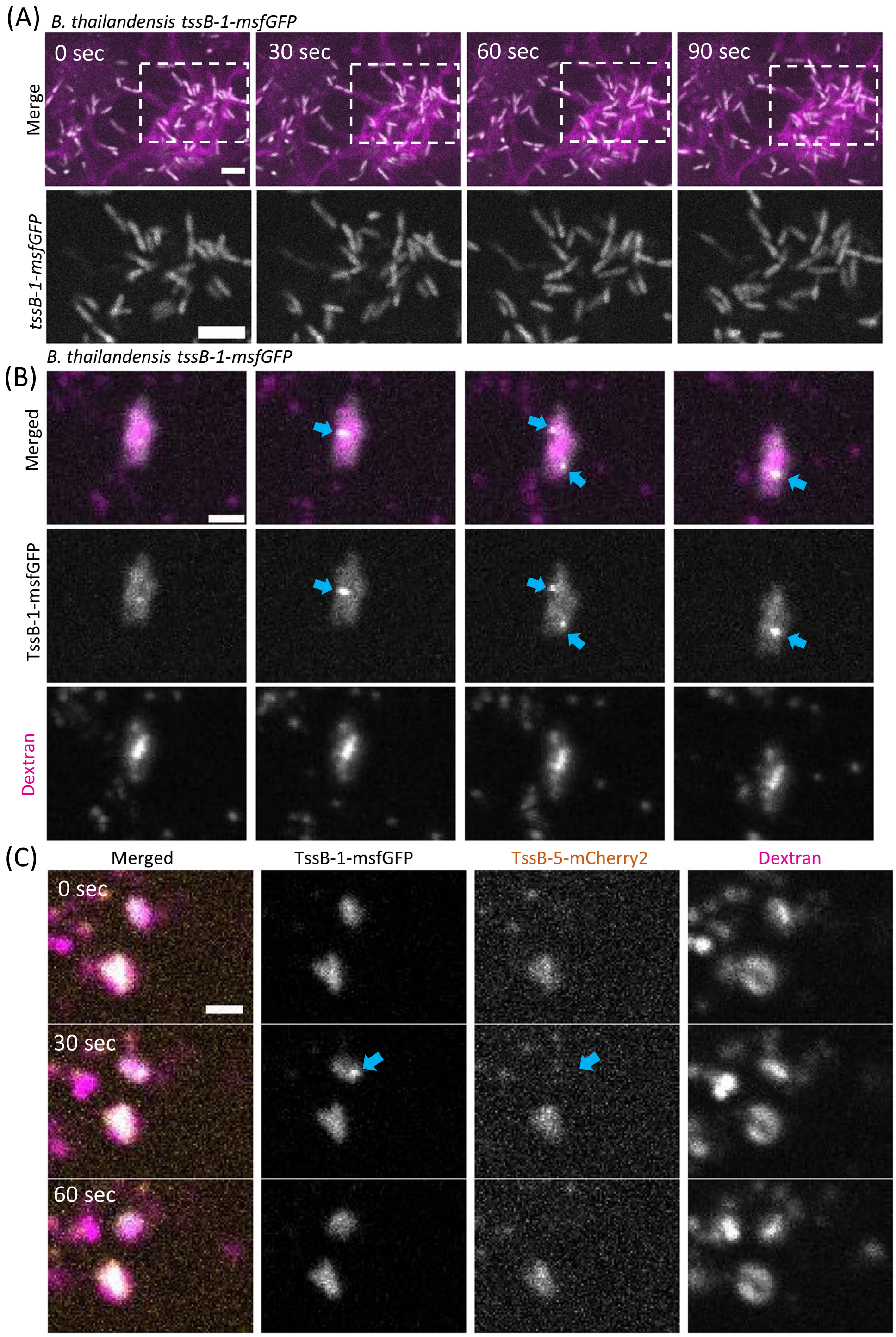

Figure EV4. T6SS-1 assembly dynamics during infection.

(**A**) Time-lapse microscopy of A549 cells infected with *B. thailandensis* TssB-1-msfGFP at MOI of 50 at 13 hpi. Host cell membranes were stained with CellMask deep red (magenta). Scale bars represent 5 µm. Images representative of at least three biological replicates. (**B**) Example of *B. thailandensis* assembling T6SS-1 inside of a dextran-labelled phagosome in A549 cells. Host cells were pre-treated with 0.25 mg/mL of fluorescent dextran for 18 h (magenta). Images were taken at 1:30 hpi. Blue arrows indicate T6SS-1 assembly events. Scale bar represents 2 µm. Images representative of at least 3 biological replicates. (**C**) Time-lapse microscopy of A549 cells infected with *B. thailandensis* expressing TssB-1-msfGFP and TssB-5-mCherry2 at MOI of 500 at 3 hpi. Host cells were pre-treated with 0.25 mg/mL of fluorescent dextran for 18 h (magenta). Blue arrows indicate a bacteria assembling T6SS-1 and not expressing T6SS-5. Scale bar represents 2 µm. Images representative of three biological replicates.

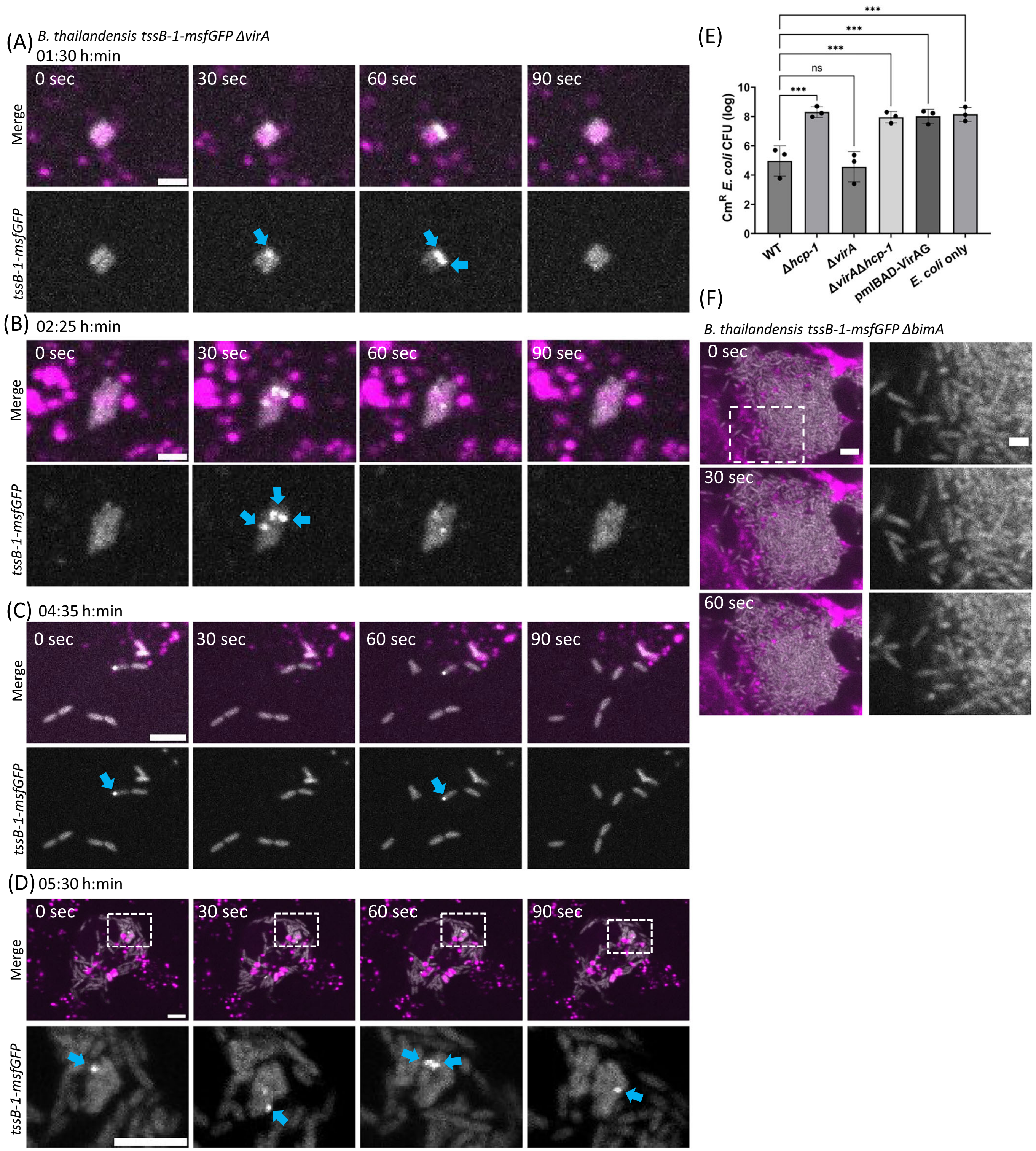
(A) B. thailandensis tssB-1-msfGFP ΔvirA
01:30 h:min
0 sec
30 sec
60 sec
90 sec
Merge
tssB-1-msfGFP
(B) 02:25 h:min
(C) 04:35 h:min
(D) 05:30 h:min
(E)
***
ns
Cm^R E. coli CFU (log)
WT
Δhcp-1
ΔvirA
ΔvirAΔhcp-1
pmlBAD-VirAG
E. coli only
(F) B. thailandensis tssB-1-msfGFP ΔbimA

Figure EV5. Intracellular T6SS-1 dynamics and bacterial competition assay.

Time-lapse microscopy of A549 cells infected with *B. thailandensis ΔvirA*, TssB-1-msfGFP at MOI of 50 at (**A**) 1:30 hpi, (**B**) 2:25 hpi, (**C**) 4:35 hpi, and (**D**) 5:30 hpi. A549 cells were pre-treated with 0.25 mg/mL of fluorescent dextran (magenta) to identify bacteria inside phagosomes. Blue arrows indicate T6SS-1 assembly events. Scale bars represent 2 µm for (**A**, **B**) and 5 µm for (**C**, **D**). Images representative of at least three biological replicates. (**E**) Recovery of viable *E. coli* prey after competition with *B. thailandensis* strains. *E. coli* prey harbouring a plasmid conferring chloramphenicol resistance was co-cultured with *B. thailandensis* strains at 1:1 ratio on LB agar plates for 5 h at 30 degrees. The co-cultures were harvested and serially diluted and spotted on LB agar plates containing 30 µg/mL chloramphenicol. Colony forming units (CFU) of *E. coli* was plotted on a logarithmic scale. Ordinary one-way ANOVA with Dunnett's multiple comparisons test was performed (****P* < 0.001, ns = not significant). Adjusted *P* values were as follows: [group WT vs. Δ*hcp-1*], $P = 3.0 \times 10^{-4}$; [group WT vs. Δ*virA*], $P = 9.260 \times 10^{-1}$; [group WT vs. Δ*virA*Δ*hcp-1*], $P = 8.0 \times 10^{-4}$; [group WT vs. pmlBAD-VirAG], $P = 7.0 \times 10^{-4}$; [group WT vs. *E. coli*], $P = 5.0 \times 10^{-4}$. Error bars represent mean ± SD. (**F**) Time-lapse microscopy of A549 cells infected with *B. thailandensis* Δ*bimA*, TssB-1-msfGFP at MOI of 50 at 14 hpi. Host cell membranes were stained with CellMask deep red (magenta). Scale bars represent 5 µm. Images representative of three biological replicates.

© The Author(s)

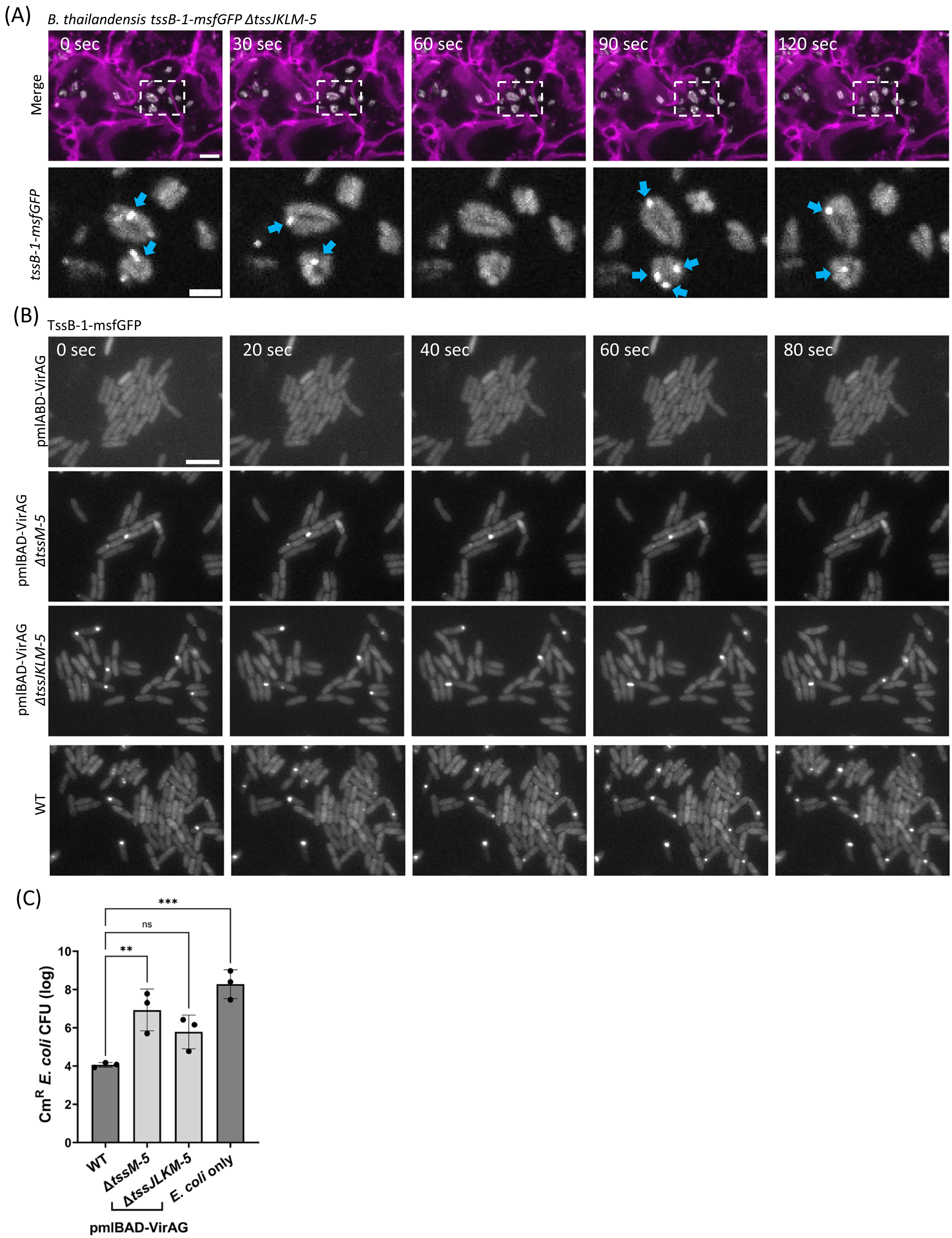
(A)
B. thailandensis tssB-1-msfGFP ΔtssJKLM-5
0 sec
30 sec
60 sec
90 sec
120 sec
Merge
tssB-1-msfGFP
(B)
TssB-1-msfGFP
0 sec
20 sec
40 sec
60 sec
80 sec
pmlABD-VirAG
pmlBAD-VirAG ΔtssM-5
pmlBAD-VirAG ΔtssJKLM-5
WT
(C)
***
ns
**
Cm^R E. coli CFU (log)
0
2
4
6
8
10
WT
ΔtssM-5
ΔtssJLKM-5
E. coli only
pmlBAD-VirAG

 © The Author(s)

Figure EV6. T6SS-1 assemblies are restored by deletion of T6SS-5.

(**A**) Time-lapse images of A549 cells infected with *B. thailandensis* Δ*tssJLKM-5* expressing TssB-1-msfGFP (grey) at MOI of 50 at 15:40 hpi. Cells were stained with CellMask deep red (magenta) and images were acquired at 30 s intervals. Blue arrows indicate T6SS-1 assemblies. Scale bars represent 5 μm in the overview image and 2 μm in the magnified image. Images representative of at least three biological replicates. (**B**) Time-lapse microscopy of *B. thailandensis* strains on agar pads. Top row: *B. thailandensis* harbouring the arabinose-inducible plasmid pmlBAD-VirAG. Second row: *B. thailandensis* Δ*tssM-5* harbouring pmlBAD-VirAG. Third row: *B. thailandensis* Δ*tssJLKM-5* harbouring pmlBAD-VirAG. Bottom row: WT *B. thailandensis*. Overnight bacterial cultures were subcultured to mid-log phase with 0.4% L-arabinose in LB and imaged on LB agar pads. Images were acquired at 10 s intervals. Scale bar represents 5 μm. Images representative of at least three biological replicates. (**C**) Recovery of viable *E. coli* prey after competition with *B. thailandensis* strains. *E. coli* prey harbouring a plasmid conferring chloramphenicol resistance was co-cultured with *B. thailandensis* strains at 1:1 ratio on LB agar plates for 5 h at 30 degrees. The co-cultures were harvested and serially diluted and spotted on LB agar plates containing 30 μg/mL chloramphenicol. Colony forming units (CFU) of *E. coli* was plotted on a logarithmic scale. Ordinary one-way ANOVA with Dunnett's multiple comparisons test was performed ($n = 3$, $^{**}P < 0.01$, $^{***}P < 0.001$, ns = not significant). Adjusted *P* values were as follows: [group WT vs. Δ*tssM-5*, pmlBAD-VirAG], $P = 6.0 \times 10^{-3}$; [group WT vs. Δ*tssJLKM-5*, pmlBAD-VirAG], $P = 7.14 \times 10^{-2}$; [group WT vs. *E. coli*], $P = 5.0 \times 10^{-4}$. Error bars represent mean ± SD.

© The Author(s)
