# Supplementary material for: Regulation of Type VI Secretion Systems of Burkholderia during transition to intracellular lifestyle
Source: EMBO Rep. 2026 Sep 2;27(19):5659–93. doi: 10.1038/s44319-026-00905-2 (PMC13650128; doi:10.1038/s44319-026-00905-2)
Supplement: Supplementary file 3 [file 44319_2026_905_MOESM2_ESM.pdf]

EMBO reports

Regulation of Type VI Secretion Systems of Burkholderia during transition to intracellular lifestyle

Hoi Ching Cheung, Daria Wüst, Miro Plum, Patricia Reist Iscar, and Marek Basler

Corresponding author(s): Marek Basler (marek.basler@unibas.ch)

Review Timeline:

Submission Date:	17th Feb 26
Editorial Decision:	24th Mar 26
Revision Received:	23rd Jun 26
Editorial Decision:	28th Jul 26
Revision Received:	31st Jul 26
Accepted:	12th Aug 26

Editor: Achim Breiling

Transaction Report:

(Note: With the exception of the correction of typographical or spelling errors that could be a source of ambiguity, letters and reports are not edited. Depending on transfer agreements, referee reports obtained elsewhere may or may not be included in this compilation. Referee reports are anonymous unless the Referee chooses to sign their reports.)

Dear Dr. Basler,

Thank you for the transfer of your manuscript to EMBO reports. I have now received reports from the three referees that were asked to evaluate your study, which can be found at the end of this email. As you will see, the referees have several comments, concerns, and suggestions, indicating that a major revision of the manuscript is necessary to allow publication of the study in EMBO reports. As the reports are below, and all points need to be addressed, I will not detail them here.

Given the constructive referee comments, I would like to invite you to revise your manuscript with the understanding that the concerns of the referees must be addressed in the revised manuscript and in a detailed point-by-point response. Acceptance of your manuscript will depend on a positive outcome of a second round of review. It is EMBO reports policy to allow a single round of revision only and acceptance of the manuscript will therefore depend on the completeness of your responses included in the next, final version of the manuscript.

Revised manuscripts should be submitted within three months of a request for revision. Please contact me to discuss the revision (also by video chat) if you have questions or comments regarding the revision, or should you need additional time.

When submitting your revised manuscript, please also carefully review the instructions that follow below.

PLEASE NOTE THAT upon resubmission revised manuscripts are subjected to an initial quality control prior to exposition to re-review. Upon failure in the initial quality control, the manuscripts are sent back to the authors, which may lead to delays. Frequent reasons for such a failure are the lack of the data availability section (please see below) and the presence of statistics based on n=2 (the authors are then asked to present scatter plots or provide more data points).

When submitting your revised manuscript, we will require:

1) a .docx formatted version of the final manuscript text (including legends for main figures, EV figures and tables), but without the figures included. Figure legends should be compiled at the end of the manuscript text.

2) individual production quality figure files as .eps, .tif, .jpg (one file per figure), of main figures and EV figures. Please upload these as separate, individual files upon re-submission.

The Expanded View format, which will be displayed in the main HTML of the paper in a collapsible format, has replaced the Supplementary information. You can submit up to 6 images as Expanded View. Please follow the nomenclature Figure EV1, Figure EV2 etc. The figure legend for these should be included in the main manuscript document file in a section called Expanded View Figure Legends after the main Figure Legends section. Additional Supplementary material should be supplied as a single pdf file labeled Appendix. The Appendix should have page numbers and needs to include a table of content on the first page (with page numbers) and legends for all content. Please follow the nomenclature Appendix Figure Sx, Appendix Table Sx etc. throughout the text, and also label the figures and tables according to this nomenclature.

The nomenclature for movie files is Movie EVx. Place change this in all places and add callouts for each file in the manuscript text. Please also provide legends for each movie. Each legend needs to be provided as separate text file and then uploaded, ZIPped together with its corresponding movie. Do not provide movie legends in the main text file.

For more details, please refer to our guide to authors:
https://link.springer.com/journal/44319/submission-guidelines#cms-Revised-submissions

Please consult our guide for figure preparation:
https://media.springernature.com/original/springer-cms/rest/v1/content/27825798/data/v1

3) a .docx formatted letter INCLUDING the reviewers' reports and your detailed point-by-point responses to their comments. As part of the EMBO Press transparent editorial process, the point-by-point response is part of the Review Process File (RPF), which will be published alongside your paper.

4) a complete author checklist, which you can download from our author guidelines (https://link.springer.com/journal/44319/submission-guidelines#cms-Revised-submissions). Please insert page numbers in the checklist to indicate where the requested information can be found in the manuscript. The completed author checklist will also be part of the RPF.

Please also follow our guidelines for the use of living organisms, and the respective reporting guidelines:
https://link.springer.com/partners/embo-press/editorial-policies#Animal%20and%20human%20studies

5) that primary datasets produced in this study (e.g. RNA-seq, ChIP-seq, structural and array data) are deposited in an

appropriate public database. If no primary datasets have been deposited, please also state this in a dedicated section (e.g. 'No primary datasets have been generated and deposited'), see below.

See also: https://link.springer.com/partners/embo-press/editorial-policies#Data%20deposition

Please remember to provide a reviewer password if the datasets are not yet public.

The accession numbers and database should be listed in a formal "Data Availability" section that follows the model below. This is now mandatory (like the COI statement). Please note that the Data Availability Section is restricted to new primary data that are part of this study. This section is mandatory. As indicated above, if no primary datasets have been deposited, please state this in this section

Data availability

The datasets produced in this study are available in the following databases:

- RNA-Seq data: Gene Expression Omnibus GSE46843 (https://www.ncbi.nlm.nih.gov/geo/query/acc.cgi?acc=GSE46843)
- [data type]: [name of the resource] [accession number/identifier/doi] ([URL or identifiers.org/DATABASE:ACCESSION])

*** Note - All links should resolve to a page where the data can be accessed. ***

See also: https://link.springer.com/partners/embo-press/editorial-policies#Data%20availability%20statement

Moreover, I have these editorial requests:

6) We now request the publication of original source data with the aim of making primary data more accessible and transparent to the reader. You will receive a separate email with instructions for providing source data with your revised manuscript, including information how to upload and organize the files.

7) Our journal encourages inclusion of *data citations in the reference list* to directly cite datasets that were re-used and obtained from public databases. Data citations in the article text are distinct from normal bibliographical citations and should directly link to the database records from which the data can be accessed. In the main text, data citations are formatted as follows: "Data ref: Smith et al, 2001" or "Data ref: NCBI Sequence Read Archive PRJNA342805, 2017". In the Reference list, data citations must be labeled with "[DATASET]". A data reference must provide the database name, accession number/identifiers and a resolvable link to the landing page from which the data can be accessed at the end of the reference. Further instructions are available at: https://link.springer.com/journal/44319/submission-guidelines#cms-Reference-guidelines

8) Regarding data quantification and statistics, please make sure that the number "n" for how many independent experiments were performed, their nature (biological versus technical replicates), the bars and error bars (e.g. SEM, SD) and the test used to calculate p-values is indicated in the respective figure legends (also for EV and Appendix figures). Please also check that all the p-values are explained in the legend, and that these fit to those shown in the figure. Please provide statistical testing where applicable. Please avoid the phrase 'independent experiment' but clearly state if these were biological or technical replicates. Please also indicate (e.g. with n.s.) if testing was performed, but the differences are not significant. In case n=2, please show the data as separate datapoints without error bars and statistics. See also:
https://link.springer.com/journal/44319/submission-guidelines#cms-Figure-and-data-presentation

If n<5, please show single datapoints for diagrams.

9) Please add scale bars of similar style and thickness to microscopic images, using clearly visible black or white bars (depending on the background). Please place these in the lower right corner of the images themselves. Please do not write on or near the bars in the image but define the size in the respective figure legend.

10) Please also note our reference format:
https://link.springer.com/journal/44319/submission-guidelines#cms-Reference-guidelines

11) We updated our journal's competing interests policy in January 2022 and request authors to consider both actual and perceived competing interests. Please name this section 'Disclosure and Competing Interests Statement' and put it after the Acknowledgements section.
See: https://link.springer.com/partners/embo-press/editorial-policies#Competing%20interest%20disclosures

12) We now use CRediT to specify the contributions of each author in the journal submission system. CRediT replaces the author contribution section. Please use the free text box to provide more detailed descriptions and do NOT provide your final manuscript text file with an author contributions section. See also our guide to authors (section 'Author contributions'):
https://link.springer.com/journal/44319/submission-guidelines#cms-Revised-submissions

13) All Materials and Methods need to be described in the main text using our 'Structured Methods' format, which is required for all research articles. According to this format, the Methods section should include a Reagents and Tools Table (listing key reagents, experimental models, software, and relevant equipment and including their sources and relevant identifiers), uploaded as separate file, and a Methods section in which we encourage the authors to describe their methods using a step-by-step protocol format with bullet points, to facilitate the adoption of the methodologies across labs. More information on how to adhere to this format as well as downloadable templates (.doc) for the Reagents and Tools Table can be found in our author guidelines (section 'Structured Methods'):

https://link.springer.com/journal/44319/submission-guidelines#cms-Manuscript-organisation-and-formatting

Please add all primer or antibody information directly to the Reagents and Tools table.

14) Please add up to five keywords and order the manuscript sections like this, using only these names:
Title page - Abstract (max. 175 words) - Keywords (max. 5) - Introduction - Results - Discussion - Methods - Data availability section - Acknowledgements (please include here all the funding information) - Disclosure and Competing Interests Statement - References - Figure legends - Expanded View Figure legends

15) Please make sure that all the funding information is also entered into the online submission system and that it is complete and similar to the one in the acknowledgement section of the manuscript text file.

We would also welcome the submission of cover suggestions, or motifs to be used by our Graphics Illustrator in designing a cover.

As part of the EMBO publication's Transparent Editorial Process, EMBO reports publishes online a Review Process File (RPF) to accompany accepted manuscripts. This File will be published in conjunction with your paper and will include the referee reports, your point-by-point response and all pertinent correspondence relating to the manuscript.

You are able to opt out of this by letting the editorial office know (emboreports@embo.org). If you do opt out, the Review Process File link will point to the following statement: "No Review Process File is available with this article, as the authors have chosen not to make the review process public in this case."

I look forward to seeing a revised form of your manuscript when it is ready.

Please use this link to submit your revision: https://embor.msubmit.net/cgi-bin/main.plex

Yours sincerely,

Achim Breiling
Senior Editor
EMBO Reports

Referee #1:

In this manuscript, fluorescence imaging was used to investigate the regulation of the different T6SS of B.thailandensis. Reporter strains were constructed, and they were used in conjunction with fluorescent reporters of host cell compartments and membranes. Furthermore, bacterial mutants of the VirAG system were investigated and their impact on the expression of the bacterial T6SS, always in the context of cell infection. Finally, the impact on infected host cells was analyzed focusing on mitochondrial damage and apoptotic cell death. Through the presented work the authors propose the key regulatory role of VirAG to switch between T6SS-1 and T6SS-5.

This is very interesting discovery and has an important potential as the differential regulation of the T6SS in these bacteria have remained poorly understood before. Despite my excitement about this and the prospect of the work, I had some major issues as described below with the compartmental labeling strategy used by the authors, with experimental design (especially to interpret whether bacteria are in the cytosol), and with the quantification based on dot formation of their fluorescent fusion proteins interpreted as assembled T6SS. Due to this and the lack of controls, I could not interpret all data presented by the authors.

Major points:
The authors check expression of the different T6SS during intracellular infection steps. They show how the strains were generated, but if I looked correctly, T6SS-2, 4 and 6 (to some extent) were not expressed under any of the conditions tested (in vitro and in cellulo). Such a negative results could be real or they could be due to a technical issue. Therefore, the authors should also look at the transcriptional level what is going on, also to compare their work with the published literature (citation 48). This would require control experiments.

The phagosomal compartment has not been labeled with a marker protein by the authors in their timelapse experiments. They use dextran instead as proxy, and it is implicated that this compartment is the "phagosome". However, dextran-positive compartments label many compartments, especially after the long period of labeling used here. The authors need to use specific markers for the labeling of phagolysosomal compartment similar to Rab5/Rab7- why did they not use LAMP1-mApple as for the Rabs?

The protocol used for the Cell Mask stain before timelapse imaging seems not very specific to me- it is possible that bacteria negative for Cell Mask are still within a membrane-bound compartment, I suspect that this dye does not label endomembranes comprehensively. I think a quantification is needed on the global population of intracellular bacteria- are they all positive for cell mask early during infection?
The authors claim that bafilomycin treatment affected T6S-5 expression, however only a small field with some bacteria is shown. It would be good to have complementary data with representative numbers of bacteria- FACS data would be helpful here.

Line 124: "Notably, the T6SS-5 assemblies did not disrupt the phagosomal membrane"- I am not absolutely sure if the authors can deduce from the fluorescence dotty staining that complexes assemble. It could also be that proteins aggregate due to the fluorescent protein. This happens often with such fusion proteins. I suggest that the authors are more careful with the assumption of "assembly". Later, the authors describe data on the non-assembly of T6SS-1 (line 186ff). This part is more convincing as there is a lack of dots, nevertheless in case dots form, they could simply be an artifact of the expression of the fusion protein. I find that either biochemical or structural data is needed to make a point that each fluorescent dot is actually an assembled T6SS. Without such controls there is a risk of misinterpretation.

Line 175-177: How representative is this data? I find that the text is unclear how significant the data is.
I find that the dextran assay for phagosomal rupture is problematic for this particular study: the authors load cells with dextran, and they add bacteria afterwards. The bacterial compartment will only become dextran-positive in case it fuses with the previously added dextran-positive compartments. It is unclear to me at which stage and to which extent this happens, for example it could happen before or after the steps affected by bafilomycin-treatment. The suggested control in Fig2F does not allow conclusions on this. Therefore, I have problems with the interpretation of the results from the experiments relying on dextran and bafilomycin in this work. I am unable to interpret the results on this without additional controls.

Minor points:
Line 107- there is an issue with the sentence..."while"

I find the term "cytosolic mCherry" easily confusing (as there is also the cytosol of the host cell)- I guess the authors wish to say that the protein was simply expressed in bacteria

----
Referee #2:

Cheung et al. investigated the coordination of multiple T6SS in B. thailandensis as it adapts to an intracellular lifestyle, revealing a distinct regulatory switch controlled by the VirAG two-component system. The authors demonstrate that while the antibacterial T6SS-1 is initially active, its assembly is progressively inhibited during phagosomal maturation as the bacterium transitions to expressing the anti-eukaryotic T6SS-5. Failure to inhibit T6SS-1 results in significant mitochondrial fragmentation and premature host cell apoptosis, which would otherwise destroy the bacterium's replicative niche.

This high-quality study provides significant insights into the regulation and assembly of the T6SS in Burkholderia, ensuring that the pathogen effectively balances interbacterial competition with host cell survival. However, there is some room for improvement, especially in one specific experiment that would help clarify the regulatory mechanism governed by VirAG, and some controls should be added to strengthen the conclusions. In addition, several points for discussion should be expanded, and minor textual corrections should be made to improve clarity and flow.

Major Comments:
This study suggests that the VirAG two-component system regulates a post-translational process, specifically the assembly of T6SS-1. As these systems typically regulate gene expression, this effect is likely to be indirect. To distinguish between possible regulatory pathways, the authors should explore whether T6SS-5 components themselves (upregulated by VirAG) are blocking T6SS-1 assembly at the protein-protein level. This could be achieved by studying the inhibition of T6SS-1 assembly by VirAG in a T6SS-5 whole-cluster mutant. If T6SS-1 assembly is no longer inhibited under these conditions, it confirms that T6SS-5 components block T6SS-1 assembly. If inhibition persists, the regulatory pathway might involve a different intermediate. The current title of this section (L205) could be misleading without this clarification.

The manuscript demonstrates that BTH_I2698 lipase impacts mitochondrial membranes; it would be good to demonstrate that it does not impact eukaryotic cell membranes, in addition to mitochondrial membranes.
The authors discussed that fagosomal maturation is key for T6SS-5 activation (L177-178). It would be beneficial to determine

whether T6SS-5 can be activated in vitro by factors such as acidification or redox changes during growth in media.

In the experiment described in L261-265, the controls for mitochondria without bacterial infection and in the presence of CCCP are currently missing.

Discussion Points to be expanded:
The authors should include what is currently known about T6SS-1 regulation and the potential indirect nature of post-translational control.

Is BTH_I2698 lipase the only T6SS-1 effector? The authors should discuss other T6SS-1 known effectors (antibacterial) and whether any other could have a transkingdom effect, such as lipases.
The authors observed a fraction of mitochondria fragmenting in a T6SS-1 independent manner (L264 and L272); this phenomenon should be discussed.

The authors should clarify what happens to the remaining percentage of T6SS-expressing bacteria not covered by the 2%, 16.5%, and 29% (L156-158).

Minor Points:
There are several instances in which figures are cited out of order. For example, Figure 1F appears before Figure 1E (L122, L154), Figure 2F appears before Figures 2D and 2E, and Figure 1G appears before Figure 1E (L124, L143).

Dextran is mentioned in Fig 1G, but its use for labeling vacuoles is not explained until later (L140/L164); this should be introduced earlier.

Provide a brief explanation of what the reporters mean for readers unfamiliar with intracellular pathogens and refer to Fig 2A, which is currently missing from the main text (L150-151).

The explanation regarding how expression vs. assembly is visualised via microscopy (L187-188) should be moved to the beginning of the Results section to aid reader interpretation.

L107: Delete "and" after "while".

L122: Use "Fig 1F" instead of "Figure 1F" for consistency (other cases through the text).

L146: This sentence requires interpretation of the results.

Referee #3:

This study shows that Burkholderia thailandensis dynamically regulates its Type VI secretion systems (T6SS) during infection. Evidence is presented that upon entry into host cells, the bacterium switches from the antibacterial T6SS-1 to the anti-eukaryotic T6SS-5 during phagosomal maturation. This transition is controlled by the two-component system VirAG, which represses T6SS-1 assembly while activating T6SS-5. The authors further demonstrate that T6SS-1 activity in host cells induces mitochondrial fragmentation and apoptosis, suggesting that its downregulation is necessary to preserve the intracellular replicative niche.

The major strength of this study is the detailed and elegant microscopic analysis of the timing of induction of the T6SS-1 and T6SS-5 systems. The authors observed T6SS-5 assemblies inside late phagosomes, while T6SS-1 is expressed but not assembled during infection. A major weakness of the paper is that the stimuli affecting VirAG activity have not been unambiguously identified. As a result, the advance in our understanding of the dynamic regulation of these systems remains limited.

Importantly, the results presented appear to be in contrast with previous work showing that T6SS-5 is activated in the host cytosol but not in phagosomes. In that study, the signal required for VirAG-dependent activation of T6SS-5 was identified as low-molecular-weight thiols, particularly glutathione, which are present in the cytoplasm but absent from phagosomes. Although the authors cite this publication, they neither discuss this apparent discrepancy nor attempt to resolve it. Based on experiments in which vacuolar acidification was inhibited by bafilomycin A1, the authors suggest that acidification and redox changes accompanying phagosome maturation may initiate T6SS-5 activation. However, additional in vitro experiments would be required to clarify this discrepancy and to unambiguously identify the signal responsible for T6SS-5 activation in phagosomes.

It is also somewhat surprising that the authors did not attempt to characterize the regulatory cascade downstream of VirAG, which could provide mechanistic insight into the differential regulation of the T6SS-1 and T6SS-5 systems.

Finally, the finding that T6SS-1 activity in host cells induces mitochondrial fragmentation and apoptosis is intriguing, but it appears somewhat artificial, as this phenotype is observed only in a virA mutant. It would be helpful if the authors could discuss under what biological circumstances this effect might be relevant. For example, have spontaneous virA mutants been identified?

Referee #1:

In this manuscript, fluorescence imaging was used to investigate the regulation of the different T6SS of B.thailandensis. Reporter strains were constructed, and they were used in conjunction with fluorescent reporters of host cell compartments and membranes. Furthermore, bacterial mutants of the VirAG system were investigated and their impact on the expression of the bacterial T6SS, always in the context of cell infection. Finally, the impact on infected host cells was analyzed focusing on mitochondrial damage and apoptotic cell death. Through the presented work the authors propose the key regulatory role of VirAG to switch between T6SS-1 and T6SS-5.

This is very interesting discovery and has an important potential as the differential regulation of the T6SS in these bacteria have remained poorly understood before. Despite my excitement about this and the prospect of the work, I had some major issues as described below with the compartmental labeling strategy used by the authors, with experimental design (especially to interpret whether bacteria are in the cytosol), and with the quantification based on dot formation of their fluorescent fusion proteins interpreted as assembled T6SS. Due to this and the lack of controls, I could not interpret all data presented by the authors.

Major points:
The authors check expression of the different T6SS during intracellular infection steps. They show how the strains were generated, but if I looked correctly, T6SS-2, 4 and 6 (to some extent) were not expressed under any of the conditions tested (in vitro and in cellulo). Such a negative results could be real or they could be due to a technical issue. Therefore, the authors should also look at the transcriptional level what is going on, also to compare their work with the published literature (citation 48). This would require control experiments.

We thank the reviewer for pointing this out. However, constructing fluorescent fusion proteins of T6SS sheath TssB is an established method, all fusions were created by the same method, and the final constructs were sequenced. In the study by (Heacock-Kang et al., 2021), transcripts levels of T6SS-2, T6SS-4, and T6SS-6 of *B. pseudomallei* were compared between infection and liquid culture conditions , however, the authors detected only a limited subset of T6SS genes (1–3 components). This suggests partial expression rather than a full system-wide activation. We think that trying to understand our failure to detect expression under our experimental conditions could be tedious while having no impact on improving our study. We would be willing to remove the description those negative results if this would be requested.

The phagosomal compartment has not been labeled with a marker protein by the authors in their timelapse experiments. They use dextran instead as proxy, and it is implicated that this compartment is the "phagosome". However, dextran-positive compartments label many

compartments, especially after the long period of labeling used here. The authors need to use specific markers for the labeling of phagolysosomal compartment similar to Rab5/Rab7- why did they not use LAMP1-mApple as for the Rabs?

Unfortunately, we had no access to a suitable cell line to directly visualize LAMP1 in live cell imaging experiments. However, we have independently assessed vacuolar localization using LAMP1 immunostaining.

The protocol used for the Cell Mask stain before timelapse imaging seems not very specific to me- it is possible that bacteria negative for Cell Mask are still within a membrane-bound compartment, I suspect that this dye does not label endomembranes comprehensively. I think a quantification is needed on the global population of intracellular bacteria- are they all positive for cell mask early during infection?

It is possible that bacteria within a membrane-bound compartment could be negative for CellMask staining. In our time-lapse videos, there is some heterogeneity in timing when bacteria are positive for CellMask. Since CellMask is a lipophilic dye that labels membranes unspecifically, CM-stained plasma membranes will be endocytically taken up and it depends on the time between staining and start of imaging.

Our intention with the CellMask staining was to demonstrate that a subset of bacteria resides within membrane-bound compartments during T6SS-5 expression, not necessarily to compare fractions of membrane-bound and cytosolic bacteria. Therefore, any bacteria that colocalize with CM signal are correctly identified, however, it is true that even more bacteria could be expressing T6SS-5 while still being membrane-bound. These observations motivated the subsequent dextran-labelling experiments to confirm that bacteria reside in phagosomes.

The authors claim that bafilomycin treatment affected T6S-5 expression, however only a small field with some bacteria is shown. It would be good to have complementary data with representative numbers of bacteria- FACS data would be helpful here.

We thank the reviewer for this suggestion. Indeed, we have included FACS data, shown in Figure 2G and EV3C (Lines 198-202) that confirm our previous observations.

Line 124: "Notably, the T6SS-5 assemblies did not disrupt the phagosomal membrane"- I am not absolutely sure if the authors can deduce from the fluorescence dotty staining that complexes assemble. It could also be that proteins aggregate due to the fluorescent protein. This happens often with such fusion proteins. I suggest that the authors are more careful with the assumption of "assembly". Later, the authors describe data on the non-assembly of T6SS-1 (line 186ff). This part is more convincing as there is a lack of dots, nevertheless in case dots form, they could simply be an artifact of the expression of the fusion protein. I find that

either biochemical or structural data is needed to make a point that each fluorescent dot is actually an assembled T6SS. Without such controls there is a risk of misinterpretation.

When we classify a structure as proper T6SS assembly, we only consider fluorescent foci that are dynamic and undergo assembly and disassembly over time (about 30s of assembly and up to a minute for disassembly). This excludes foci that would be simple aggregates of TssB fusion protein. The functionality of the TssB-5 fusion protein was previously validated in our earlier publication (Plum et al., 2024), and the morphology and dynamics of the foci observed here are consistent with those previously reported. Therefore, we believe that such live-cell imaging and scoring sheath dynamics is the most direct and sensitive way to confirm T6SS activity at the single cell level and with high temporal resolution. We are not aware of any other method independent that could achieve the same.

Line 175-177: How representative is this data? I find that the text is unclear how significant the data is.

The line referred to here is "The bacteria were able to replicate inside of dextran labelled vacuoles but were unable to escape from the phagosome". We have now added a quantification and showed 100% of bacteria in BAF-treated cells are localized to dextran labelled vacuoles (Figure 3E, lines 220-222).

I find that the dextran assay for phagosomal rupture is problematic for this particular study: the authors load cells with dextran, and they add bacteria afterwards. The bacterial compartment will only become dextran-positive in case it fuses with the previously added dextran-positive compartments. It is unclear to me at which stage and to which extent this happens, for example it could happen before or after the steps affected by bafilomycin-treatment. The suggested control in Fig2F does not allow conclusions on this. Therefore, I have problems with the interpretation of the results from the experiments relying on dextran and bafilomycin in this work. I am unable to interpret the results on this without additional controls.

Dextran was preloaded into the endolysosomal system by incubating cells with dextran for 18 h prior to infection, and excess dextran was removed immediately before bacterial addition. Under normal conditions, bacteria-containing phagosomes mature and fuse with dextran-labelled endolysosomal compartments. Consistent with previous literature and our own observations showing that *Burkholderia* localizes to Rab5- and Rab7-positive vacuoles(Hu et al., 2019), these data support normal progression through the early stages of phagosomal maturation. Importantly, Bafilomycin A1 primarily inhibits lysosomal acidification and late phagosome–lysosome fusion(Mauvezin & Neufeld, 2015), and is therefore not expected to interfere with earlier maturation events.

We have performed a new experiment of infecting Galectin-3-GFP cells with *B. thailandensis*, either treated with bafilomycin A1 (BAF) or DMSO. Galectin-3 (Gal-3) binds to ruptured

membrane vacuoles. We show that there is no Gal-3 colocalization with bacterial-containing vacuoles in the BAF-treated cells (Figure 3). This further supports the interpretation that dextran labelled phagosomes are not affected with BAF treatment, and that the phagosomal membrane remains intact.

Minor points:
Line 107- there is an issue with the sentence..."while"

Thank you for pointing it out. We have now corrected the sentence.

I find the term "cytosolic mCherry" easily confusing (as there is also the cytosol of the host cell)- I guess the authors wish to say that the protein was simply expressed in bacteria

We now rephrased the sentence to make it clear that this is cytosolic mCherry in the bacteria.

Referee #2:

Cheung et al. investigated the coordination of multiple T6SS in B. thailandensis as it adapts to an intracellular lifestyle, revealing a distinct regulatory switch controlled by the VirAG two-component system. The authors demonstrate that while the antibacterial T6SS-1 is initially active, its assembly is progressively inhibited during phagosomal maturation as the bacterium transitions to expressing the anti-eukaryotic T6SS-5. Failure to inhibit T6SS-1 results in significant mitochondrial fragmentation and premature host cell apoptosis, which would otherwise destroy the bacterium's replicative niche.

This high-quality study provides significant insights into the regulation and assembly of the T6SS in Burkholderia, ensuring that the pathogen effectively balances interbacterial competition with host cell survival. However, there is some room for improvement, especially in one specific experiment that would help clarify the regulatory mechanism governed by VirAG, and some controls should be added to strengthen the conclusions. In addition, several points for discussion should be expanded, and minor textual corrections should be made to improve clarity and flow.

Major Comments:
This study suggests that the VirAG two-component system regulates a post-translational process, specifically the assembly of T6SS-1. As these systems typically regulate gene expression, this effect is likely to be indirect. To distinguish between possible regulatory pathways, the authors should explore whether T6SS-5 components themselves (upregulated by VirAG) are blocking T6SS-1 assembly at the protein-protein level. This could be achieved by studying the inhibition of T6SS-1 assembly by VirAG in a T6SS-5 whole-cluster mutant. If

T6SS-1 assembly is no longer inhibited under these conditions, it confirms that T6SS-5 components block T6SS-1 assembly. If inhibition persists, the regulatory pathway might involve a different intermediate. The current title of this section (L205) could be misleading without this clarification.

Thank you for the suggestion. Indeed, we now show that deletion of *tssM-5* and *tssJLK-5* (T6SS-5 membrane complex and baseplate components) restores T6SS-1 assembly during infection. Deletion of these T6SS-5 components also restores T6SS-1 assembly on agar pads when VirAG is overexpressed. This new data is included in Figure 6 and EV6, description of the results is in lines 292-313.

The manuscript demonstrates that BTH_I2698 lipase impacts mitochondrial membranes; it would be good to demonstrate that it does not impact eukaryotic cell membranes, in addition to mitochondrial membranes.

We thank the reviewer for this suggestion. To directly assess whether the eukaryotic plasma membrane is affected, we performed a propidium iodide (PI) uptake assay. As a positive control for membrane permeabilization, uninfected cells were treated with ethanol, which resulted in robust nuclear PI staining. In contrast, cells infected with the Δ*virA* strain did not show detectable PI signal in the nuclei. These new data are now included in Figure 7F and Figure S3D (also referenced in lines 3357-368).

The authors discussed that fagosomal maturation is key for T6SS-5 activation (L177-178). It would be beneficial to determine whether T6SS-5 can be activated in vitro by factors such as acidification or redox changes during growth in media.

Despite numerous attempts, we failed to activate T6SS-5 expression *in vitro* by acidification or redox changes alone. One possible explanation is that expression of VirAG must first be upregulated prior to activation by environmental signals.

In the experiment described in L261-265, the controls for mitochondria without bacterial infection and in the presence of CCCP are currently missing.

We apologize if this was unclear in the original manuscript. The relevant data are shown in Figure 7A and the quantification for mitochondria fragmentation in Figure 7E (Lines 347-349).

Discussion Points to be expanded:
The authors should include what is currently known about T6SS-1 regulation and the potential indirect nature of post-translational control.

We have added a brief discussion of the current knowledge in lines 450-452. Relatively little is currently known about the regulation of T6SS-1. To date, studies have primarily focused on

the role of T6SS accessory genes and quorum sensing in regulating T6SS-1 activity. Our findings also now highlight a post-translational inhibition of T6SS-1 assemblies by T6SS-5 components.

Is BTH_I2698 lipase the only T6SS-1 effector? The authors should discuss other T6SS-1 known effectors (antibacterial) and whether any other could have a transkingdom effect, such as lipases.

We have added this in lines 475-488. We performed bioinformatics analysis of the predicted T6SS-1 effectors and found two effectors with lipase domains (BTH_I2698, BTH_I2701), and one effector belonging to the VasX toxin family (BTH_2691). These findings indeed suggest that the predicted T6SS-1 effectors could have transkingdom effects and will require further functional characterization in future studies, which is beyond the scope of the present work.

The authors observed a fraction of mitochondria fragmenting in a T6SS-1 independent manner (L264 and L272); this phenomenon should be discussed.

We have now included this discussion in lines 493-498. This is due to the fact the VirAG is a virulence regulator and deletion of *virA* impairs the intracellular fitness of *B. thailandensis*. We therefore speculate that the observed T6SS-1 independent mitochondria fragmentation may reflect effects of altered bacterial virulence. Nevertheless, we found a significant difference in mitochondrial fragmentation in the T6SS-1 assembling compared to the non-assembling strain, which allows us to conclude on the effect of T6SS-1 assemblies on host cell immune response.

The authors should clarify what happens to the remaining percentage of T6SS-expressing bacteria not covered by the 2%, 16.5%, and 29% (L156-158).

We have clarified this in lines 434-436. Markers such as Rab5, Rab7, and LAMP1 represent well-established components of the phagosomal maturation pathway, although additional markers also contribute to defining distinct vacuolar populations. In this study, we focused on these canonical markers as representative readouts of phagosomal progression. Bacteria not associated with these markers may therefore either reside in the host cytosol or occupy vacuolar compartments labelled by other unassessed markers.

Minor Points:
There are several instances in which figures are cited out of order. For example, Figure 1F appears before Figure 1E (L122, L154), Figure 2F appears before Figures 2D and 2E, and Figure 1G appears before Figure 1E (L124, L143).

Thank you for pointing this out. We have corrected the figure citation order.

Dextran is mentioned in Fig 1G, but its use for labeling vacuoles is not explained until later (L140/L164); this should be introduced earlier.

We have moved the text referring to Fig 1G (T6SS-5 firing inside of the phagosome) to after dextran has been introduced (Lines 159-161).

Provide a brief explanation of what the reporters mean for readers unfamiliar with intracellular pathogens and refer to Fig 2A, which is currently missing from the main text (L150-151).

We have added an explanation in lines 169-172.

The explanation regarding how expression vs. assembly is visualised via microscopy (L187-188) should be moved to the beginning of the Results section to aid reader interpretation.

We added an explanation at the end of the introduction (lines 86-89).

-

L107: Delete "and" after "while".
L122: Use "Fig 1F" instead of "Figure 1F" for consistency (other cases through the text).

Thank you for pointing these out. We have corrected the text.

L146: This sentence requires interpretation of the results.
The sentence the reviewer pointed out refers to "To elucidate at which step of the phagosomal maturation pathway is *B. thailandensis* expressing T6SS-5, we infected two HeLa cell lines stably expressing Rab5-mApple or Rab7-mApple, and stained with anti-LAMP1 (lysosome-associated membrane protein-1) antibody."

We have revised the sentence to read as follows (Lines 167-169)

"To assess phagosomal maturation during infection with B. thailandensis expressing T6SS-5, we infected two HeLa cell lines stably expressing Rab5-mApple or Rab7-mApple and stained for LAMP1 (lysosome-associated membrane protein-1) by immunofluorescence."

Referee #3:

This study shows that Burkholderia thailandensis dynamically regulates its Type VI secretion systems (T6SS) during infection. Evidence is presented that upon entry into host cells, the bacterium switches from the antibacterial T6SS-1 to the anti-eukaryotic T6SS-5 during phagosomal maturation. This transition is controlled by the two-component system VirAG, which represses T6SS-1 assembly while activating T6SS-5. The authors further demonstrate that T6SS-1 activity in host cells induces mitochondrial fragmentation and apoptosis, suggesting that its downregulation is necessary to preserve the intracellular replicative niche.

The major strength of this study is the detailed and elegant microscopic analysis of the timing of induction of the T6SS-1 and T6SS-5 systems. The authors observed T6SS-5 assemblies inside late phagosomes, while T6SS-1 is expressed but not assembled during infection. A major weakness of the paper is that the stimuli affecting VirAG activity have not been unambiguously identified. As a result, the advance in our understanding of the dynamic regulation of these systems remains limited.

Importantly, the results presented appear to be in contrast with previous work showing that T6SS-5 is activated in the host cytosol but not in phagosomes. In that study, the signal required for VirAG-dependent activation of T6SS-5 was identified as low-molecular-weight thiols, particularly glutathione, which are present in the cytoplasm but absent from phagosomes. Although the authors cite this publication, they neither discuss this apparent discrepancy nor attempt to resolve it.

Thank you for pointing this out. We have now expanded the section in discussion addressing this (Lines 416-422). We believe that the main difference is previous work focused at a later infection stage where most bacteria have escaped into the host cytosol (6 hpi) and we focused on bacteria within phagosomes (3 hpi). Nevertheless, both studies support the observation that T6SS-5 is expressed when *B. thailandensis* is intracellular.

Based on experiments in which vacuolar acidification was inhibited by bafilomycin A1, the authors suggest that acidification and redox changes accompanying phagosome maturation may initiate T6SS-5 activation. However, additional in vitro experiments would be required to clarify this discrepancy and to unambiguously identify the signal responsible for T6SS-5 activation in phagosomes.

Despite multiple attempts, T6SS-5 expression could not be induced *in vitro* by acidification or redox changes alone. One possible explanation is that expression of VirAG must first be upregulated prior to activation by environmental signals.

It is also somewhat surprising that the authors did not attempt to characterize the regulatory cascade downstream of VirAG, which could provide mechanistic insight into the differential regulation of the T6SS-1 and T6SS-5 systems.

Thank you for the suggestion. We have now included this in Figure 6 and S8. We deleted membrane complex and baseplate components of the T6SS-5 (TssJLKM-5) and restored T6SS-1 assemblies during infection and during VirAG overexpression experiments on agar pads. This implies a post-translational inhibition of T6SS-1 assembly by T6SS-5 components, contributing to the switch from T6SS-1 to T6SS-5 when the bacterium enters host cells.

Finally, the finding that T6SS-1 activity in host cells induces mitochondrial fragmentation and

apoptosis is intriguing, but it appears somewhat artificial, as this phenotype is observed only in a virA mutant. It would be helpful if the authors could discuss under what biological circumstances this effect might be relevant. For example, have spontaneous virA mutants been identified?

VirA knockouts are not occurring naturally. VirA knockout was indeed generated to artificially prevent inhibition of T6SS-1 during infection. By allowing T6SS-1 expression and assembly inside the host cell we could identify possible benefits for inhibition of T6SS-1 assembly during infection. We agree that this is somewhat artificial, however, we believe that the experiment still points to possible selective pressures that could have led to T6SS-1 inhibition. We phrase this carefully to not overinterpret our data.

Dear Dr. Basler

Thank you for the submission of your revised manuscript to EMBO reports. As my colleague Achim Breiling is currently out of office, I have temporarily taken over the handling of your manuscript. We have now received the full set of referee reports that is copied below.

As you will see, all referees are very positive about the study and recommend publication. Before I can accept the manuscript, I need you to address some minor points below:

- Please provide the main and EV figures as individual production quality Figure files (as .eps, .tif, .jpg).

- You have three Supplementary figures. These can remain part of one PDF but the nomenclature needs to change to Appendix Figure S#. This Appendix needs a title page stating "Appendix" and the manuscript title. It further needs a table of concent with page numbers and the figure legends are also part of the PDF.

- The Data availability section should only refer to datasets deposited in public repositories. Therefore, either remove that statement "Imaging dataset available on request" or deposit these data in a repository or as source data (preferred).

- The Fiji macro script .docx file would need a different nomenclature. It could either go to Github, into an Appendix (a PDF with a title page and a table of content), or into the methods section. Maybe the Appendix is a good place. Maybe ImageJ wiki is an alternative? I am happy to discuss this further.

- Regarding the Author Contributions, we use CRediT to specify the contributions of each author in the journal submission system. CRediT replaces the author contribution section, which therefore needs to be removed from the manuscript text. You can use the free text box in our system if you wish to provide more detailed descriptions. See also guide to authors https://www.embopress.org/page/journal/14693178/authorguide#authorshipguidelines.

- References: et al needs to be used after 10 author names; DOIs should only be used for preprints and datasets that have not been published yet.

- Please add callouts for Fig. 2AG, 3C. Supplementary Figure S5C, Fig S7A, and Figure S9A are called out but since there are only 3 Supplementary figures these callouts might refer to EV figures? We also need a callout for Movie EV4.

- Please update the 'Conflict of interest' paragraph to our new 'Disclosure and competing interests statement'. For more information see
https://link.springer.com/journal/44319/submission-guidelines#conflictsofinterest

- Summary should be Abstract.

- Materials and Methods should be Methods.

- Please provide the exact p values in the legends of figures 1C, 2E, F; 3D, E; 6C, D; 7E, 8C, EV6 C.

- Please provide information related to n in the legend of figure EV6 C.

- Please define the size of the scale bar for Supplementary figure 1A, B

- The Author Checklist refers to Table 1 in several places. Should this be the Reagents and Tools table? Please update this accordingly (the Checklist will be published as part of the Review Process File).

- Finally, EMBO Reports papers are accompanied online by
A) a short (1-2 sentences) summary of the findings and their significance,
B) 2-3 bullet points highlighting key results and
C) a schematic summary figure that provides a sketch of the major findings (not a data image).
Please provide the summary figure as a separate file in PNG or JPG format at a size of 550x300 pixels (width x height). Please note that the size is rather small and that text needs to be readable at the final size. Please send us this information along with the revised manuscript.

We look forward to seeing a final version of your manuscript as soon as possible.

With kind regards,

Martina Rembold, PhD
Senior Editor
EMBO reports

=================

Referee #1:

The authors have done a great job revising their work. Some of the comments were provided only to the reviewers, and I think that it could be helpful that all points are also addressed clearly in the text. This is really a very minor point, and it should be possible for the authors to finalize this with a short time delay.

Overall, this is really an excellent mansucript with high quality data and a clear message.

Referee #2:

All my comments and suggestions have been successfully addressed by the authors. I have no further remarks.

Referee #3:

My concerns have been suitably addressed. I have no further issues with this paper.

All editorial and formatting issues were resolved by the authors.

Dr. Marek Basler
Basel University
Biozentrum
Spitalstrasse 41
Basel 4056
Switzerland

Dear Dr. Basler,

I am very pleased to accept your manuscript for publication in the next available issue of EMBO reports. Thank you for your contribution to our journal.

Your manuscript will be processed for publication by EMBO Press. It will be copy edited and you will receive page proofs prior to publication. Please note that you will be contacted by Springer Nature Author Services to complete licensing and payment information.

You may qualify for financial assistance for your publication charges - either via a Springer Nature fully open access agreement or an EMBO initiative. Check your eligibility: https://link.springer.com/journal/44319/how-to-publish-with-us

Should you be planning a Press Release on your article, please notify the production team via this form in order to coordinate publication and release dates: https://forms.cloud.microsoft/e/eH4A8JVP6g

If you have any questions, please do not hesitate to contact the Editorial Office. Thank you for your contribution to EMBO Reports.

Yours sincerely,

Achim Breiling
Senior Editor
EMBO Reports

>>> Please note that it is EMBO Reports policy for the transcript of the editorial process (containing referee reports and your response letter) to be published as an online supplement to each paper. If you do NOT want this, you will need to inform the Editorial Office via email immediately. More information is available here: https://link.springer.com/partners/embo-press/editorial-policies#Peer%20review

EMBO Press Author Checklist

Corresponding Author Name: Marek Basler	
Journal Submitted to: EMBO Reports	
Manuscript Number: EMBOR-2026-63869	

USEFUL LINKS FOR COMPLETING THIS FORM
The EMBO Journal - Author Guidelines
EMBO Reports - Author Guidelines
Molecular Systems Biology - Author Guidelines
EMBO Molecular Medicine - Author Guidelines

Reporting Checklist for Life Science Articles (updated January
This checklist is adapted from Materials Design Analysis Reporting (MDAR) Checklist for Authors. MDAR establishes a minimum set of requirements in transparent reporting in the life sciences (see Statement of Task: 10.31222/osf.io/9sm4x). Please follow the journal's guidelines in preparing your
Please note that a copy of this checklist will be published alongside your article.

Abridged guidelines for figures
1. Data
The data shown in figures should satisfy the following conditions:
- the data were obtained and processed according to the field's best practice and are presented to reflect the results of the experiments in an accurate and unbiased manner.
- ideally, figure panels should include only measurements that are directly comparable to each other and obtained with the same assay.
- plots include clearly labeled error bars for independent experiments and sample sizes. Unless justified, error bars should not be shown for technical
- if n<5, the individual data points from each experiment should be plotted. Any statistical test employed should be justified.
- Source Data should be included to report the data underlying figures according to the guidelines set out in the authorship guidelines on Data

2. Captions
Each figure caption should contain the following information, for each panel where they are relevant:
- a specification of the experimental system investigated (eg cell line, species name).
- the assay(s) and method(s) used to carry out the reported observations and measurements.
- an explicit mention of the biological and chemical entity(ies) that are being measured.
- an explicit mention of the biological and chemical entity(ies) that are altered/varied/perturbed in a controlled manner.
- the exact sample size (n) for each experimental group/condition, given as a number, not a range;
- a description of the sample collection allowing the reader to understand whether the samples represent technical or biological replicates (including how many animals, litters, cultures, etc.).
- a statement of how many times the experiment shown was independently replicated in the laboratory.
- definitions of statistical methods and measures:
  - common tests, such as t-test (please specify whether paired vs. unpaired), simple χ2 tests, Wilcoxon and Mann-Whitney tests, can be unambiguously identified by name only, but more complex techniques should be described in the methods section;
  - are tests one-sided or two-sided?
  - are there adjustments for multiple comparisons?
  - exact statistical test results, e.g., P values = x but not P values < x;
  - definition of 'center values' as median or average;
  - definition of error bars as s.d. or s.e.m.

Please complete ALL of the questions below.
Select "Not Applicable" only when the requested information is not relevant for your study.

Materials

Newly Created Materials	Information included in the manuscript?	In which section is the information available? (Reagents and Tools Table, Materials and Methods, Figures, Data Availability Section)
New materials and reagents need to be available; do any restrictions apply?	Yes	Reagents and Tools table

Antibodies	Information included in the manuscript?	In which section is the information available? (Reagents and Tools Table, Materials and Methods, Figures, Data Availability Section)
For **antibodies** provide the following information:<br>- Commercial antibodies: RRID (if possible) or supplier name, catalogue number and or/clone number<br>- Non-commercial: RRID or citation	Yes	Materials and Methods

DNA and RNA sequences	Information included in the manuscript?	In which section is the information available? (Reagents and Tools Table, Materials and Methods, Figures, Data Availability Section)
**Short novel DNA or RNA including primers, probes**: provide the sequences.	Yes	Reagents and Tools table

Cell materials	Information included in the manuscript?	In which section is the information available? (Reagents and Tools Table, Materials and Methods, Figures, Data Availability Section)
**Cell lines:** Provide species information, strain. Provide accession number in repository **OR** supplier name, catalog number, clone number, and/**OR** RRID.	Yes	Reagents and Tools table
**Primary cultures:** Provide species, strain, sex of origin, genetic modification status.	Not Applicable	
Report if the cell lines were recently **authenticated** (e.g., by STR profiling) and tested for mycoplasma contamination.	Yes	Materials and Methods

Experimental animals	Information included in the manuscript?	In which section is the information available? (Reagents and Tools Table, Materials and Methods, Figures, Data Availability Section)
**Laboratory animals or Model organisms:** Provide species, strain, sex, age, genetic modification status. Provide accession number in repository **OR** supplier name, catalog number, clone number, **OR** RRID.	Not Applicable	
**Animal observed in or captured from the field:** Provide species, sex, and age where possible.	Not Applicable	
Please detail **housing and husbandry conditions**.	Not Applicable	

Plants and microbes	Information included in the manuscript?	In which section is the information available? (Reagents and Tools Table, Materials and Methods, Figures, Data Availability Section)
**Plants:** provide species and strain, ecotype and cultivar where relevant, unique accession number if available, and source (including location for collected wild specimens).	Not Applicable	
**Microbes:** provide species and strain, unique accession number if available, and source.	Yes	Reagents and Tools table

Human research participants	Information included in the manuscript?	In which section is the information available? (Reagents and Tools Table, Materials and Methods, Figures, Data Availability Section)
If collected and within the bounds of privacy constraints report on age, sex and gender or ethnicity for all study participants.	Not Applicable	

Core facilities	Information included in the manuscript?	In which section is the information available? (Reagents and Tools Table, Materials and Methods, Figures, Data Availability Section)
If your work benefited from core facilities, was their service mentioned in the acknowledgments section?	Yes	Acknowledgments

Design

Study protocol	Information included in the manuscript?	In which section is the information available? (Reagents and Tools Table, Materials and Methods, Figures, Data Availability Section)
If study protocol has been **pre-registered, provide DOI in the manuscript**. For clinical trials, provide the trial registration number **OR** cite DOI.	Not Applicable	
Report the **clinical trial registration number** (at ClinicalTrials.gov or equivalent), where applicable.	Not Applicable	

Laboratory protocol	Information included in the manuscript?	In which section is the information available? (Reagents and Tools Table, Materials and Methods, Figures, Data Availability Section)
Provide DOI OR other citation details if **external detailed step-by-step protocols** are available.	Not Applicable	

Experimental study design and statistics	Information included in the manuscript?	In which section is the information available? (Reagents and Tools Table, Materials and Methods, Figures, Data Availability Section)
Include a statement about **sample size** estimate even if no statistical methods were used.	Yes	Figure legends
Were any steps taken to minimize the effects of subjective bias when allocating animals/samples to treatment (e.g. **randomization procedure**)? If yes, have they been described?	Not Applicable	
Include a statement about **blinding** even if no blinding was done.	Not Applicable	
Describe **inclusion/exclusion criteria** if samples or animals were excluded from the analysis. Were the criteria pre-established?<br><br>If sample or data points were omitted from analysis, report if this was due to attrition or intentional exclusion and provide justification.	Not Applicable	
For every figure, are **statistical tests** justified as appropriate? Do the data meet the assumptions of the tests (e.g., normal distribution)? Describe any methods used to assess it. Is there an estimate of variation within each group of data? **I**s the variance similar between the groups that are being statistically compared?	Yes	Figure legends and Materials and Methods

Sample definition and in-laboratory replication	Information included in the manuscript?	In which section is the information available? (Reagents and Tools Table, Materials and Methods, Figures, Data Availability Section)
In the figure legends: state number of times the experiment was **replicated** in laboratory.	Yes	Figure legends
In the figure legends: define whether data describe **technical or biological replicates**.	Yes	Figure legends

Ethics

Ethics	Information included in the manuscript?	In which section is the information available? (Reagents and Tools Table, Materials and Methods, Figures, Data Availability Section)
Studies involving **human participants**: State details of **authority granting ethics approval** (IRB or equivalent committee(s), provide reference number for approval.	Not Applicable	
Studies involving **human participants**: Include a statement confirming that **informed consent** was obtained from all subjects and that the experiments conformed to the principles set out in the WMA Declaration of Helsinki and the Department of Health and Human Services Belmont Report.	Not Applicable	
Studies involving **human participants:** For publication of **patient photos**, include a statement confirming that consent to publish was obtained.	Not Applicable	
Studies involving experimental **animals**: State details of **authority granting ethics approval** (IRB or equivalent committee(s), provide reference number for approval. Include a statement of compliance with ethical regulations.	Not Applicable	
Studies involving **specimen and field samples:** State if relevant **permits** obtained, provide details of authority approving study; if none were required, explain why.	Not Applicable	

Dual Use Research of Concern (DURC)	Information included in the manuscript?	In which section is the information available? (Reagents and Tools Table, Materials and Methods, Figures, Data Availability Section)
Could your study fall under dual use research restrictions? Please check biosecurity documents and list of **select agents and toxins** (CDC): https://www.selectagents.gov/sat/list.htm	Not Applicable	
If you used a select agent, is the security level of the lab appropriate and reported in the manuscript?	Not Applicable	
If a study is subject to dual use research of concern regulations, is the name of the **authority granting approval and reference number** for the regulatory approval provided in the manuscript?	Not Applicable	

Reporting

The MDAR framework recommends adoption of discipline-specific guidelines, established and endorsed through community initiatives. Journals have their own policy about requiring specific guidelines and recommendations to complement MDAR.

Adherence to community standards	Information included in the manuscript?	In which section is the information available? (Reagents and Tools Table, Materials and Methods, Figures, Data Availability Section)
State if relevant guidelines or checklists (e.g., **ICMJE, MIBBI, ARRIVE, PRISMA**) have been followed or provided.	Not Applicable	
For **tumor marker prognostic studies,** we recommend that you follow the **REMARK** reporting guidelines (see link list at top right). See author guidelines, under 'Reporting Guidelines'. Please confirm you have followed these guidelines.	Not Applicable	
For **phase II and III randomized controlled trials**, please refer to the **CONSORT** flow diagram (see link list at top right) and submit the CONSORT checklist (see link list at top right) with your submission. See author guidelines, under 'Reporting Guidelines'. Please confirm you have submitted this list.	Not Applicable	

Data Availability

Data availability	Information included in the manuscript?	In which section is the information available? (Reagents and Tools Table, Materials and Methods, Figures, Data Availability Section)
Have **primary datasets** been deposited according to the journal's guidelines (see 'Data Deposition' section) and the respective accession numbers provided in the Data Availability Section?	Yes	Data Availability Section
Were **human clinical and genomic datasets** deposited in a public access-controlled repository in accordance to ethical obligations to the patients and to the applicable consent agreement?	Not Applicable	
Are **computational models** that are central and integral to a study available without restrictions in a machine-readable form? Were the relevant accession numbers or links provided?	Not Applicable	
If publicly available data were reused, provide the respective **data citations in the reference list**.	Not Applicable	
