# Supplementary material for: Regulation of Type VI Secretion Systems of Burkholderia during transition to intracellular lifestyle
Source: EMBO Rep. 2026 Sep 2;27(19):5659–93. doi: 10.1038/s44319-026-00905-2 (PMC13650128; doi:10.1038/s44319-026-00905-2)
Supplement: Supplementary file 4 [file 44319_2026_905_MOESM10_ESM.zip › Movie EV8 legend.docx]

**Movie EV8. *B. thailandensis* ∆*virA* assembles T6SS-1 during infection.** This video shows four segments (Examples 1-4) of *B. thailandensis* ∆*virA* T6SS-1 assemblies inside of A549 cells between 13-16 hpi. Four fields of view (44.45 x 33.34 μm) from independent biological replicates are shown per strain. T6SS-1 is visualized with a TssB-1-msfGFP protein fusion and shown in grayscale. Host cell membrane is stained with CellMask and shown in magenta. The time-lapse video is shown with 10 frames per second. The scale bars represent 5 μm.
