# Supplementary material for: Regulation of Type VI Secretion Systems of Burkholderia during transition to intracellular lifestyle
Source: EMBO Rep. 2026 Sep 2;27(19):5659–93. doi: 10.1038/s44319-026-00905-2 (PMC13650128; doi:10.1038/s44319-026-00905-2)
Supplement: Supplementary file 5 [file 44319_2026_905_MOESM11_ESM.zip › Movie EV9 legend.docx]

**Movie EV9. T6SS-1 assemblies of *B. thailandensis* ∆*tssJLKM-5* during infection.** This video shows three segments (Examples 1-3) of *B. thailandensis* ∆*tssJLKM-5* T6SS-1 assemblies inside of A549 cells between 15-16 hpi. Each field of view is 44.45 x 33.34 μm. T6SS-1 is visualized with a TssB-1-msfGFP protein fusion and shown in grayscale. Host cell membrane is stained with CellMask and shown in magenta. The time-lapse video is shown with 5 frames per second. The scale bars represent 5 μm.
