# Supplementary material for: Regulation of Type VI Secretion Systems of Burkholderia during transition to intracellular lifestyle
Source: EMBO Rep. 2026 Sep 2;27(19):5659–93. doi: 10.1038/s44319-026-00905-2 (PMC13650128; doi:10.1038/s44319-026-00905-2)
Supplement: Supplementary file 6 [file 44319_2026_905_MOESM12_ESM.zip › Movie EV10 legend.docx]

**Movie EV10. T6SS-1 assemblies of *B. thailandensis* ∆T6SS-5 *in vitro*.** This video shows two segments (Examples 1-2) of time-lapse microscopy of *B. thailandensis* strains on agar pads. Each field of view is 26.00 x 19.50 μm. The time-lapse video is shown with 5 frames per second. The scale bars represent 5 μm.
