# Supplementary material for: Regulation of Type VI Secretion Systems of Burkholderia during transition to intracellular lifestyle
Source: EMBO Rep. 2026 Sep 2;27(19):5659–93. doi: 10.1038/s44319-026-00905-2 (PMC13650128; doi:10.1038/s44319-026-00905-2)
Supplement: Supplementary file 16 [file 44319_2026_905_MOESM4_ESM.zip › Movie EV2 legend.docx]

**Movie EV2. T6SS-5 expression in dextran-labelled phagosomes.** This video shows two segments (Examples 1-2) of T6SS-5 expression inside of A549 cells. Each field of view is 32.50 x 24.38 μm. T6SS-5 is visualized with a TssB-5-mScarlet-I protein fusion and shown in grayscale. Bacterial cytosol shown in cyan. Host cells were pre-treated with 0.25 mg/mL of fluorescent dextran for 18 hours (magenta). The time-lapse video is shown with 10 frames per second. The scale bars represent 5 μm.
