# Supplementary material for: Regulation of Type VI Secretion Systems of Burkholderia during transition to intracellular lifestyle
Source: EMBO Rep. 2026 Sep 2;27(19):5659–93. doi: 10.1038/s44319-026-00905-2 (PMC13650128; doi:10.1038/s44319-026-00905-2)
Supplement: Supplementary file 18 [file 44319_2026_905_MOESM6_ESM.zip › Movie EV4 legend.docx]

**Movie EV4. Bafilomycin A1 inhibits T6SS-5 expression.** This video shows four segments of *B. thailandensis* infection of A549 cells either treated with 100nM bafilomycin A1 (BAF) or DMSO. Two fields of view (44.45 x 33.34 μm) from independent biological replicates are shown per condition. T6SS-5 is visualized with a TssB-5-mScarlet-I protein fusion and shown in grayscale. Bacterial cytosol shown in cyan. Host cell membrane is stained with CellMask and shown in magenta. The time-lapse video is shown with 15 frames per second. The scale bars represent 5 μm.
