# Supplementary material for: Regulation of Type VI Secretion Systems of Burkholderia during transition to intracellular lifestyle
Source: EMBO Rep. 2026 Sep 2;27(19):5659–93. doi: 10.1038/s44319-026-00905-2 (PMC13650128; doi:10.1038/s44319-026-00905-2)
Supplement: Supplementary file 19 [file 44319_2026_905_MOESM7_ESM.zip › Movie EV5 legend.docx]

**Movie EV5. T6SS-1 does not assemble during late infection.** This video shows three segments (Examples 1-3) of T6SS-1 dynamics inside of A549 cells at 13 hpi. The three fields of view (43.33 x 32.50 μm) are from independent biological replicates. T6SS-1 is visualized with a TssB-1-msfGFP protein fusion and shown in grayscale. Host cell membrane is stained with CellMask and shown in magenta. The time-lapse video is shown with 10 frames per second. The scale bars represent 5 μm.
