# Supplementary material for: Regulation of Type VI Secretion Systems of Burkholderia during transition to intracellular lifestyle
Source: EMBO Rep. 2026 Sep 2;27(19):5659–93. doi: 10.1038/s44319-026-00905-2 (PMC13650128; doi:10.1038/s44319-026-00905-2)
Supplement: Supplementary file 20 [file 44319_2026_905_MOESM8_ESM.zip › Movie EV6 legend.docx]

**Movie EV6. T6SS-1 assemblies in phagosomes.** This video shows three segments (Examples 1-3) of T6SS-1 dynamics inside of A549 cells between 1-2 hpi. Each field of view is 11.11 x 8.34 μm. T6SS-1 is visualized with a TssB-1-msfGFP protein fusion and shown in grayscale. Host cells were pre-treated with 0.25 mg/mL of fluorescent dextran for 18 hours (magenta). The time-lapse video is shown with 5 frames per second. The scale bars represent 5 μm.
