# Supplementary material for: Regulation of Type VI Secretion Systems of Burkholderia during transition to intracellular lifestyle
Source: EMBO Rep. 2026 Sep 2;27(19):5659–93. doi: 10.1038/s44319-026-00905-2 (PMC13650128; doi:10.1038/s44319-026-00905-2)
Supplement: Supplementary file 21 [file 44319_2026_905_MOESM9_ESM.zip › Movie EV7 legend.docx]

**Movie EV7.** **Inhibition of T6SS-1 assemblies by VirAG *in vitro*.** Time-lapse microscopy of *B. thailandensis* TssB-1-msfGFP parental strain (top row) and a stain harbouring the arabinose-inducible plasmid pmlBAD-VirAG (bottom row) on agar pads. Three fields of view (26.00 x 19.50 μm) from independent biological replicates are shown per strain. The time-lapse video is shown with 6 frames per second. The scale bars represent 5 μm.
